# A Comprehensive HPTLC-Based Analysis of the Impacts of Temperature on the Chemical Properties and Antioxidant Activity of Honey

**DOI:** 10.3390/molecules27238491

**Published:** 2022-12-02

**Authors:** Md Khairul Islam, Tomislav Sostaric, Lee Yong Lim, Katherine Hammer, Cornelia Locher

**Affiliations:** 1 Cooperative Research Centre for Honey Bee Products Limited (CRC HBP), University of Western Australia, Perth 6009, Australia; 2 Division of Pharmacy, School of Allied Health, University of Western Australia, Perth 6009, Australia; 3 School of Biomedical Sciences, University of Western Australia, Perth 6009, Australia

**Keywords:** honey, processing, temperature effect, Manuka, Coastal Peppermint, Marri, HPTLC, quality control

## Abstract

Honeys are commonly subjected to a series of post-harvest processing steps, such as filtration and/or radiation treatment and heating to various temperatures, which might affect their physicochemical properties and bioactivity levels. Therefore, there is a need for robust quality control assessments after honey processing and storage to ensure that the exposure to higher temperatures, for example, does not compromise the honey’s chemical composition and/or antioxidant activity. This paper describes a comprehensive short-term (48 h) and long-term (5 months) study of the effects of temperature (40 °C, 60 °C and 80 °C) on three commercial honeys (Manuka, Marri and Coastal Peppermint) and an artificial honey, using high-performance thin-layer chromatography (HPTLC) analysis. Samples were collected at baseline, at 6 h, 12 h, 24 h and 48 h, and then monthly for five months. Then, they were analysed for potential changes in their organic extract HPTLC fingerprints, in their HPTLC-DPPH total band activities, in their major sugar composition and in their hydroxymethylfurfural (HMF) content. It was found that, while all the assessed parameters changed over the monitoring period, changes were moderate at 40 °C but increased significantly with increasing temperature, especially the honeys’ HPTLC-DPPH total band activity and HMF content.

## 1. Introduction

Honey is a highly concentrated semi-solid natural substance. It mainly consists of sugar (about 65 to 86%), water (about 14–20%) and minor quantities (about 2–3%) of non-sugar components [1,2]. Each constituent class plays a critical role in the honey’s specific characteristics. Moisture gives the honey its viscosity and acts as a dissolution medium for its sugar and non-sugar components [3]. Major sugars such as fructose, glucose, maltose and sucrose, and also minor sugars (e.g., maltotriose, raffinose, erlose, melezitose, turanose), give honey its sweet flavour but also impact on its tendency to crystallise [4,5,6]. Due to its high sugar concentration, the osmolarity of honey is also high, which contributes to its antibacterial properties [7,8,9]. Non-sugar components play a part in honey’s organoleptic properties (e.g., colour, flavour), as well as its antimicrobial and antioxidant activities [10,11].

Raw or unprocessed honey is rarely sold directly to consumers. In most instances, raw honey is subjected to a number of processing steps, such as filtration, heating and/or radiation [12]. Filtration is applied to remove unwanted substances from honey, such as plant debris, bee parts or waxes. Filtration also removes larger sugar crystals that may have already formed during storage and may, if left in the honey, act as seeds for rapid crystallisation [13]. Depending on the pore size, filtration can also remove pollen from honey, a process that, in some instances, has been demonstrated to impact on the honey’s phenolic signature [14]. Filtered honey might thus have a lower antioxidant and optical activity compared to raw or unprocessed honey [14,15]. Radiation is applied to destroy fungal spores, bacterial endospores and other pathogens or microbes in order to sterilise the honey, specifically if it is to be used as a topical wound care product [16]. Usually, high-energy gamma radiation is employed for this purpose [17]. Finally, honeys can be heated to evaporate excess moisture that, otherwise, could facilitate the fermentation and spoilage of honey. Heating also destroys the seed crystals and thus prevents rapid crystallisation and preserves the uniformity of the contents [18,19,20]. Heating also helps to decrease the honey’s viscosity, which facilitates handling, particularly during when dispensing it into jars. Honey is usually exposed to temperatures of 40 to 60 °C for various periods of time during normal processing. However, it can also be treated for shorter periods of time with temperatures as high as 70 or 80 °C to destroy microbial pathogens [18,19,21,22].

The processing of honey, especially heating, can have negative effects. For example, it can lead to the caramelisation of sugars and the formation of unwanted artefacts such as HMF, which is suspected to have carcinogenic effects when ingested in high doses [18,23]. Heating might also change the honey’s native chemical composition, particularly its phenolic profile, which, ultimately, could lead to changes in its antioxidant bioactivity [24]. To maintain its quality and ensure food safety, key honey characteristics, such as its sugar and phenolic profiles [20,21,24], HMF content [2,18,23] and antioxidant properties [12,17], should be monitored during processing steps involving elevated temperatures.

There are several analytical instruments used for honey quality control. For the analysis of sugar and non-sugar components, these include near-infrared spectroscopy (NIR) [25,26], Fourier-transform infrared spectroscopy (FT-IR) [27,28,29], gas chromatography (GC) [30], gas chromatography coupled with mass spectrometry (GC–MS) [31], high-performance liquid chromatography (HPLC) [32,33,34] and nuclear magnetic resonance (NMR) spectroscopy [35,36]. For phenolic compounds, GC–MS and HPLC are commonly used [37], whereas the HMF content is frequently determined by capillary electrophoresis [38] and UV/VIS spectrophotometry [24]. The antioxidant activity in vitro is commonly determined using the DPPH* (2,2-diphenyl-1-picrylhydrazyl), ferric reducing antioxidant power (FRAP) and oxygen radical absorbance capacity (ORAC) assays [39,40,41,42].

In this study, high-performance thin-layer chromatography (HPTLC) was employed for the assessment of all of the above-mentioned honey quality control parameters, demonstrating the versatility of the instrumentation. Islam et al. (2020) developed a fully validated analysis method for sugars in honey using HPTLC, which can detect and quantify its major sugars (e.g., fructose, glucose, sucrose and maltose) with high levels of precision and accuracy, as well as low limits of detection (LOD) and quantification (LOQ) [43]. The method can also be employed for the detection of post-harvest adulterations of honey with sugar syrups [44,45]. Locher et al. (2017 and 2018) developed a HPTLC-based fingerprinting method for organic honey extracts [46,47], which can be used for the authentication of a honey’s floral origin as well as tracking changes in its organic extract profile over time post-exposure to different elevated temperatures. Islam et al. (2020 and 2021) developed a HPTLC–DPPH assay for the measurement of the antioxidant band activities of honey, which can be used to assess changes in its antioxidant activity caused by heating [48]. Along with the HPTLC-based quantification of the HMF in honey [49], these analyses were applied in this study for a comprehensive assessment of the longitudinal effects of temperature on honey quality.

## 2. Results and Discussion

### 2.1. Analysis of Organic Extracts of Honeys

**Figure 1:**
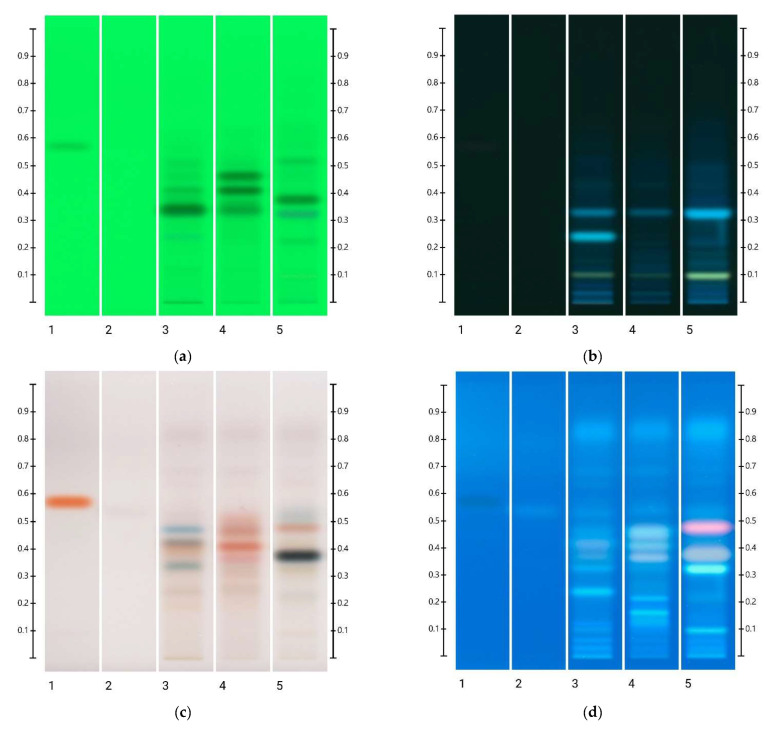
Images taken at (**a**) 254 nm; (**b**) 366 nm; (**c**) white light after derivatisation and (**d**) 366 nm after derivatisation with vanillin reagent; Track 1—4,5,7-trihydroxyflavanon, Track 2—ART, Track 3—LEP, Track 4—MAR, and Track 5—PEP; 5 μL of each honey extract, respectively, all at baseline (0 min).

Figure 1 shows the baseline (0 min) HPTLC fingerprints obtained under four different light conditions (at 245 nm and 366 nm developed; under white light and at 366 nm derivatised) for the organic extracts of the three honeys and the artificial comparator honey. The main features of each set of fingerprints are summarised in Table 1, which stipulates the Rf values of the observed major bands and their respective colours. As these HPTLC fingerprints are reflective of the respective nectar source of each honey [14], it is not surprising that the artificial honey, which constitutes a concentrated sugar solution void of any phenolic compounds or other nectar-derived phytochemicals, lacks any major bands, with the exception of a faint blue band at Rf 0.53, seen at 366 nm derivatised. The major bands recorded for the other three honeys are in agreement with previous findings [41,47].

All four honeys (ART, LEP, MAR and PEP) were exposed over a short-term period (up to 48 h) and also over a five-month period to different temperature conditions. The changes in their respective HPTLC fingerprints over time were recorded, and the major changes that were observed are described below.

The samples kept at ambient temperature (approximately 25 °C) did not present any changes in their HPTLC fingerprints (images not shown), whereas changes did occur in the samples stored at 40 °C, 60 °C and 80 °C. Not all the samples could, however, be tracked over the entire study period, as those kept at 80 °C had already caramelised completely after 48 h. Thus, a continuation of these samples in the long-term study was abandoned.

**Figure 2:**
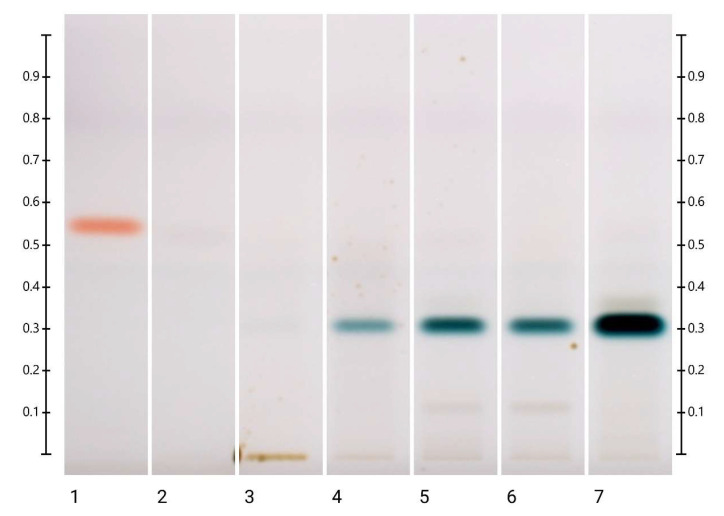
ART long-term storage at 60 °C. Images taken under white light after derivatisation with vanillin reagent; Track 1—4,5,7-trihydroxyflavanon, Track 2—0 h, Track 3—1 month, Track 4—2 months, Track 5—3 months, Track 6—4 months, and Track 7—5 months; 5 μL of each honey extract, respectively.

There were no visible changes in the HPTLC fingerprints of the ART honey at 40 °C after 48 h, the endpoint of the short-term temperature study (Supplementary Figure S1), but the faint blue band at Rf 0.53, seen at 366 nm derivatised, decreased over time at 60 °C and 80 °C. The change was visible at 60 °C after 12 h (Supplementary Figures S2 and S3) and at 80 °C after 6 h (Supplementary Figures S4 and S5) of exposure. A new band at Rf 0.32 could also be detected in the ART sample. It was visible at 40 °C at 254 nm after 2 months and under white light after 4 months (Supplementary Figures S6–S8), and at 60 °C after 1 month and 2 months, respectively (Figure 2 and Supplementary Figures S9–S11). At 80 °C, the band appeared at 254 nm already after 6 h of exposure, and it appeared under white light after 24 h (Supplementary Figure S4). The intensity of this newly emerging band increased over time in all the analytical conditions, indicating the formation of a temperature-induced artefact. Some ‘fuzzy’ bands also appeared at 60 °C at 366 nm after 4 months (Supplementary Figure S9).

There were no visible changes in the HPTLC fingerprints of the LEP honey after 48 h at 40 °C and 60 °C (Supplementary Figures S12 and S13), but the LEP honey stored at 80 °C showed a decrease in the intensity of the bands over time at Rf 0.39 at 254 nm, Rf 0.08 and 0.29 at 366 nm developed, Rf 0.20, 0.40 and 0.46 under white light and Rf 0.20, 0.29, 0.34 and 0.39 at 366 nm derivatised (Supplementary Figures S14–S16). For long term storage at 40 °C, the intensity of the bands at Rf 0.39 at 254 nm, Rf 0.08 and 0.29 at 366 nm developed, Rf 0.20, 0.40 and 0.46 under white light and Rf 0.20, 0.29, 0.34 and 0.39 at 366 nm derivatised decreased (Supplementary Figures S17–S20), but at 60 °C, these reductions in the band intensity were far more pronounced and appeared after a shorter period of exposure (Supplementary Figures S21–S24).

**Figure 3:**
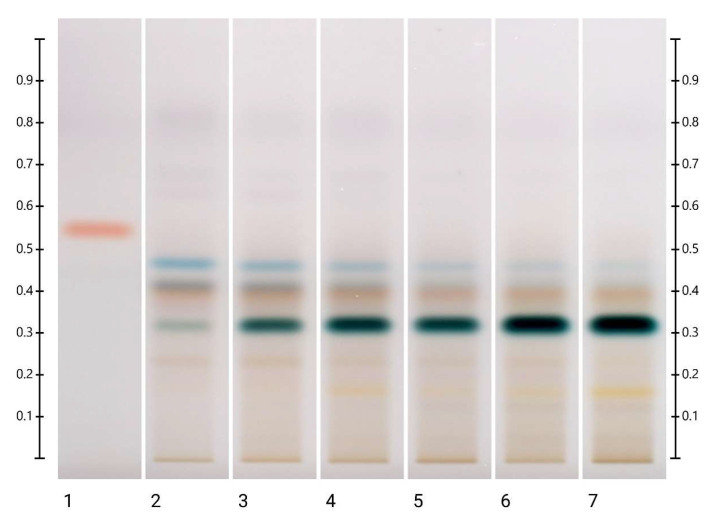
LEP long-term storage at 60 °C. Images taken under white light after derivatisation with vanillin reagent; Track 1—4,5,7-trihydroxyflavanon, Track 2—0 h, Track 3—1 month, Track 4—2 months, Track 5—3 months, Track 6—4 months, and Track 7—5 months; 5 μL of each honey extract, respectively.

A new band also appeared at Rf 0.32 in the LEP honey stored at 40 °C, 60 °C and 80 °C, with its intensity increasing over time. Interestingly, this band coincided with one of the blue bands inherent to LEP. For the samples stored at 40°C, it was visible under white light after 1 month (Supplementary Figure S17). For the samples kept at 60 °C, it was visible at 254 nm, under white light and at 366 nm after 1 month (Figure 3 and Supplementary Figures S11–S24), and for the samples stored at 80 °C, it was visible at 254 nm, under white light and at 366 nm already after 12 h (Supplementary Figures S14–S16).

**Figure 4:**
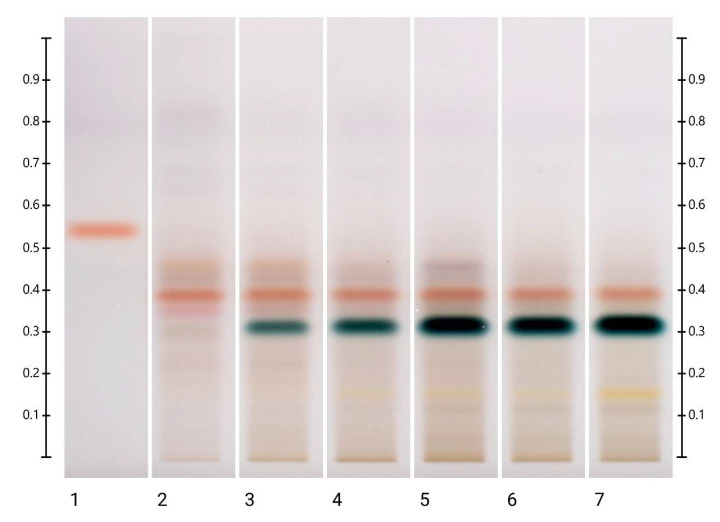
MAR long-term storage at 60 °C. Images taken under white light after derivatisation with vanillin reagent; Track 1—4,5,7-trihydroxyflavanon, Track 2—0 h, Track 3—1 month, Track 4—2 months, Track 5—3 months, Track 6—4 months, and Track 7—5 months; 5 μL of each honey extract, respectively.

There were no visible changes in the HPTLC fingerprints of the MAR honey samples stored at 40 °C and 60 °C during the short-term stability study (Supplementary Figures S25 and S26), but the bands at Rf 0.39 and 0.44 at 254 nm developed, Rf 0.39 and 0.46 under white light and Rf 0.19, 0.34, 0.39 and 0.46 at 366 nm derivatised decreased in intensity over time for the samples stored at 80 °C (Supplementary Figures S27–S30). For long-term storage, at 40 °C, the intensity of the bands at Rf 0.39 and 0.44 at 254 nm developed, Rf 0.46 under white light and Rf 0.46 at 366 nm derivatised decreased (Supplementary Figures S31–S34), and these reductions in the band intensity were more noticeable in the MAR samples kept at 60 °C. This can be seen, for example, in the bands at Rf 0.44 at 254 nm developed, Rf 0.46 under white light and Rf 0.46 at 366 nm derivatised, which reduced in intensity after 2 months of storage (Figure 4 and Supplementary Figures S35–S38).

Similar to what was observed in both the ART and LEP samples, a new band at Rf 0.32 appeared and increased in intensity over time in the MAR samples stored at 40 °C and 60 °C in the long-term study and in the MAR samples kept at 80 °C in the short-term study. For the samples stored at 40 °C, the band was visible under white light after 1 month (Supplementary Figure S31). For the samples stored at 60 °C, it appeared at 254 nm, under white light and at 366 nm after 1 month (Figure 4 and Supplementary Figures S35–S38), and for the samples stored at 80 °C, it was visible already after 12 h (Figure 4 and Supplementary Figures S27–S30).

**Figure 5:**
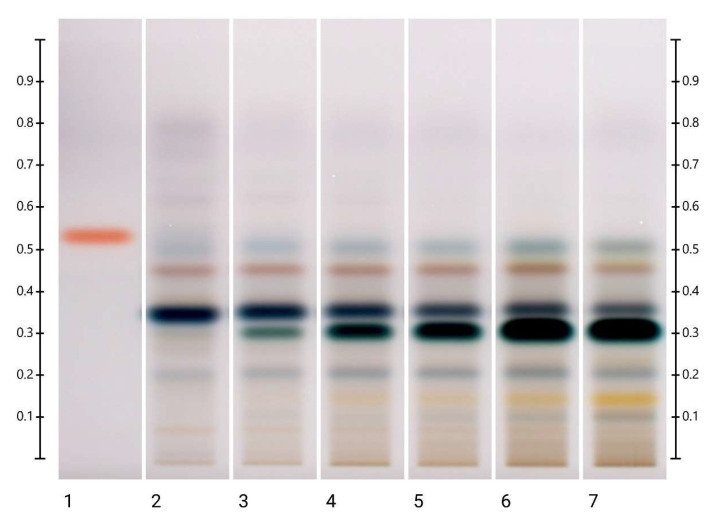
PEP long-term storage at 60 °C. Images taken under white light after derivatisation with vanillin reagent; Track 1—4,5,7-trihydroxyflavanon, Track 2—0 h, Track 3—1 month, Track 4—2 months, Track 5—3 months, Track 6—4 months, and Track 7—5 months; 5 μL of each honey extract, respectively.

There were no visible changes in the HPTLC fingerprints of the PEP honey at 40 °C, 60 °C and 80 °C after 48 h (Supplementary Figures S39–S41). For long-term storage at 40 °C, there were no significant changes in the intensity of the bands (Supplementary Figures S44–S47), but at 60 °C, the bands at Rf 0.50 at 254 nm developed and at Rf 0.47 and 0.50 under white light derivatised increased in intensity, whereas the bands at Rf 0.36 at 254 nm developed, Rf 0.36 under white light and Rf 0.36 and 0.47 at 366 nm derivatised decreased in intensity (Figure 5 and Supplementary Figures S48–S51). New bands also appeared at Rf 0.47 at 254 nm developed and at Rf 0.32 at 254 nm developed, under white light and at 366 nm derivatised. The appearance of the latter was dependent on the storage conditions. It emerged at 40 °C after 2 months, at 60 °C after 1 month and at 80 °C after 24 h, as seen especially under white light (Figure 5 and Supplementary Figures S44 and S48).

In summary, the short-term storage (up to 48 h) of the honeys at 40 °C and 60 °C did not seem to cause any changes in their organic extract fingerprints. In contrast, at 80 °C, changes in the organic extract fingerprints were observed as early as after only 6 h of storage (Table 2). The long-term storage of the honeys at 40 °C and 60 °C caused changes in their organic extract fingerprints. At 40 °C, the changes were noticeable after one or two months and at 60 °C from one month onwards. These changes could be seen either as a decrease in the intensity of certain bands present in the honeys or as the appearance of new bands. Of particular interest in this context, thus warranting further investigation, is the honey artefact at Rf 0.32, which seems to have formed across all the honeys, as well as the ART.

### 2.2. Analysis of an Unidentified Honey Artefact Formed during Storage under Extreme Conditions

**Figure 6:**
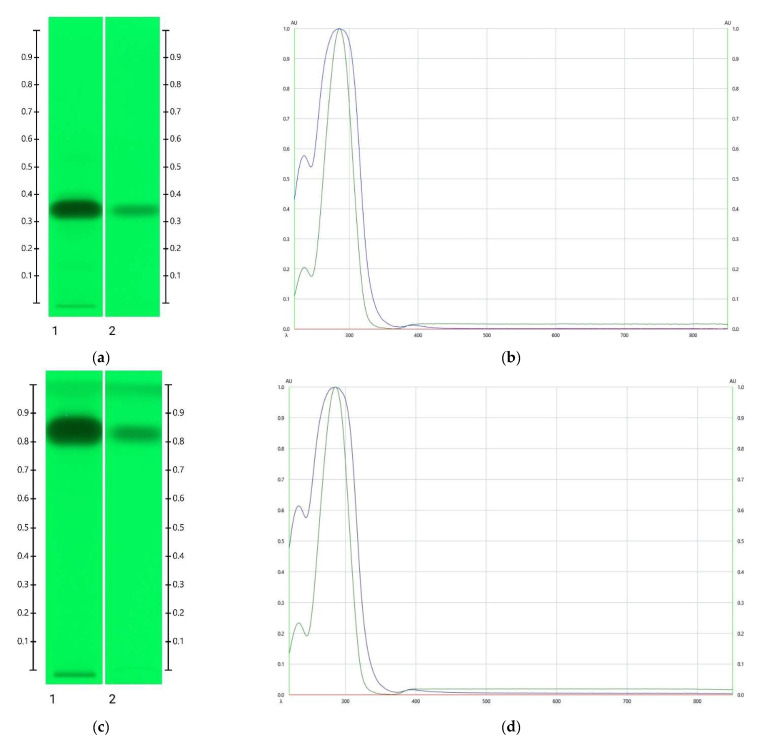
HPTLC images taken at 245 nm after development with toluene: ethyl acetate: formic acid (6:5:1, *v*/*v*/*v*) (**a**); corresponding absorbance spectra (**b**); development with ethyl acetate (**c**); corresponding absorbance spectra (**d**); Track 1— ART short-term storage at 60 °C for 5 months (5 μL) (blue line in spectral analysis), Track 2—HMF (1 mg/mL) aqueous solution (2 μL) (green line in spectral analysis).

**Figure 7:**
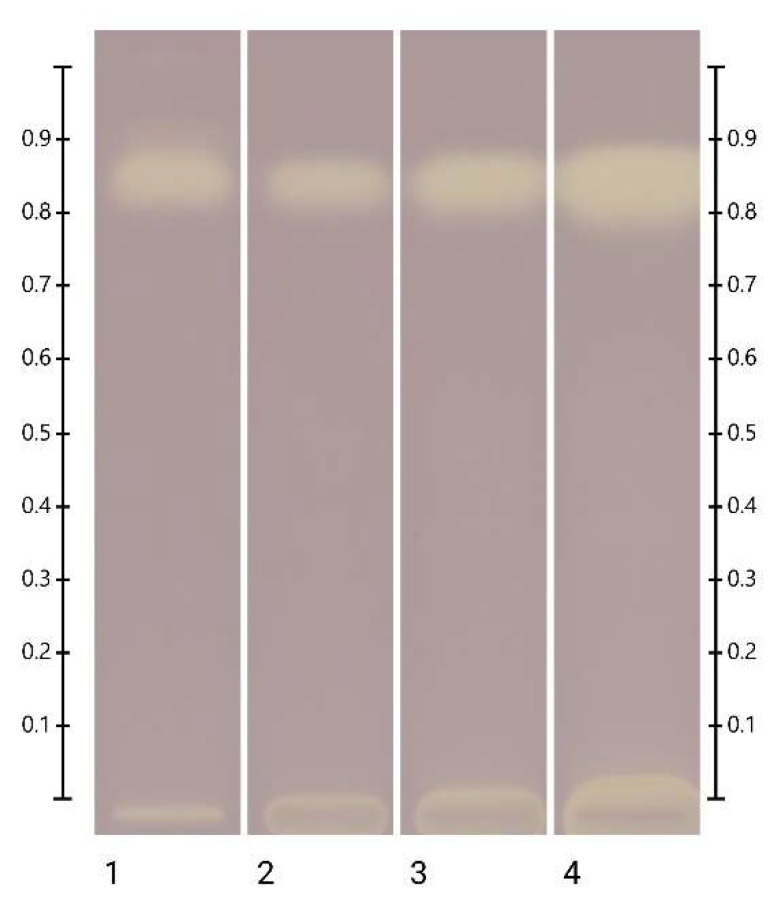
Images of HPTLC plate taken under white light 60 min after derivatisation with DPPH* reagent; Track 1— ART short-term storage at 60 °C for 5 months (5 μL), Track 2—HMF (20 mg/mL) aqueous solution (3 μL), Track 3—HMF (20 mg/mL) aqueous solution (5 μL), and Track 4—HMF (20 mg/mL) aqueous solution (10 μL).

The spectral analysis of the unknown band at Rf 0.32 was carried out using a CAMAG TLC Scanner 4 in the absorbance (220–850 nm) mode. As the intensity of that particular band for the ART stored for 5 months at 60 °C was highest and without the interference of any additional bands, this ART sample was used for the spectral analysis. After development, the absorbance maximum was found at 286 nm (Figure 6). A comparative run was performed in two different mobile phases (toluene: ethyl acetate: formic acid (6:5:1, *v*/*v*/*v*) and ethyl acetate) using hydroxymethylfurfural (HMF) as a reference. The Rf values and spectra of the unidentified honey artefact were found to match that of HMF. Furthermore, a comparative run was conducted with HMF, followed by derivatisation with DPPH (see Section 2.3), and antioxidant activity was detected for both bands (Figure 7). It can therefore be concluded that the honey artefact is HMF.

### 2.3. Analysis of HPTLC-DPPH Activities of the Organic Extracts of the Honeys

Changes in antioxidant activity on exposure to different temperatures over time, reflected in the respective HPTLC-DPPH fingerprints, were recorded for all three honeys (LEP, MAR, PEP), as well as the artificial honey (ART). The samples were analysed under white light 1 h after derivatisation with DPPH* reagent, with any bands exhibiting antioxidant activity reacting with the reagent, thus showing a change from its inherent purple colour to yellow. The formation of any yellow bands is thus indicative of antioxidant activity, and the intensity of the yellow colour correlates with the intensity of the effect [48,50]. Major changes seen in the four samples on exposure to the four temperature conditions over five months are described below.

The honey samples stored at ambient temperature (approximately 25 °C) did not show any changes in their respective HPTLC-DPPH fingerprints (data not presented). When stored at 40 °C, 60 °C and 80 °C, the antioxidant band activities of the honeys changed over time, and for the samples stored at 80 °C, it was impossible to record any data beyond 48 h exposure due to the caramelisation of the samples.

**Figure 8:**
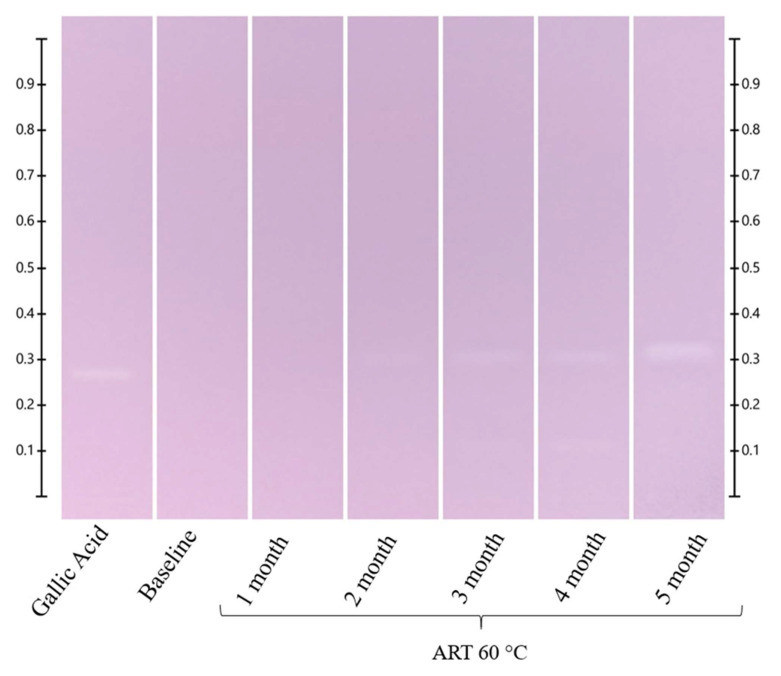
HPTLC-DPPH fingerprints of ART honey stored at 60 °C for up to 5 months. Images of the HPTLC plate were taken under white light after 60 min of derivatization with DPPH* reagents, gallic acid (4 μL) and honey extracts (5 μL) respectively.

As expected, there were no visible changes in the HPTLC-DPPH fingerprints of the ART honey at 40 °C, 60 °C and 80 °C during the short-term study (Supplementary Figures S52 and S53). Storage at 40 °C for up to 5 months also did not result in the formation of any antioxidant bands (Supplementary Figures S54a and S55). However, a faint band with antioxidant activity started to appear at Rf 0.32 after two months in the sample kept at 60 °C (Figure 8 and Supplementary Figure S56). Interestingly, the Rf value of this band corresponds to that of the heat-induced artefact detected by HPTLC that emerged in the ART and all the other honeys over time, particularly on exposure to higher temperatures.

**Figure 9:**
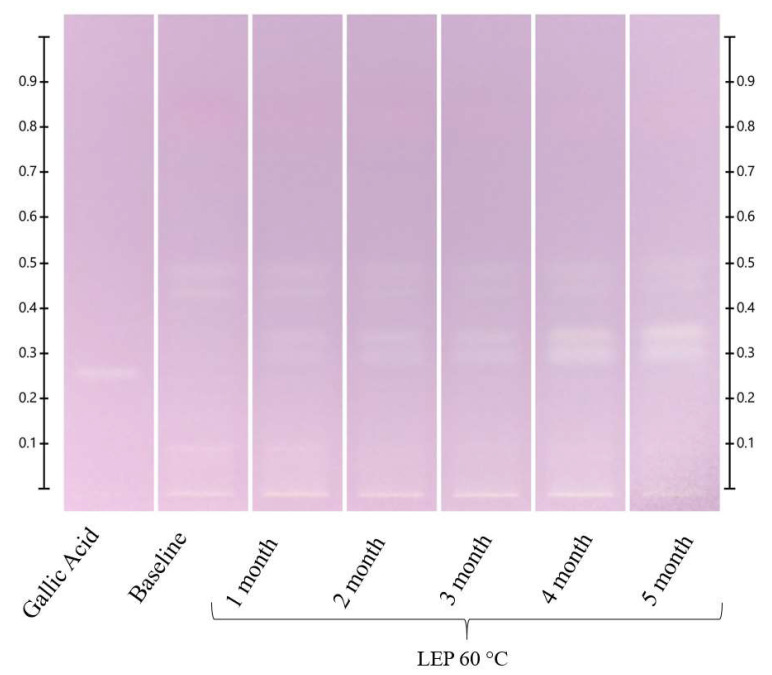
HPTLC-DPPH fingerprints of LEP honey stored at 60 °C for up to 5 months. Images of the HPTLC plate were taken under white light after 60 min of derivatization with DPPH* reagents, gallic acid (4 μL) and honey extracts (5 μL), respectively.

During the short-term study, there were no visible changes in the HPTLC-DPPH fingerprints of the LEP honey at 40 °C and 60 °C (Supplementary Figures S57 and S58), but a faint antioxidant band appeared at Rf 0.4 after 6 h of storage at 80 °C. On the other hand, during the long-term study, it was noted that the antioxidant activity of two bands (at Rf 0.46 and 0.49) inherent to the LEP sample decreased following storage at both 40 °C and 60 °C, whereas two additional antioxidant bands (at Rf 0.32 and 0.35) appeared after 4 months of storage at 40 °C and after 1 month of storage at 60 °C. The intensity of these bands continued to increase as the storage duration progressed (Figure 9 and Supplementary Figures S59–S61).

**Figure 10:**
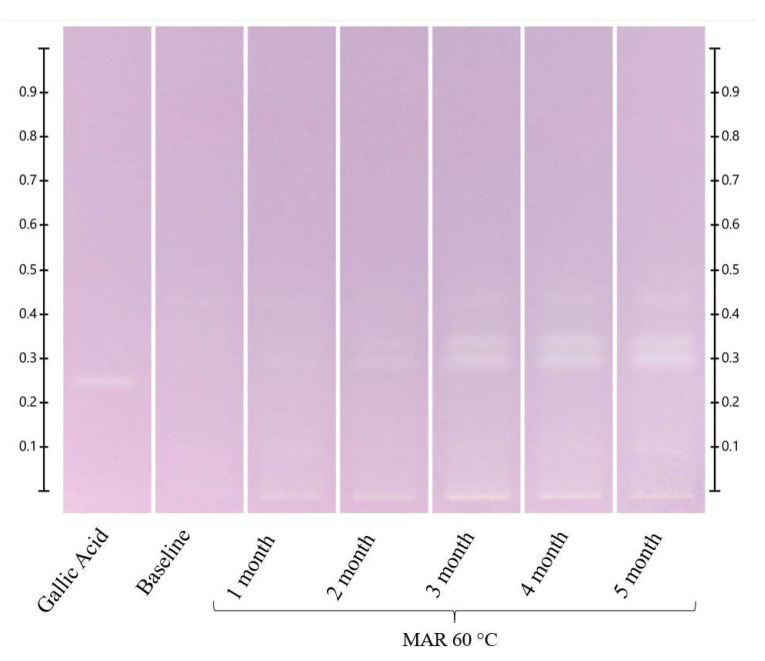
HPTLC-DPPH fingerprints of MAR honey stored at 60 °C for up to 5 months. Images of the HPTLC plate were taken under white light after 60 min of derivatization with DPPH* reagents, gallic acid (4 μL) and honey extracts (5 μL), respectively.

Similar to the findings observed for the LEP, there were also no visible changes in the HPTLC-DPPH fingerprints of the MAR honey at 40 °C and 60 °C during the short-term study (Supplementary Figures S62 and S63), but a faint antioxidant band appeared at Rf 0.39 in the sample stored at 80 °C after 24 h. During the long-term study, it was found that the antioxidant activity of the band at Rf 0.43 decreased when the samples were stored at both 40 °C and 60 °C, and two additional bands (at Rf 0.32 and 0.35) appeared after 4 months storage at 40 °C and after 1 month storage at 60 °C. The intensity of these bands continued to increase with the progression of the storage duration (Figure 10 and Supplementary Figures S64–S66).

**Figure 11:**
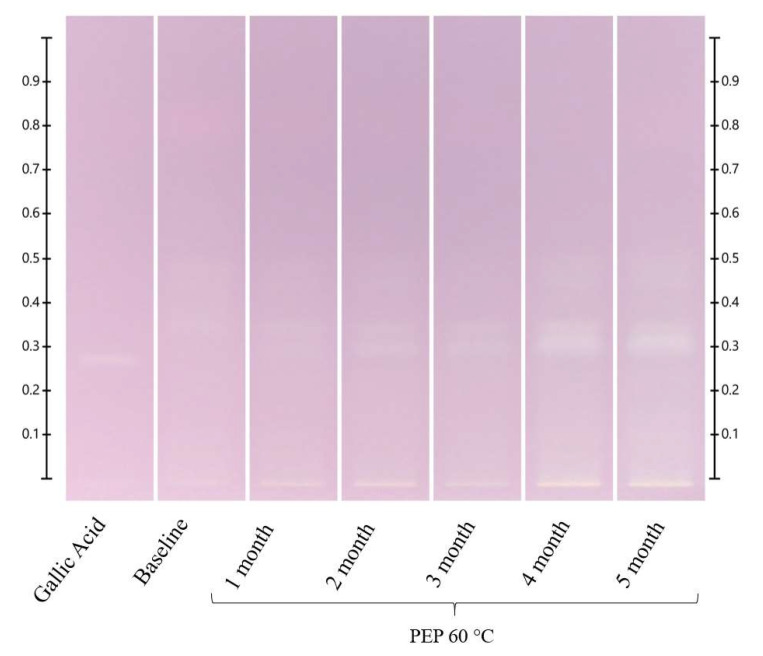
HPTLC-DPPH fingerprints of PEP honey stored at 60 °C for up to 5 months. Images of the HPTLC plate were taken under white light after 60 min of derivatization with DPPH* reagents, gallic acid (4 μL) and honey extracts (5 μL), respectively.

For the PEP samples, no visible changes in the HPTLC-DPPH fingerprints were detected in the short-term study for the samples stored at 40 °C and 60 °C (Supplementary Figures S67 and S68), but a faint band appeared at Rf 0.39 after 24 h storage at 80 °C. In the long-term study, two additional antioxidant bands (at Rf 0.32 and 0.35) appeared after 4 months of storage at 40 °C and after 1 month of storage at 60 °C. As was seen in the other honeys, these newly formed bands continued to increase in intensity with the prolongation of the storage time (Figure 11 and Supplementary Figures S69–S71).

The investigation of the effects of elevated temperatures on the antioxidant activity presented a somewhat complex picture, as some of the antioxidant compounds inherent to the honey were negatively affected, whereas temperature-induced artefacts with antioxidant activities also emerged in some cases. One of these (Rf 0.32) appears to be related to the honeys’ sugar fraction, as it was detected in the honey samples themselves (LEP, MAR, PEP) and also in the artificial honey (ART).

In a similar way, it was found that the antioxidant band activities of the three honeys were quite stable at 40 °C and 60 °C throughout the short-term study. When stored for 5 months at 40 °C and 60 °C, significant changes in the antioxidant band activities were noted, which were also associated with the formation of a honey artefact with antioxidant activity. The same pattern emerged for the honeys stored at 80 °C, albeit over a much shorter time frame.

A previous study suggested that the degradation of sugars produces Maillard reaction products which are non-nutrient antioxidants [51,52]. This study confirmed the formation of hydroxymethylfurfural (HMF), which is a Maillard reaction product. However, although HMF has been found to possess DPPH antioxidant activity, it is also known to be harmful to human health [18,23]. This demonstrates that some caution is warranted before claims regarding potential health benefits are made based on the determination of the total DPPH antioxidant activity of honey. Future studies should therefore investigate temperature-induced honey artefacts in more depth and determine their contributions to not only the honey’s overall antioxidant activity but also its impacts on human health.

### 2.4. Analysis of the Major Sugars of the Honeys

**Figure 12:**
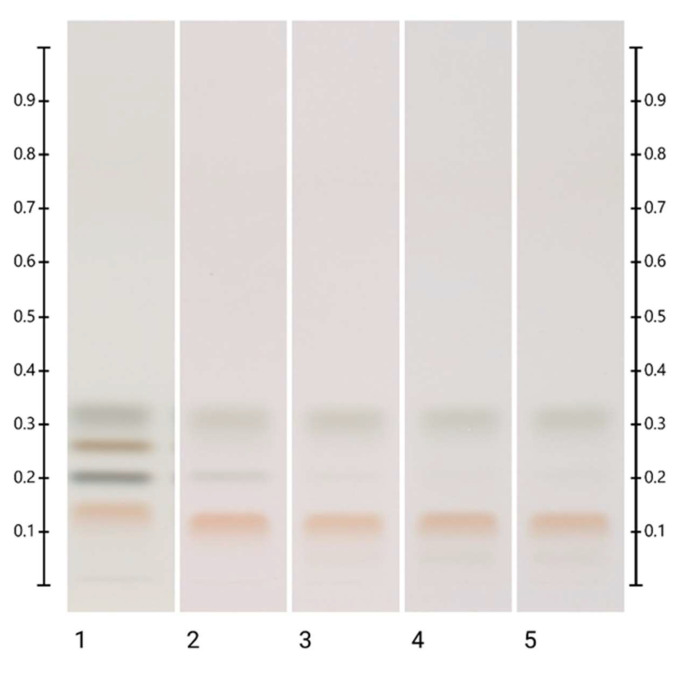
HPTLC images taken under white light after derivatisation with aniline-diphenylamine-phosphoric acid reagent; Track 1—standards (fructose, maltose, sucrose and glucose in increasing Rf values), Track 2—ART, Track 3—MAN, Track 4—MAR, Track 5—PEP; 2 µL of aqueous methanolic solution each.

The presence of fructose, glucose, maltose and sucrose was analysed by HPTLC in all the honeys, including the artificial honey (Figure 12). LEP, MAR and PEP were found to contain detectable quantities of fructose and glucose only. As the artificial honey (ART) was prepared by mixing fructose, glucose, maltose and sucrose, the presence of all four sugars was confirmed, and their respective quantities were determined. With recoveries of 98% (fructose) and 97% (glucose), the precision of the validated analysis method [43,44] used to detect major sugars in honey was confirmed once more (Table 3). As the fructose to glucose ratio (F/G) is an important honey characteristic, which not only influences its crystallisation behaviour but can also be used for honey authentication [44], this ratio was determined for all the honeys and found to be within the expected ranges for those honeys for which published information on their F/G ratio was available [44].

To monitor the potential impact of temperature on the sugar composition of the honey, the respective F/G ratios of the three honeys and the artificial honey comparator were tracked over time during the short- and long-term stability studies (Table 4).

Storage at ambient temperature (approximately 25 °C) did not trigger any changes in the F/G ratios of all the samples over the entire analysis period (data not presented). Changes only occurred at 40 °C, 60 °C and 80 °C, although, in the latter case, they could only be visually detected but not quantitatively analysed, as the samples were already completely caramelized after 48 h. Thus, data for storage at 80 °C for 48 h is not included here.

In the short-term study, there were no statistically significant differences in the F/G ratios compared to baseline (0 min) for all the samples stored under the three temperature conditions (at 40 °C, 60 °C and 80 °C). In the long-term study, for storage at 40 °C, there were no statistically significant differences in the F/G ratio compared to baseline for the ART, LEP and MAR honeys. However, in the PEP honey, a statistically significant difference was found after 5 months of storage (Table 4). For the PEP honey stored at 60 °C, there was also a statistically significant difference in the F/G ratio at 5 months, the same time point seen in the case of storage at 40 °C. For the ART and LEP honeys stored at 60 °C, statistically significant differences could be seen from 3 months onwards, whereas for the MAR honey, statistically significant changes could be seen after 2 months of storage at that temperature.

### 2.5. Analysis of 5-Hydroxymethylfurfural (HMF) in the Honeys

The HMF levels at baseline (0 min) were quantified by HPTLC at 290 nm using the instrument module’s TLC scanner. According to the Codex Alimentarius Commission’s guidelines, acceptable HMF concentrations are those below 40 mg/kg or below 80 mg/kg for honeys produced in tropical regions. Of the honeys used in this study, ART and PEP did not have detectable quantities of HMF at baseline. The HMF level of MAR at the time of the commencement of the study was within the acceptable ranges, but in the LEP honey, the baseline HMF level was found to already exceed the Codex Alimentarius guidelines [53], even if the higher threshold of 80 mg/kg was applied (Table 5). As the HMF content of LEP was already above the acceptable limits, the honey was not included in the HMF content analysis as part of the short- and long-term stability study.

During storage at ambient temperature (approx. 25 °C), no changes in the HMF content could be detected in any of the samples (data not presented). However, the samples kept at 40 °C and 60 °C showed increases in the HMF content, whereas the samples stored at 80 °C could not be analysed beyond 48 h due to their complete caramelisation. Samples were excluded from the experiment as soon as their HMF content was found to exceed the 80 mg/kg content threshold.

In the ART honey, detectable quantities of HMF were noticed after two months of storage at 40 °C, with an average of 7.25 mg/kg. At 60 °C storage, HMF was detectable after one month, and the average content of 91.36 mg/kg was already above the acceptable upper limits. At 80 °C, HMF was recorded after 24 h of storage, yielding, on average, 9.99 mg/kg; however, a rapid increase with the storage time was noted, with the HMF content after 48 h determined to be 119.64 mg/kg, which exceeded acceptable limits.

For the MAR honey, the detectable amount of HMF present at baseline (36.75 mg/kg on average) remained unchanged when the samples were stored at 40 °C, with the samples stored at 48 h showing, on average, 43.53 mg/kg HMF. Interestingly, the HMF concentrations then decreased over the first storage month (33.18 mg/kg) before rising again and exceeding the acceptable limits after two months of storage when, on average, 112.64 mg/kg HMF was found in the samples. At 60 °C, the HMF content gradually increased to 63.48 mg/kg after 48 h and had already exceeded acceptable limits after 1 month of storage at that temperature, with an average of 259.55 mg/kg. At 80 °C, the formation of HMF was even faster, reaching an average of 67.61 mg/kg after 6 h, and it exceeded the acceptable limits after 12 h, with an average HMF content of 123.98 mg/kg.

**Figure 13:**
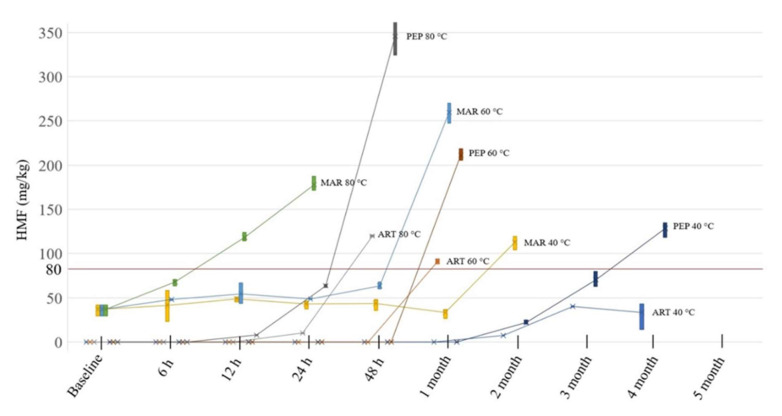
5-Hydroxymethylfurfural (HMF) content in different honeys over time.

For the PEP honey, at 40 °C storage, detectable quantities of HMF were observed after 2 months, with an average HMF content of 21.8 mg/kg. After 3 months, the HMF level had climbed on average to 70.33 mg/kg and exceeded the acceptable limits after 4 months of storage (128.34 mg/kg HMF on average). For PEP stored at 60 °C, there was no detectable quantity of HMF after 48 h, but after 1 month of storage, the HMF content had already exceeded the acceptable limits, with the samples showing an average HMF content of 211.24 mg/kg. For PEP stored at 80 °C, HMF could already be detected after 12 h, with an average of 7.68 mg/kg. After 24 h, the average increased to 63.60 mg/kg, and after 48 h, the samples had an average HMF content (345.64 mg/kg) that exceeded the acceptable limits (Figure 13).

As the floral origins of the analysed honeys were different, their initial HMF contents varied. Furthermore, differences in the HMF content at baseline might also be reflective of potential exposure to heat as a result of processing and storage prior to purchase. The ART and PEP honey samples did not have any detectable levels of HMF at baseline. The MAR honey had detectable levels, but its HMF content was within the limits set by the Codex Alimentarius guidelines, whereas the investigated LEP honey already had an HMF content exceeding the acceptable limits.

Given the inherent differences at baseline among LEP, MAR and PEP, the ART honey is the most appropriate sample with which to discuss general changes in the HMF content that can be expected on temperature exposure. At 40 °C storage, HMF was detected in the ART honey after two months, while at 60 °C, there were no detectable quantities of HMF during short-term storage (up to 48 h), but acceptable limits were exceeded after one month (average 91.36 mg/kg). At 80 °C storage, the ART honey’s HMF content was within the acceptable limits for up to 24 h of storage (average 9.99 mg/kg) but exceeded the acceptable range at 48 h (average 119.36 mg/kg). Based on these findings, if nectar-derived honeys have no detectable HMF at baseline, it can be concluded that they can be safely stored at 40 °C for up to two months, at 60 °C for less than one month and at 80°C not even for 48 h. The storage times will be shorter for honeys that already have an inherent HMF content at baseline.

## 3. Materials and Methods

### 3.1. Experimental Design

The honey samples (Table 5) were placed in glass-stoppered glass jars and stored at ambient temperature (approx. 25 °C) and also at 40 °C, 60 °C and 80 °C using a temperature-controlled oven (Memmert GmbH + Co. KG, Büchenbach, Germany). For the short-term stability study, sampling was carried out in triplicate (*n* = 3) at 0 min, 6 h, 12 h, 24 h and 48 h. For the long-term stability study, sampling was carried out at baseline (0 min) and then monthly for five months. As the samples stored at 80 °C changed rapidly and already appeared to be completely caramelised and dark-coloured after a few days, the 80 °C storage condition was excluded from the long-term stability study.

The collected honey samples were extracted with dichloromethane, and their HPTLC fingerprints were recorded (see Section 3.4.1) alongside their antioxidant profiles (see Section 3.4.2), which allowed us to track the antioxidant activity of the active bands. Aqueous methanolic honey solutions were also prepared and analysed by HPTLC to record their major sugar profiles and to quantify their main sugars (see Section 3.4.3). The content of HMF in the aqueous honey samples was also recorded by HPTLC analysis (see Section 3.4.4).

### 3.2. Chemicals and Reagents

The chemicals and reagents and their suppliers were as follows: glucose, sucrose (Chem-Supply Pty Ltd., St. Gillman, SA, Australia), fructose, maltose, aniline, vanillin (Sigma-Aldrich, St. Louis, MO, USA), boric acid (Pharma Scope, Welshpool, WA, Australia), 4,5,7-trihydroxyflavanone, 5-hydroxymethylfurfural (Alfa Aesar, England, UK), DPPH* (Fluka AG, Buchs SG, Switzerland), gallic acid, diphenylamine, phosphoric acid (Ajax Finechem Pvt Ltd., Sydney, Australia), anhydrous magnesium sulphate (Scharlau, Barcelona, Spain) and Folin and Ciocalteu’s Phenol Reagent 2N (Sigma-Aldrich, St. Louis, MO, USA).

The solvents and their suppliers were as follows: methanol (Scharlau, Barcelona, Spain), 1-butanol (Chem-Supply Pty Ltd., St. Gillman, SA, Australia), 2-propanol (Asia Pacific Specialty Chemicals Ltd., Sydney, Australia), dichloromethane (Merck KGaA, Darmstadt, Germany), toluene (APS Chemicals, Sydney, Australia), ethanol, ethyl acetate and formic acid (Ajax Finechem Pvt Ltd., Sydney, Australia).

The commercial honeys (Table 6) were obtained from beekeepers and supermarkets in Western Australia. An artificial honey was prepared as the comparator honey (see Section 3.3.2).

### 3.3. Sample Preparation

#### 3.3.1. Standards, Reagents and Mobile Phase Preparation

For the organic extract HPTLC fingerprinting, a methanolic solution of 0.5 mg/mL of 4,5,7-trihydroxyflavanone was prepared as a reference standard. A mixture of toluene: ethyl acetate: formic acid (6:5:1, *v*/*v*/*v*) was used as the mobile phase. The vanillin derivatisation reagent was prepared by dissolving 1 g of vanillin in 100 mL of ethanol, followed by the dropwise addition of 2 mL of sulphuric acid. The antioxidant derivatisation reagent was prepared by dissolving 40 mg DPPH* in 10 mL of a mixture of 50% methanol and 50% ethanol and stored in an amber glass bottle, which was protected from light until further use.

To identify and quantify the honey’s main sugars, standard glucose, fructose, maltose and sucrose solutions (250 μg/mL) were prepared by dissolving 25 mg of the respective sugar in 100 mL of 50% aqueous methanol. A 3:5:1 *v*/*v*/*v* mixture of 1-butanol: 2-propanol: boric acid (5 mg/mL in water) was used as the mobile phase. The derivatisation reagent was prepared by dissolving 2 g of diphenylamine and 2 mL of aniline in 80 mL of methanol. After the addition of 10 mL of phosphoric acid (85%), the solution was made up to 100 mL using methanol.

To detect and quantify the presence of HMF, an aqueous 0.01% (*w*/*v*) solution of HMF was prepared as the standard. Ethyl acetate was used as the mobile phase.

#### 3.3.2. Preparation of the Samples for Analysis

The artificial honey (ART) was prepared as described previously [54] by dissolving 40.5 g fructose, 33.5 g glucose, 1.5 g sucrose and 7.5 g maltose in 17 mL of deionised water.

For the preparation of the organic honey extracts, approximately 1 g of honey was mixed with 2 mL of deionised water. The aqueous solution was then extracted three times with 5 mL of dichloromethane. The combined organic extracts were dried with anhydrous MgSO_4_ and filtered, and the solvent was evaporated at ambient temperature. The extract was stored at 4 °C and reconstituted in 100 µL dichloromethane prior to HPTLC analysis.

For the sugar analysis, 100 mg of honey was dissolved in 80 mL of 50% aqueous methanol by sonication and then made up to 100 mL with 50% aqueous methanol.

A 10% (*w*/*v*) aqueous solution of honey was used for the analysis of its HMF content.

### 3.4. Instrumentation and High-Performance Thin-Layer Chromatography (HPTLC) Method

#### 3.4.1. Organic Extract Analysis

The reference standard (4 µL) and the respective organic honey extract solutions (5 µL) were applied as 8 mm bands at 8 mm from the lower edge of the HPTLC plate (glass plates 20 × 10 cm, silica gel 60 F_254_) at a rate of 150 nLs^−1^ using a semi-automated HPTLC application device (Linomat 5, CAMAG). The chromatographic separation was performed in a saturated and activated (33% relative humidity) automated development chamber (ADC2, CAMAG). The plates were pre-conditioned with the mobile phase for 5 min and automatically developed to a distance of 70 mm at a fixed ambient temperature. The obtained chromatographic results were documented using an HPTLC imaging device (TLC Visualizer 2, CAMAG) at 254 nm and 366 nm, respectively.

After the initial documentation of the chromatographic results, each plate was derivatised with 3 mL of vanillin reagent and heated for 3 min at 115 °C using a CAMAG TLC Plate Heater III. The plate was cooled to room temperature and analysed with the HPTLC imaging device under white light and at 366 nm [46,47]. The chromatographic images were digitally processed and analysed using specialised HPTLC software (visionCATS, CAMAG), which was also used to control the individual instrumentation modules.

#### 3.4.2. HPTLC-DPPH Fingerprint Analysis

For the quantification of the antioxidant constituents as gallic acid equivalents in the respective honeys’ organic extracts, 4 µL of the reference solution, 4 µL of the gallic acid standard solution and 5 µL of the respective honey extract were applied as 8 mm bands at 8 mm from the lower edge of the HPTLC plate (glass plates 20 × 10 cm, silica gel 60 F254) at a rate of 150 nLs^−1^ using a semi-automated HPTLC application device (Linomat 5, CAMAG). To prepare a gallic acid standard curve in the honey matrix, 2 µL, 3 µL, 4 µL, 5 µL, 6 µL and 7 µL of gallic acid standard solution were applied by over-spotting the honey bands.

The chromatographic separation was performed in a saturated and activated (33% relative humidity) automated development chamber (ADC2, CAMAG), and the plates were pre-conditioned with the mobile phase for 5 min and automatically developed to a distance of 70 mm at a fixed ambient temperature. The plates were then dried for 5 min before being derivatised with 3 mL of 0.4% DPPH* reagent (CAMAG derivatiser). The derivatised plates were analysed with the HPTLC imaging device under white light by taking images at 60 min after their derivatisation [48,50]. The obtained chromatographic images were digitally processed and analysed using specialised HPTLC software (visionCATS, CAMAG), which was also used to control the individual instrumentation modules. For the quantification of the honey’s antioxidant constituents as gallic acid equivalents, the obtained images were converted into individual absorbance points according to their Rf values. Using Excel^©^, the data were converted into chromatograms, which were used to derive calibration curves of the area of absorbance versus concentration [50].

#### 3.4.3. Sugar Analysis

The standard solutions were applied as 8 mm bands at 8 mm from the lower edge of the HPTLC plate (glass plates 20 × 10 cm, silica gel 60 F_254_) at a rate of 50 nLs^−1^ using a semi-automated HPTLC application device (Linomat 5, CAMAG). To prepare the glucose, fructose, sucrose and maltose standard curves, 1 µL, 2 µL, 3 µL, 4 µL and 5 µL of the respective standard solutions were applied. For the analysis of the sugars in the honey samples, 2 µL of the respective sample solution was applied.

The chromatographic separation was performed in a saturated (33% relative humidity) automated development chamber (ADC2, CAMAG). The development chamber was saturated for 60 min, and the plates were pre-conditioned with the mobile phase for 5 min, automatically developed to a distance of 85 mm at a fixed ambient temperature and dried for 5 min. The obtained chromatographic results were documented using an HPTLC imaging device (TLC Visualizer 2, CAMAG) under white light.

After the initial documentation of the chromatographic results, each plate was derivatised with 2 mL of aniline-diphenylamine-phosphoric acid reagent using a TLC derivatiser (CAMAG Derivatiser). The derivatised plates were heated for 10 min at 115 °C using a CAMAG TLC Plate Heater III. The plates were then cooled to room temperature and analysed with the HPTLC imaging device under white light [43,44]. The chromatographic images were digitally processed and analysed using specialised HPTLC software (visionCATS, CAMAG), which was also used to control the individual instrumentation modules.

#### 3.4.4. 5-Hydroxymethylfurfural (HMF) Analysis

The chromatographic separation was performed as previously described [43] at ambient temperature on silica gel 60 F_254_ HPTLC plates (glass plates 20 × 10 cm). The standard solutions were applied as 8 mm bands at 8 mm from the lower edge of the HPTLC plate at a rate of 50 nLs^−1^ using a semi-automated HPTLC application device (Linomat 5, CAMAG). To prepare the HMF standard curve, 1 µL, 2 µL, 3 µL, 4 µL and 5 µL of the respective standard solution were applied. For the analysis of the HMF in the honey samples, 10 µL of the respective sample solution was analysed.

The following automated development chamber (ADC2, CAMAG) settings were used: a pre-drying time of 1 min, humidity control (33% relative humidity) and drying time of 5 min. The plates were automatically developed to a distance of 50 mm at ambient temperature using ethyl acetate as a mobile phase. The obtained chromatographic results were documented using a TLC Scanner 4 (CAMAG) at 290 nm [49]. The chromatographic results were analysed using specialised HPTLC software (visionCATS, CAMAG), which was also used to control the individual instrumentation modules.

### 3.5. Statistical Analysis

All the quantitative experiments (major sugar and HMF content) were performed in triplicate, and the results were evaluated by a one-way analysis of variance (ANOVA) followed by Tukey’s honestly significant difference (TukeyHSD) test, where a *p*-value of less than 0.05 was considered statistically significant. All the statistical analyses were performed using Microsoft Office 365, R and R studio [55,56].

## 4. Conclusions

To our knowledge, this was the first time that some key chemical parameters (non-sugar constituent profile, sugar composition and HMF content) as well as the antioxidant activities of a range of honeys stored under different temperature conditions (ambient, 40 °C, 60 °C and 80 °C) were tracked over a five-month period. The organic extract composition of all the honeys, including the artificial honey, was stable during storage at 40 °C for up to one month, as evidenced by the consistent band patterns in the respective HPTLC fingerprints and the absence of any heat-induced artefacts. At 60 °C storage, the organic extracts of all the honeys remained stable during the short-term (48 h) period but demonstrated changes when stored for one month or longer. These changes included the appearance of a new band (Rf 0.32) in all the honeys, including the artificial honey, and the decrease in the intensity of some bands in the LEP, MAR and PEP honey beyond the 5-month storage time. At the 80 °C storage temperature, the organic extracts of all the honeys were stable for only 6 h. Afterwards, the artefact band (Rf 0.32) appeared before the honeys were completely caramelised by 48 h, rendering any further analysis impossible.

The sugar composition, specifically the samples’ F/G ratio, was stable at 40 °C for the entire study period (up to 5 months) for all the honeys except PEP, which started to change after five months. At 60 °C, the F/G ratio of all the honeys remained stable for two months and then started to significantly change over time. Interestingly, the observed changes varied between different honeys. For example, the changes were significant in the MAR honey from the second month onwards, in the ART and LEP honeys after three months and in the PEP honey after five months of storage. The samples kept at 80 °C could only be analysed for up to 48 h, and during that time, no significant changes in the sugar compositions of all the analysed honeys, including the artificial honey, were noted.

This study showed that the HPTLC-DPPH total band activities of the three honeys increased over time at higher temperatures. However, from this experiment, it is impossible to conclude the potential impact of the increased activity on human health due to the unidentified nature of the compounds responsible for the antioxidant activities. In this study, only one sample of each honey type was analysed. More samples and samples of different types of honeys must be analysed to derive any definitive conclusive statements about the effects of temperature on the antioxidant profiles of honeys.

In addition to revealing some interesting trends of the analysed honeys that require further in-depth investigations, this study also demonstrated the usefulness of HPTLC as a simple, easy-to-perform and cost-effective method for honey quality control. By facilitating the recording of various important parameters of honey quality using a single instrument and, by extension, allowing these analyses to be carried out in a single lab, the versatility of HPTLC offers great potential for the honey industry in terms of routine quality control.

## Figures and Tables

**Table 1:** Baseline HPTLC fingerprints of honeys (key band positions at specific Rf and colour).

ID	HPTLC Fingerprint Bands (Rf)							
	After Development				After Derivatisation			
	R 254		R 366		T White		R 366	
	Rf	Colour	Rf	Colour	Rf	Colour	Rf	Colour
ART	-	-	-	-	-	-	0.53	Faint blue
LEP	0.23		0.10	Faint yellow	0.23	Dark	0.10	
	0.33		0.23	Bright blue	0.32	Green	0.11	
	0.40		0.32	Blue	0.40	Orange	0.22	Blue
					0.41		0.31	Blue
					0.48	Blue	0.35	Blue
							0.40	
MAR	0.33		0.10		0.41	Red	0.17	
	0.42		0.32	Light blue	0.47	Orange	0.21	Beige
	0.47						0.36	Green
							0.40	Orange-brown
							0.48	Blue-green
PEP	0.22		0.10	Bright yellow	0.39	Blue	0.10	
	0.32		0.32	Bright blue	0.49	Orange	0.32	Bright blue
	0.38						0.39	Brick red
	0.51						0.49	Bright red

**Table 2:** Effect of temperature on the HPTLC fingerprints of the honeys.

ID	Bands	Temperature				
		At 40 °C		At 60 °C		At 80 °C
		0–48 h	48 h–5 Months	0–48 h	48 h–5 Months	0–48 h
ART	Baseline	-	-	↓ Intensity over time	↓ Intensity over time	↓ Intensity over time
	New band *		Appeared at 2 months ↑ intensity over time		Appeared at 1 month ↑↑ intensity over time	Appeared at 6 h ↑↑ intensity over time
LEP	Baseline	-	↓ intensity over time	-	↓↓ intensity over time	↓ intensity over time
	New band *		Appeared at 1 month ↑ intensity over time		Appeared at 1 month ↑↑ intensity over time	Appeared at 12 h ↑↑ intensity over time
MAR	Baseline	-	↓ intensity over time	-	↓↓ intensity over time	↓ intensity over time
	New band *		Appeared at 1 month ↑ intensity over time		Appeared at 1 month ↑↑ intensity over time	Appeared at 12 h ↑↑ intensity over time
PEP	Baseline	-	↓ intensity over time	-	↓↓ intensity over time	↓ intensity over time
	New band *		Appeared at 2 months ↑ intensity over time		Appeared at 1 month ↑↑ intensity over time	Appeared at 24 h ↑ intensity over time

* New band formation at Rf 0.32.

**Table 3:** Fructose and glucose content in different honeys.

Honey	Fructose (mg per g Honey)	Glucose (mg per g Honey)	F/G
	Average ± SD	Average ± SD	
ART	395.58 ± 7.46	325.12 ± 20.06	1.22
LEP	400.89 ± 9.74	274.63 ± 4.81	1.46
MAR	423.68 ± 6.05	236.61 ± 5.71	1.79
PEP	404.31 ± 12.11	247.94 ± 9.25	1.63

**Table 4:** Change in the fructose to glucose ratio over time compared to baseline (0 min).

Honey	Term	Temperature	Time Point (*p*-Value)
ART	Short	40 °C	No significant difference
		60 °C	No significant difference
		80 °C	No significant difference
	Long	40 °C	No significant difference
		60 °C	3 months (*p* = 0.00292), 4 months (*p* = 0.03763) and 5 months (*p* = 0.0397)
LEP	Short	40 °C	No significant difference
		60 °C	No significant difference
		80 °C	No significant difference
	Long	40 °C	No significant difference
		60 °C	3 months (*p* = 0.01994), 4 months (*p* = 0.000003) and 5 months (*p* = 0.0000005)
MAR	Short	40 °C	No significant difference
		60 °C	No significant difference
		80 °C	No significant difference
	Long	40 °C	No significant difference
		60 °C	2 months (*p* = 0.0005369), 3 months (*p* = 0.00000), 4 months (*p* = 0.0000024) and 5 months (*p* = 0.00000)
PEP	Short	40 °C	No significant difference
		60 °C	No significant difference
		80 °C	No significant difference
	Long	40 °C	5 months (*p* = 0.00367)
		60 °C	5 months (*p* = 0.00133)

**Table 5:** 5-Hydroxymethylfurfural (HMF) content in different honeys.

Honey	HMF (mg/kg)	SD
ART	–	–
LEP	189.51	5.43
MAR	36.75	6.34
PEP	–	–

**Table 6:** Honey samples, including packaging information and sample ID.

Honey Type	Floral Source	Sample ID
Artificial	N/A	ART
Manuka	*Leptospermum* sp.	LEP
Marri/WA Red Gum	*Corymbia calophylla*	MAR
Coastal Peppermint	*Agonis flexuosa*	PEP

## Data Availability

All data are presented either in the manuscript or in the Supplementary Material.

## Supplementary Materials

The following supporting information can be downloaded at: www.mdpi.com/article/10.3390/molecules27238491/s1.
