# Supplementary material for: A Comprehensive HPTLC-Based Analysis of the Impacts of Temperature on the Chemical Properties and Antioxidant Activity of Honey
Source: Molecules. 2022 Dec 2;27(23):8491. doi: 10.3390/molecules27238491 (PMC9737681; doi:10.3390/molecules27238491)
Supplement: Supplementary file 1 [file molecules-27-08491-s001.zip › molecules-2044053-supplementary.pdf]

Supplementary Material

A Comprehensive HPTLC-Based Analysis of the Impacts of Temperature on the Chemical Properties and Antioxidant Activity of Honey

Md Khairul Islam [1,2], Tomislav Sostaric [2], Lee Yong Lim [2], Katherine Hammer [1,3] and Cornelia Locher [1,2,*]

[1] Cooperative Research Centre for Honey Bee Products Limited (CRC HBP), University of Western Australia, Perth 6009, Australia;
[2] Division of Pharmacy, School of Allied Health, University of Western Australia, Perth 6009, Australia
[3] School of Biomedical Sciences, University of Western Australia, Perth 6009, Australia
* Correspondence: connie.locher@uwa.edu.au

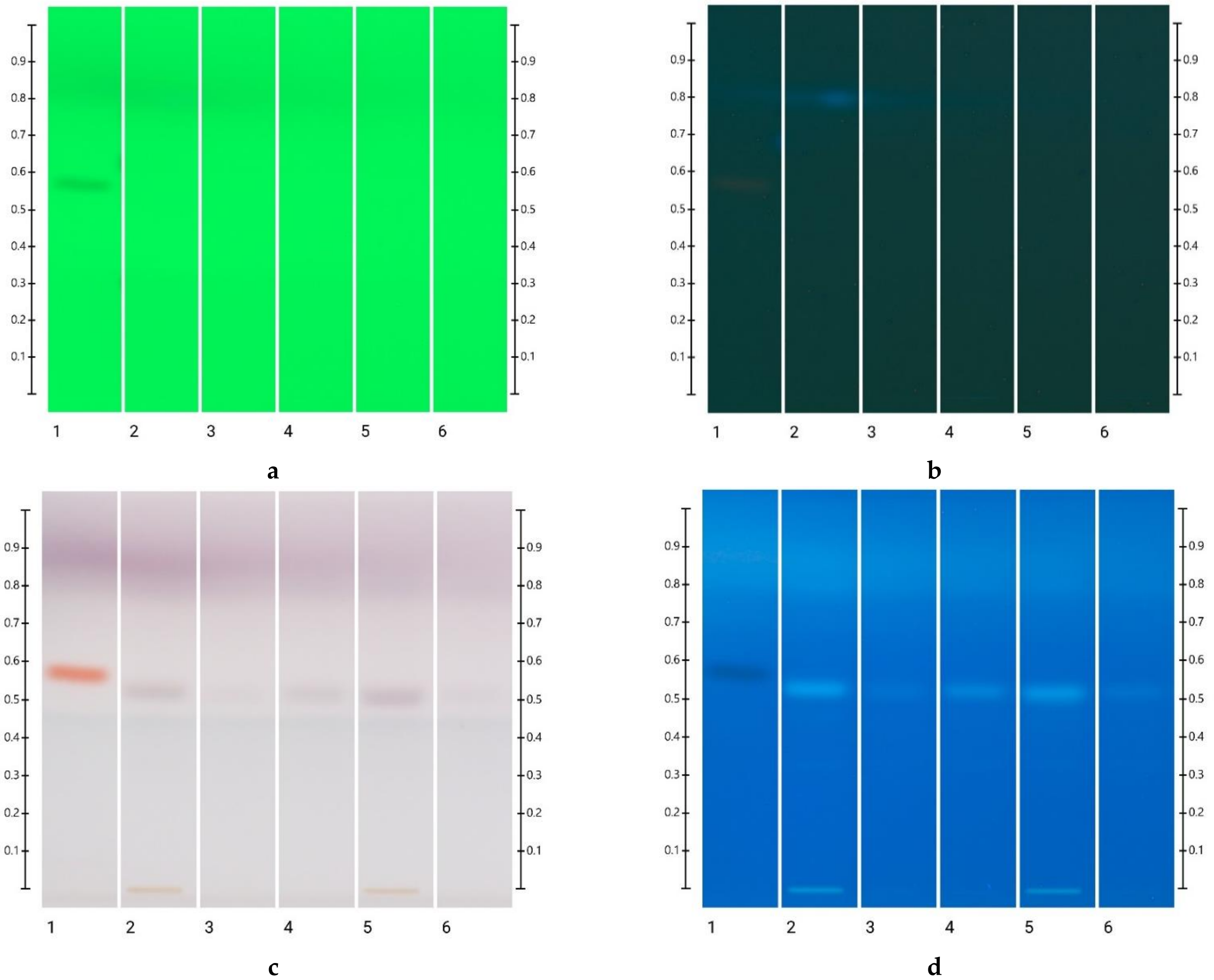

Supplementary Figure S1. ART short-term storage at 40 °C; images taken at (**a**) 254 nm; (**b**) 366 nm; (**c**) White light after derivatisation and (**d**) 366 nm after derivatisation with vanillin reagent; Track 1—4,5,7-trihydroxyflavanon, Track 2—0 h, Track 3—6 h, Track 4—12 h, Track 5—24 h, and Track 6—48 h; 5 μL of each honey extract respectively.

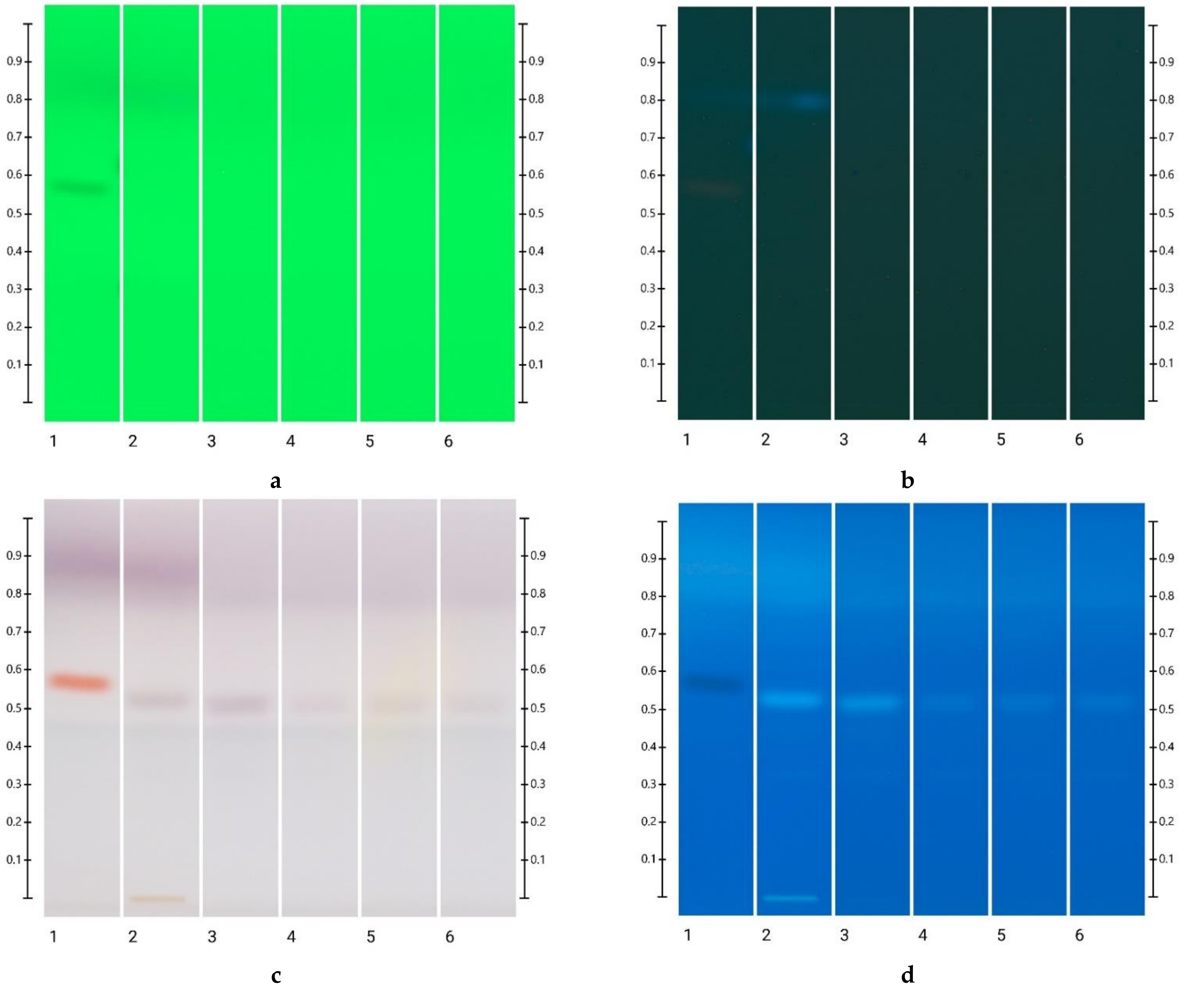

Supplementary Figure S2. ART short-term storage at 60 °C; images taken at (**a**) 254 nm; (**b**) 366 nm; (**c**) White light after derivatisation and (**d**) 366 nm after derivatisation with vanillin reagent; Track 1— 4,5,7-trihydroxyflavanon, Track 2— 0 h, Track 3— 6 h, Track 4— 12 h, Track 5— 24 h, and Track 6— 48 h; 5 µL of each honey extract respectively.

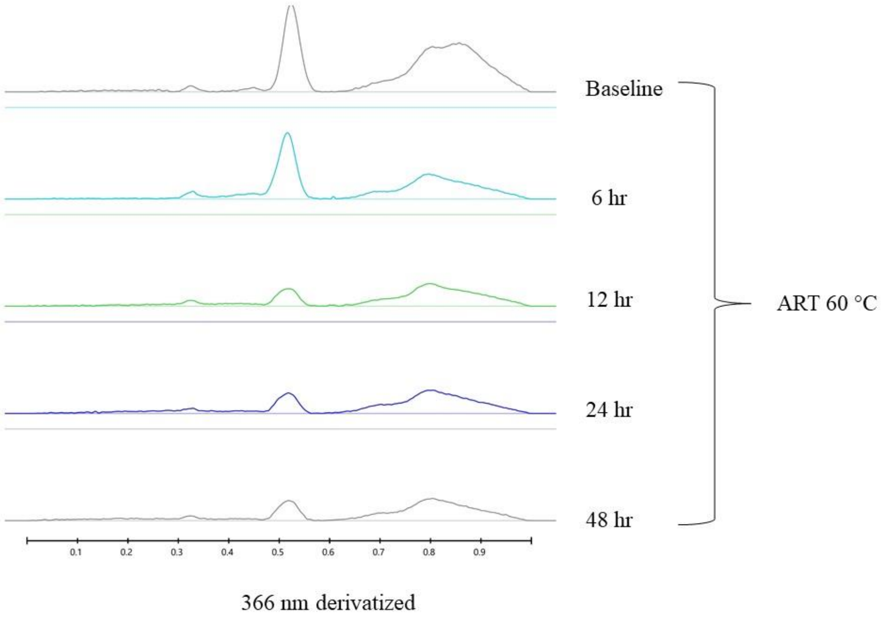

Supplementary Figure S3. Organic honey extract chromatograms (offset Y-axis values).

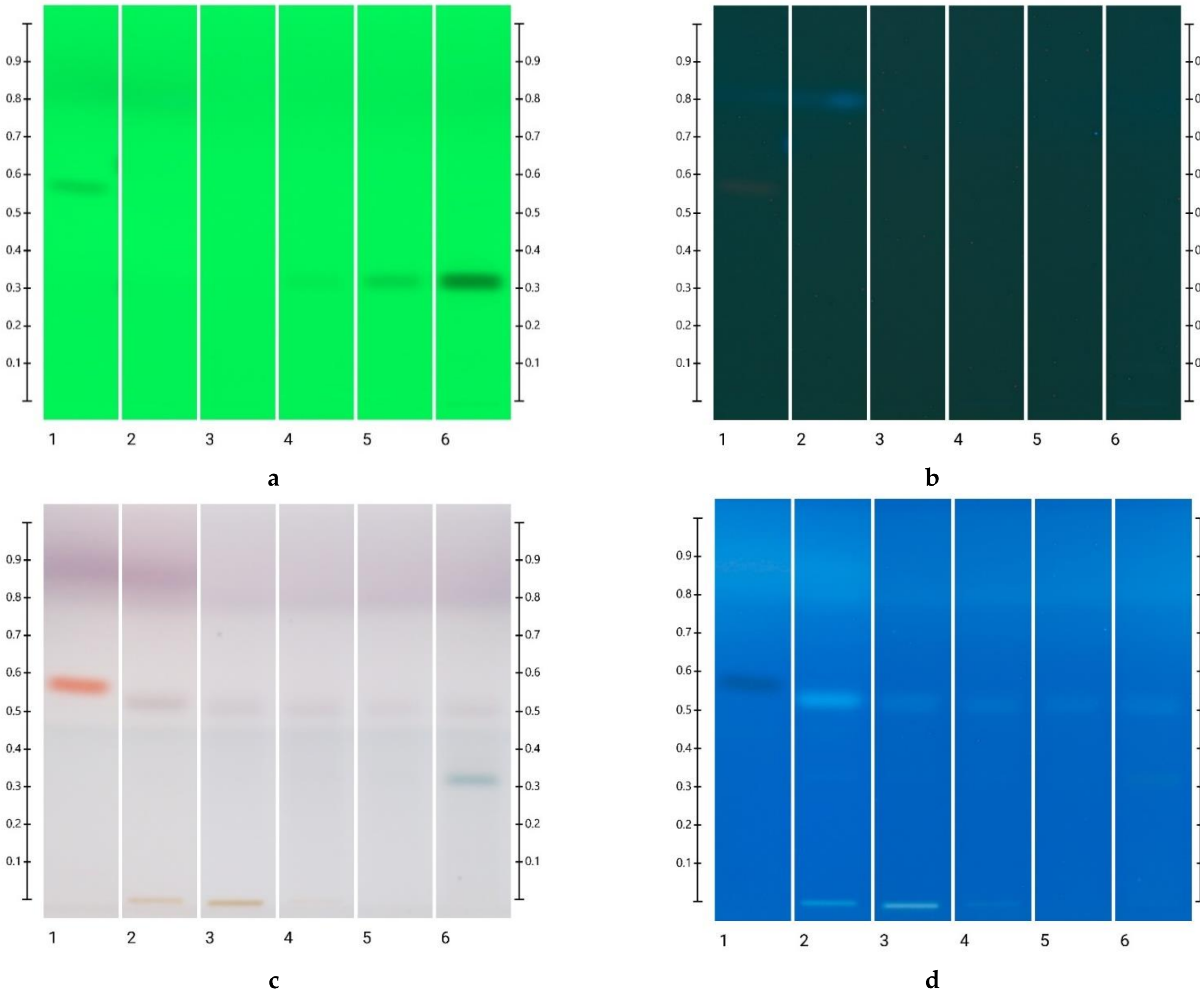

Supplementary Figure S4. ART short-term storage at 80 °C; images taken at (**a**) 254 nm; (**b**) 366 nm; (**c**) White light after derivatisation and (**d**) 366 nm after derivatisation with vanillin reagent; Track 1— 4,5,7-trihydroxyflavanon, Track 2— 0 h, Track 3— 6 h, Track 4— 12 h, Track 5— 24 h, and Track 6— 48 h; 5 µL of each honey extracts respectively.

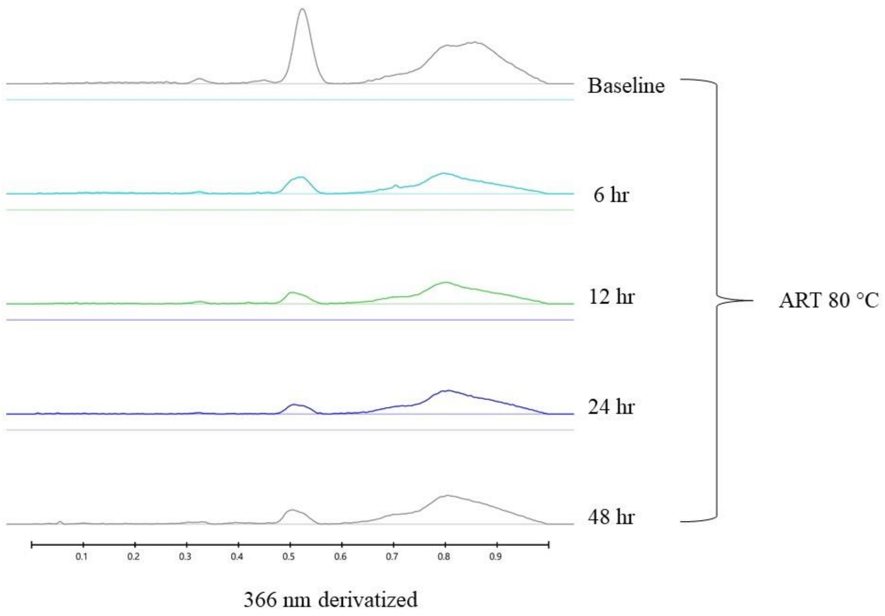

Supplementary Figure S5. Organic honey extract chromatograms (offset Y-axis values).

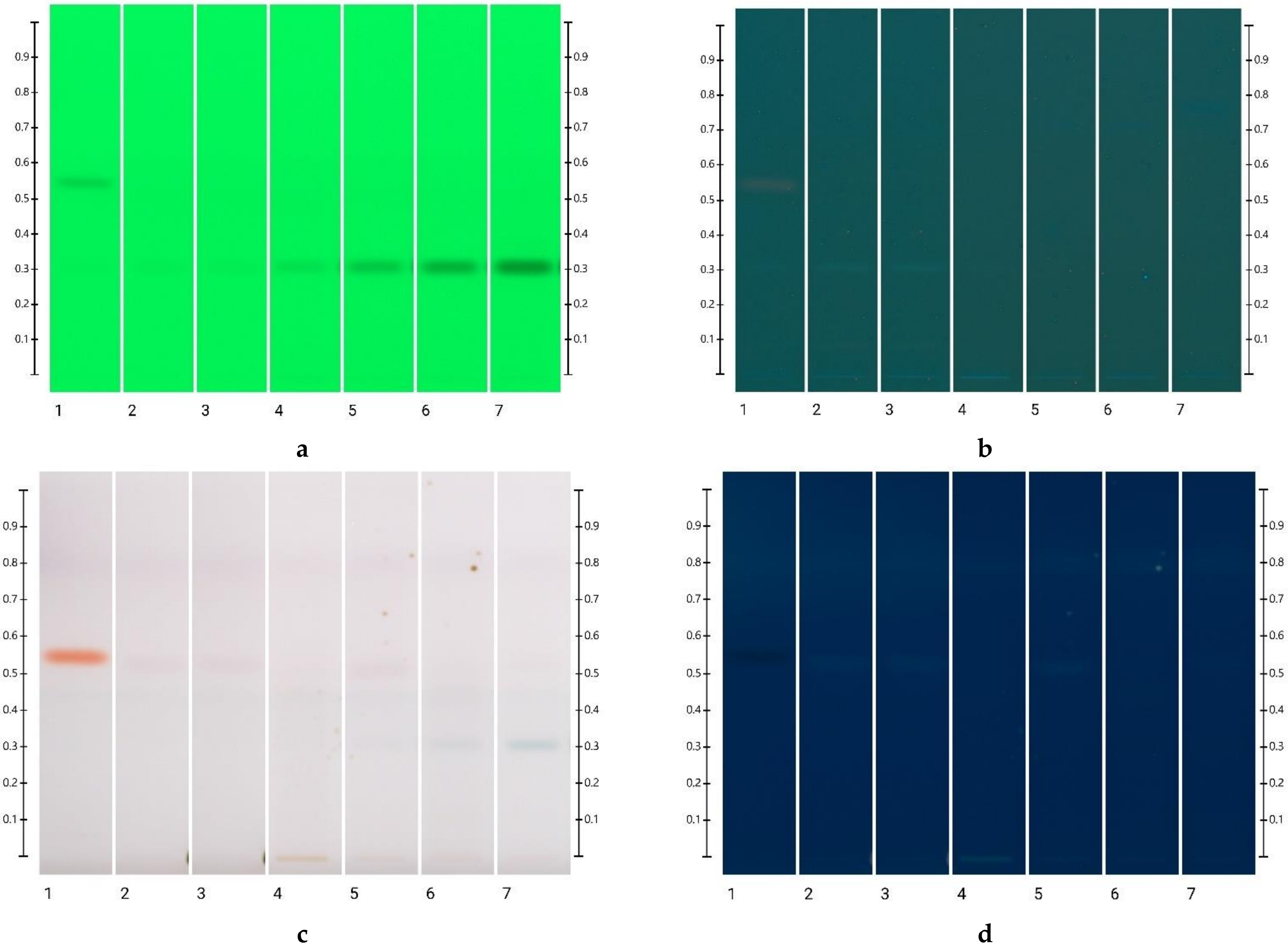

Supplementary Figure S6. ART long-term storage at 40 °C Images taken at (**a**) 254 nm; (**b**) 366 nm; (**c**) White light after derivatisation and (**d**) 366 nm after derivatisation with vanillin reagent; Track 1—4,5,7-trihydroxyflavanon, Track 2— 0 h, Track 3— 1 month, Track 4— 2 month, Track 5— 3 month, Track 6— 4 month, and Track 7— 5 month; 5 μL of each honey extract respectively.

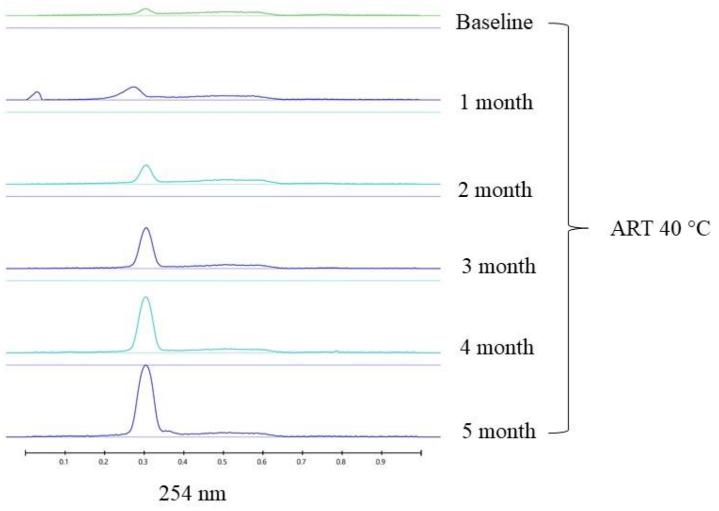

Supplementary Figure S7. Organic honey extract chromatograms.

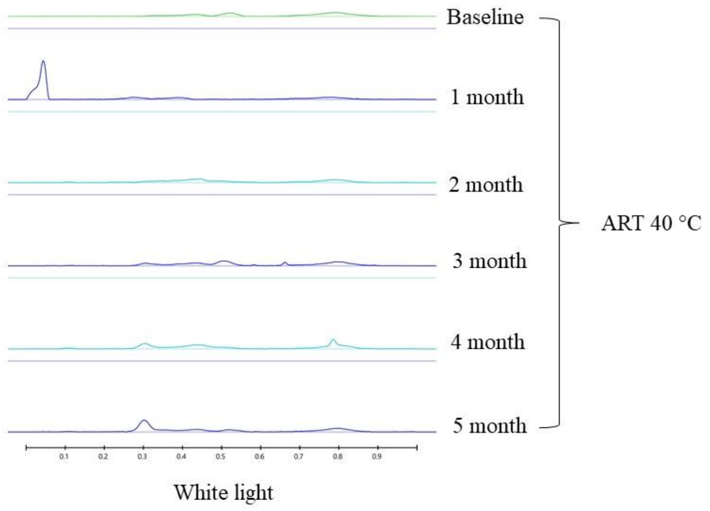

Supplementary Figure S8. Organic honey extract chromatograms.

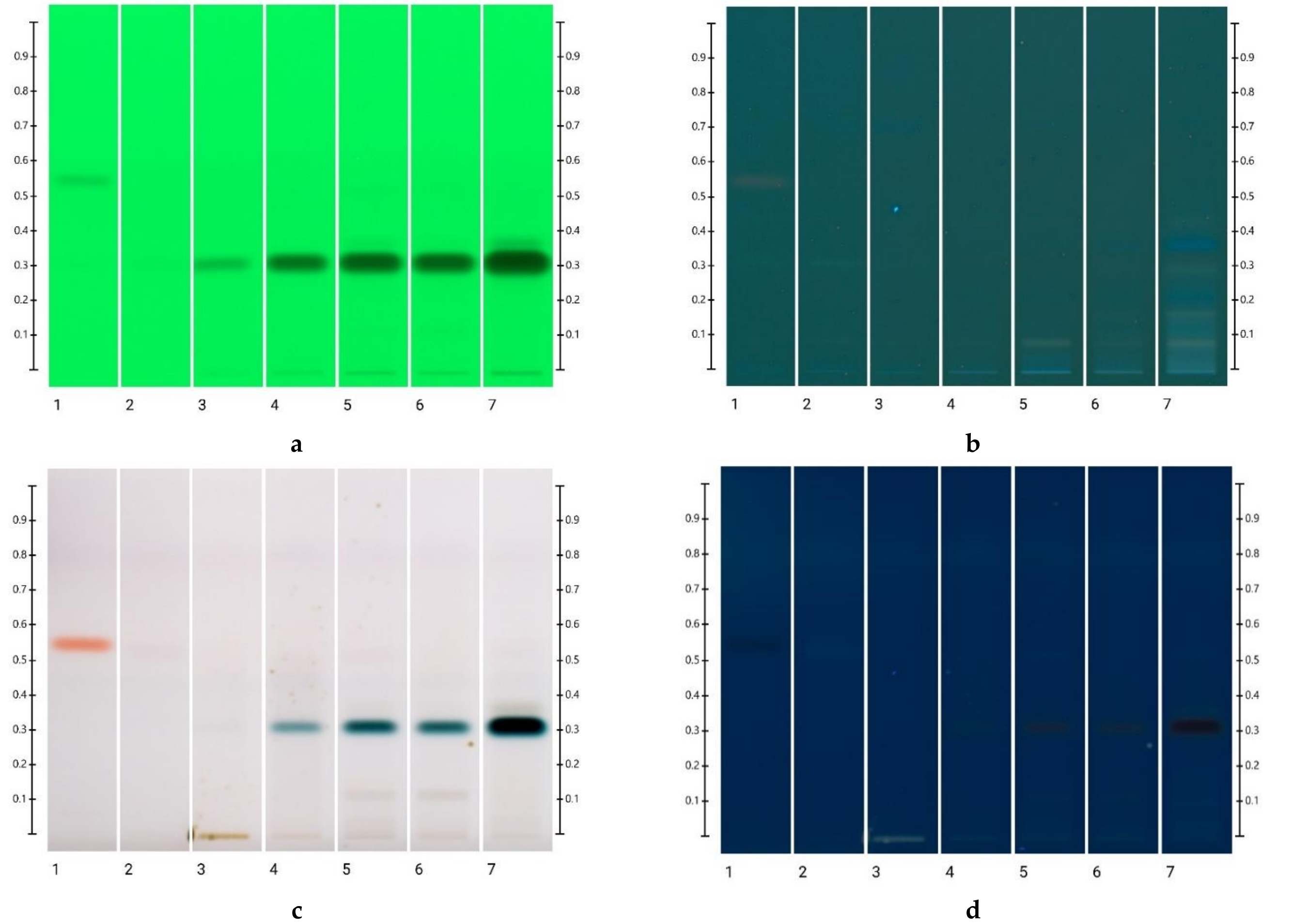

Supplementary Figure S9. ART long-term storage at 60 °C Images taken at (**a**) 254 nm; (**b**) 366 nm; (**c**) White light after derivatisation and (**d**) 366 nm after derivatisation with vanillin reagent; Track 1—4,5,7-trihydroxyflavanon, Track 2— 0 h, Track 3— 1 month, Track 4— 2 month, Track 5— 3 month, Track 6— 4 month, and Track 7— 5 month; 5 µL of each honey extract respectively.

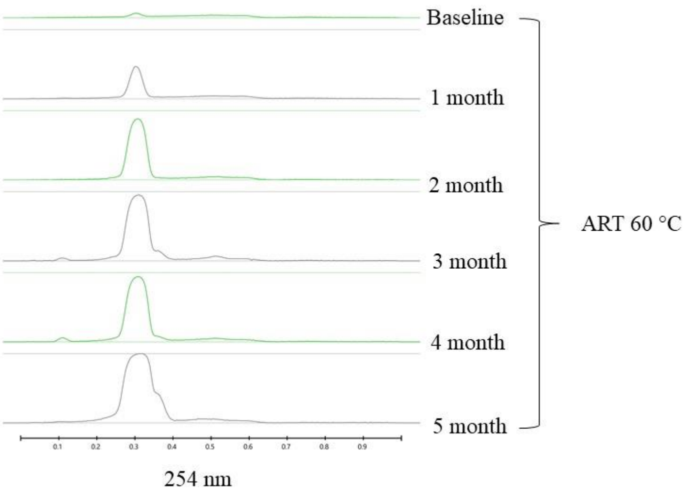

Supplementary Figure S10. Organic honey extract chromatograms (offset Y-axis values).

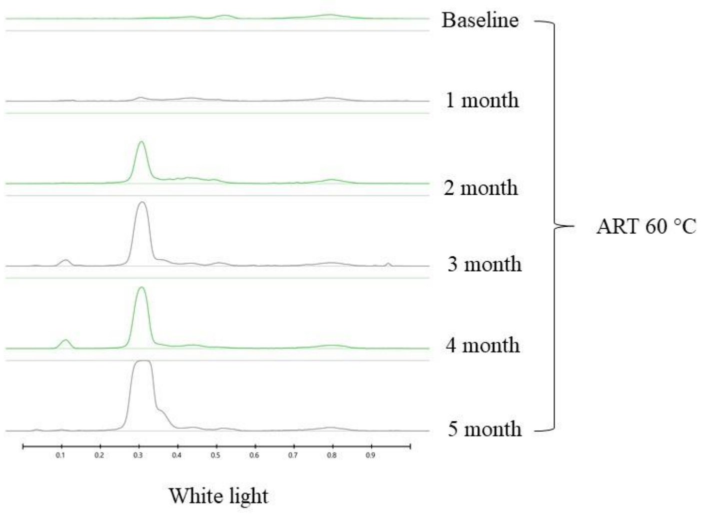

Supplementary Figure S11. Organic honey extract chromatograms (offset Y-axis values).

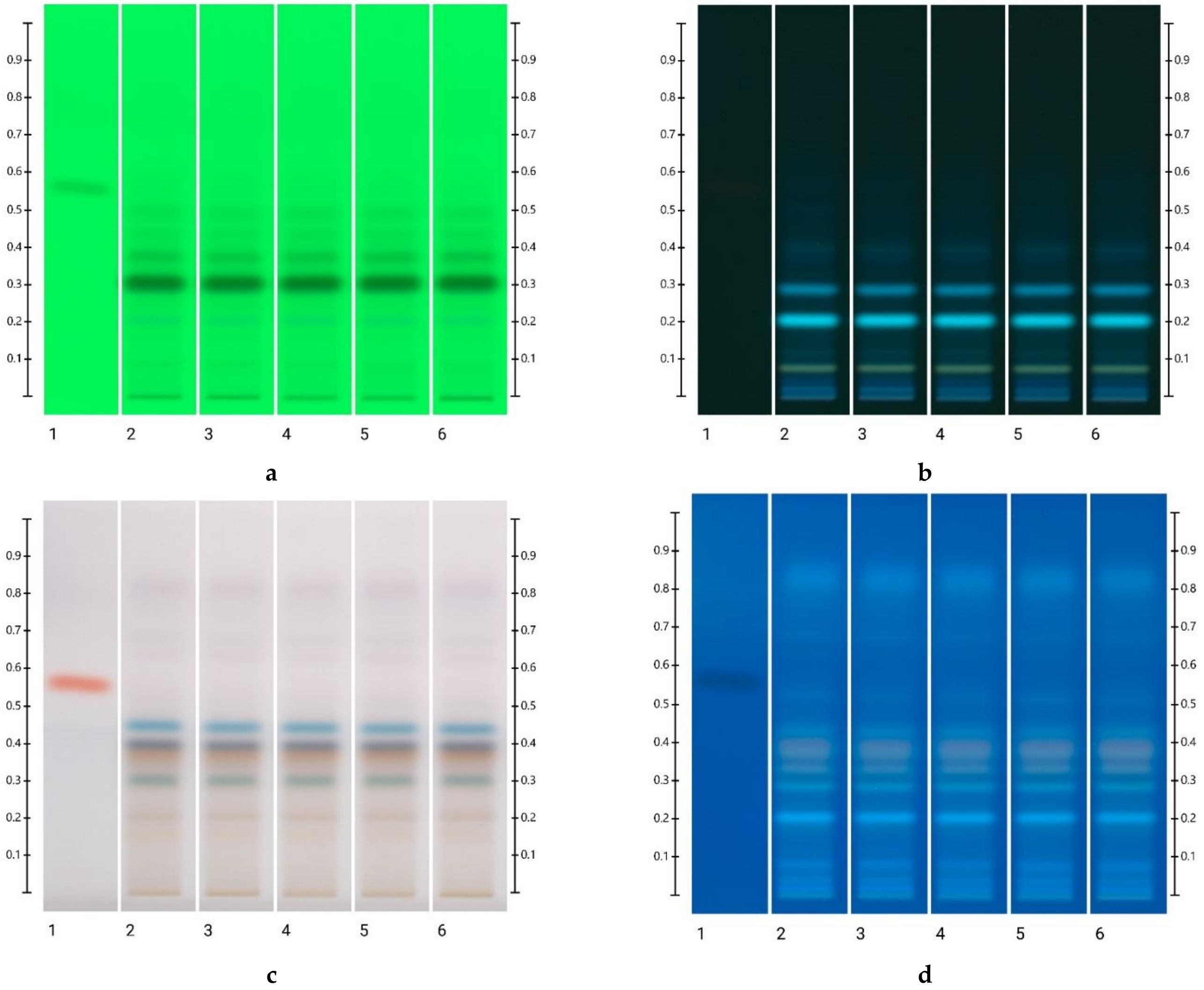

Supplementary Figure S12. LEP short-term storage at 40 °C; images taken at (**a**) 254 nm; (**b**) 366 nm; (**c**) White light after derivatisation and (**d**) 366 nm after derivatisation with vanillin reagent; Track 1— 4,5,7-trihydroxyflavanon, Track 2— 0 h, Track 3— 6 h, Track 4— 12 h, Track 5— 24 h, and Track 6— 48 h; 5 µL of each honey extract respectively.

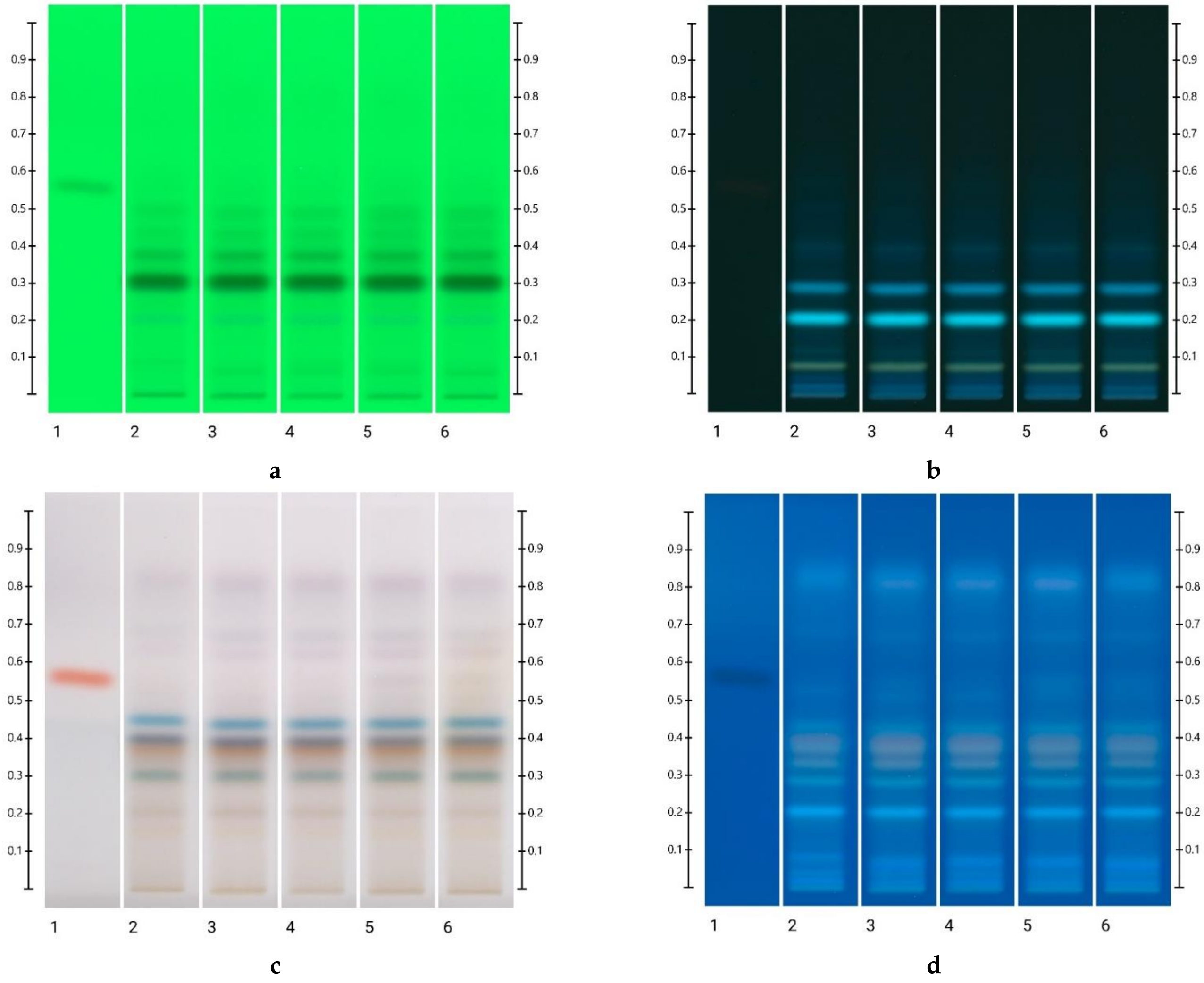

Supplementary Figure S13. LEP short-term storage at 60 °C; images taken at (**a**) 254 nm; (**b**) 366 nm; (**c**) White light after derivatisation and (**d**) 366 nm after derivatisation with vanillin reagent; Track 1— 4,5,7-trihydroxyflavanon, Track 2— 0 h, Track 3— 6 h, Track 4— 12 h, Track 5— 24 h, and Track 6— 48 h; 5 µL of each honey extract respectively.

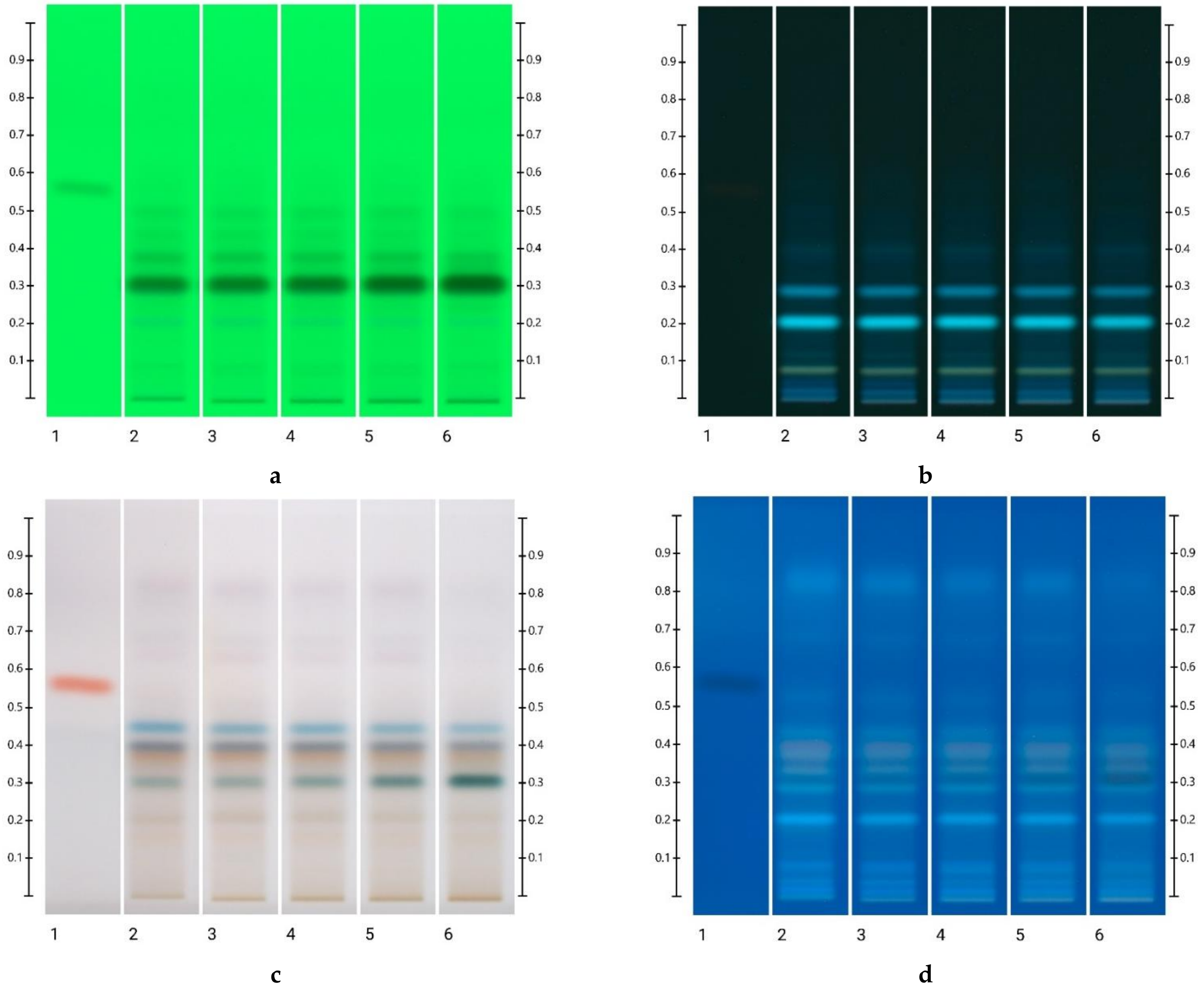

Supplementary Figure S14. LEP short-term storage at 80 °C; images taken at (**a**) 254 nm; (**b**) 366 nm; (**c**) White light after derivatisation and (**d**) 366 nm after derivatisation with vanillin reagent; Track 1— 4,5,7-trihydroxyflavanon, Track 2— 0 h, Track 3— 6 h, Track 4— 12 h, Track 5— 24 h, and Track 6— 48 h; 5 µL of each honey extract respectively.

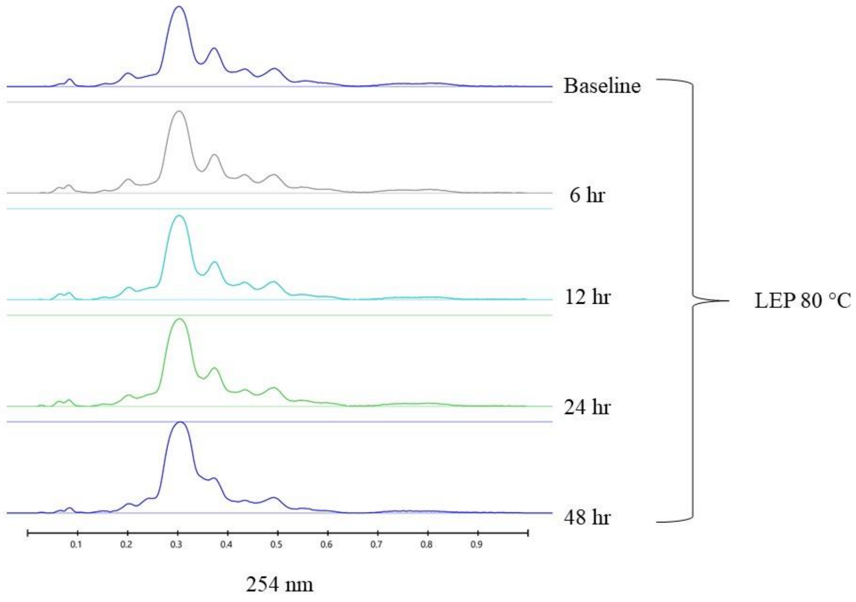

Supplementary Figure S15. Organic honey extract chromatograms (offset Y-axis values).

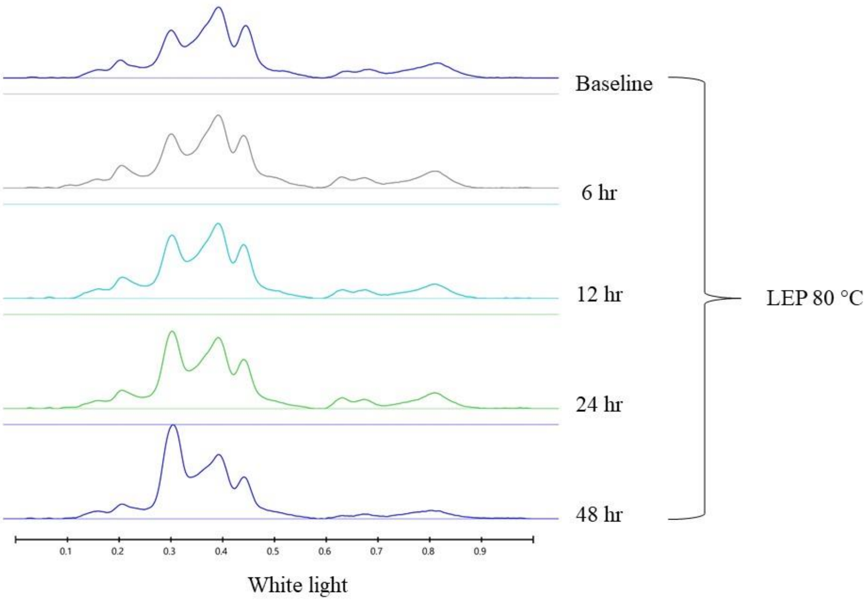

Supplementary Figure S16. Organic honey extract chromatograms (offset Y-axis values).

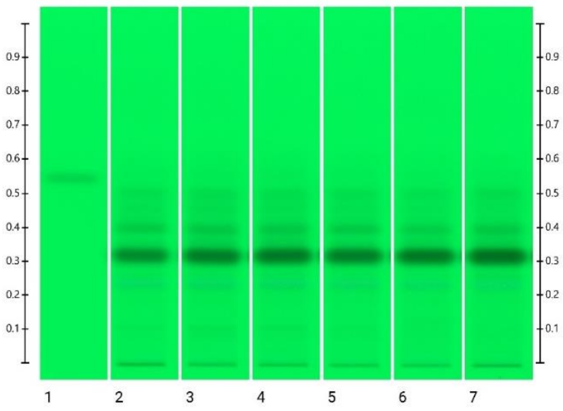

a

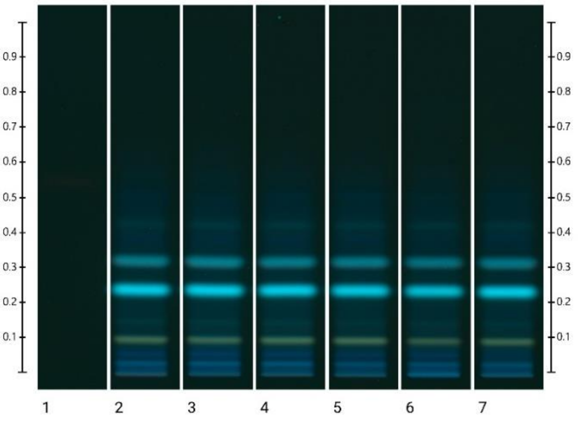

b

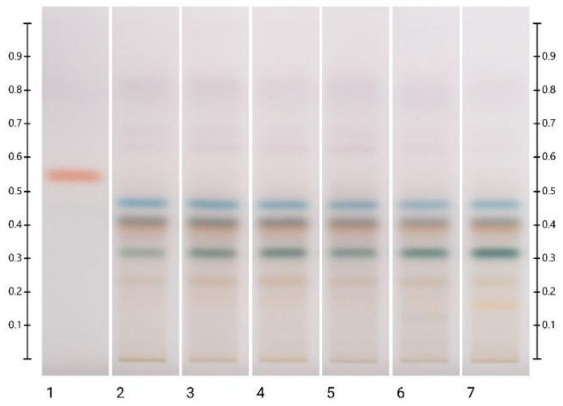

c

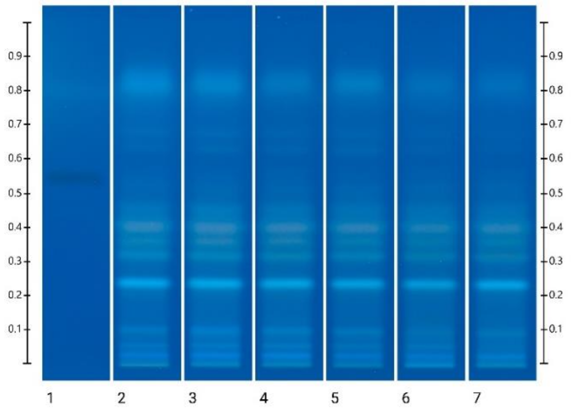

d

Supplementary Figure S17. LEP long-term storage at 40 °C Images taken at (**a**) 254 nm; (**b**) 366 nm; (**c**) White light after derivatisation and (**d**) 366 nm after derivatisation with vanillin reagent; Track 1—4,5,7-trihydroxyflavanon, Track 2— 0 h, Track 3— 1 month, Track 4— 2 month, Track 5— 3 month, Track 6— 4 month, and Track 7— 5 month; 5 µL of each honey extract respectively.

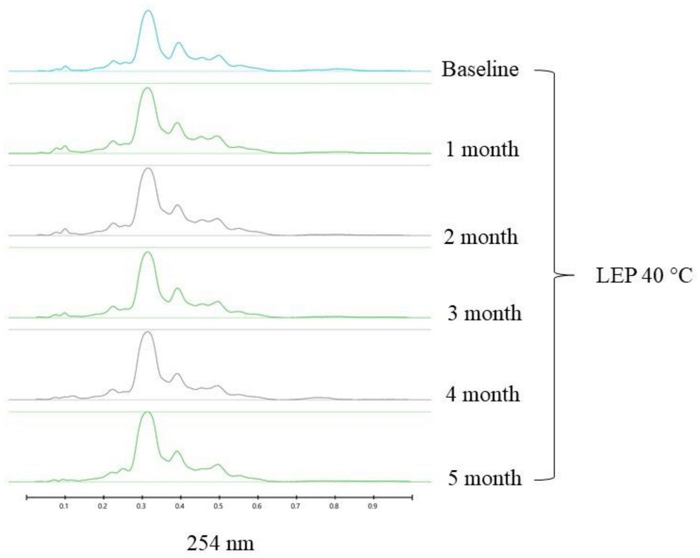

Supplementary Figure S18. Organic honey extract chromatograms (offset Y-axis values).

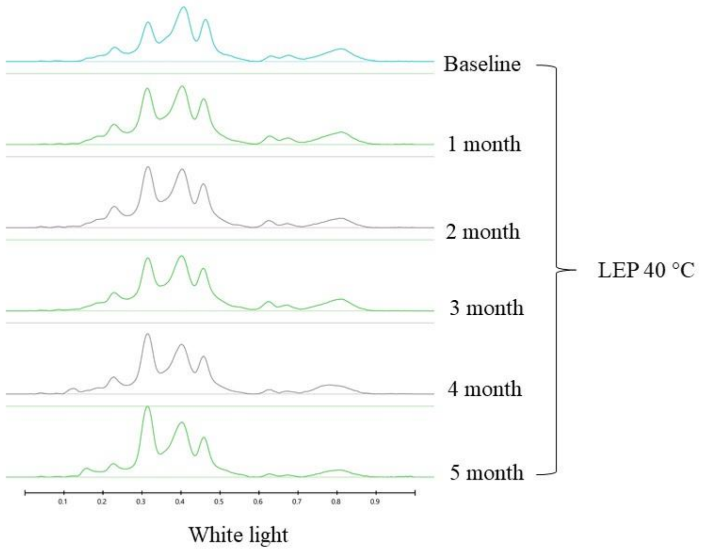

Supplementary Figure S19. Organic honey extract chromatograms (offset Y-axis values).

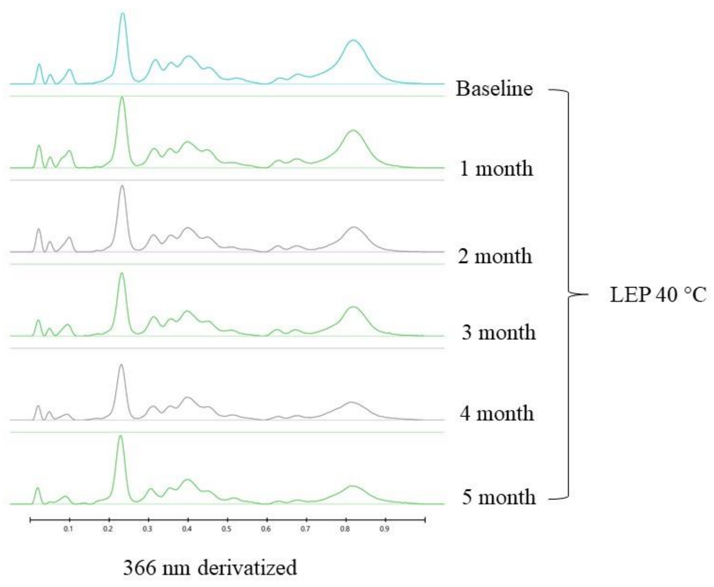

Supplementary Figure S20. Organic honey extract chromatograms (offset Y-axis values).

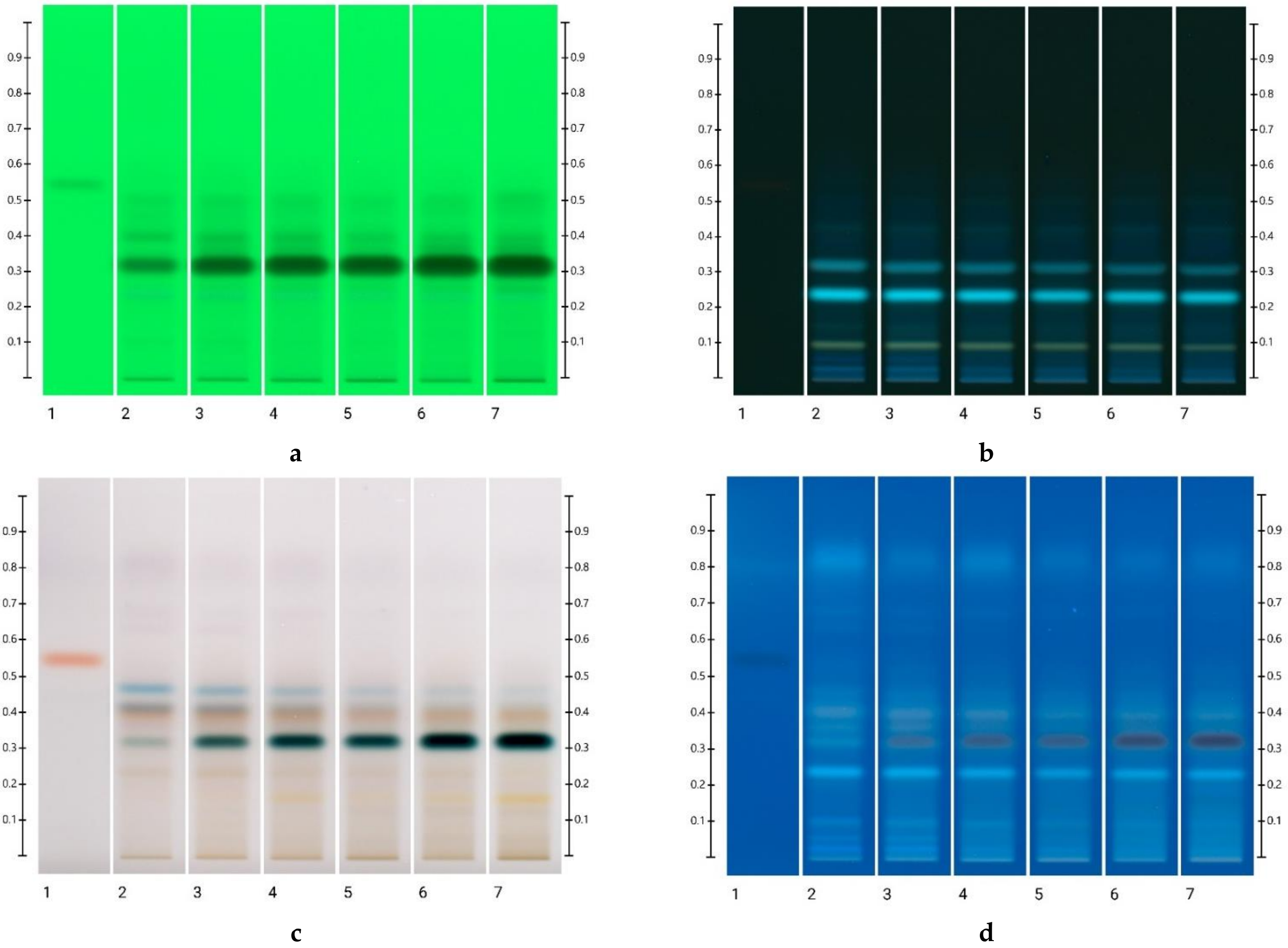

Supplementary Figure S21. LEP long-term storage at 60 °C Images taken at (**a**) 254 nm; (**b**) 366 nm; (**c**) White light after derivatisation and (**d**) 366 nm after derivatisation with vanillin reagent; Track 1—4,5,7-trihydroxyflavanon, Track 2— 0 h, Track 3— 1 month, Track 4— 2 month, Track 5— 3 month, Track 6— 4 month, and Track 7— 5 month; 5 µL of each honey extract respectively.

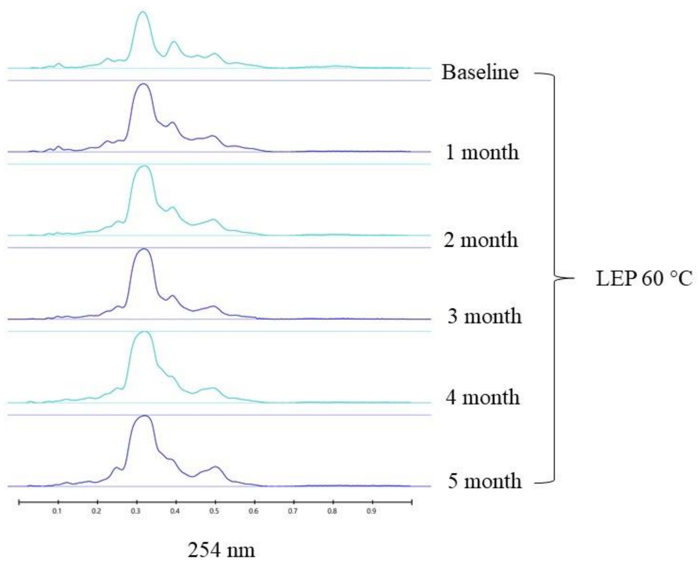

Supplementary Figure S22. Organic honey extract chromatograms (offset Y-axis values).

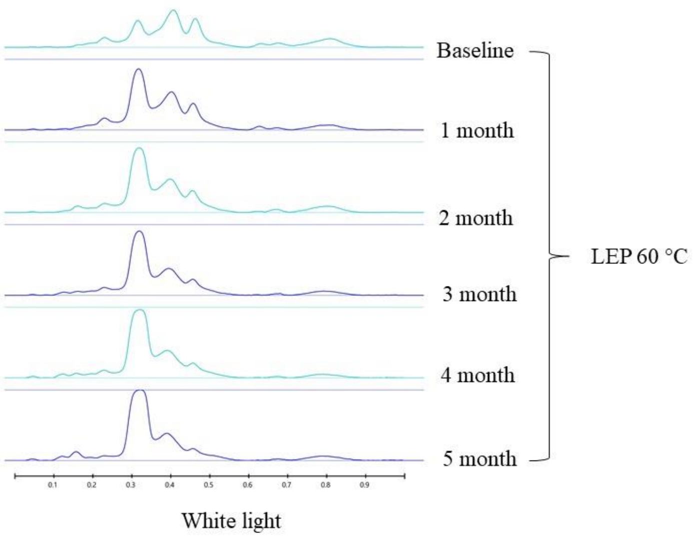

Supplementary Figure S23. Organic honey extract chromatograms (offset Y-axis values).

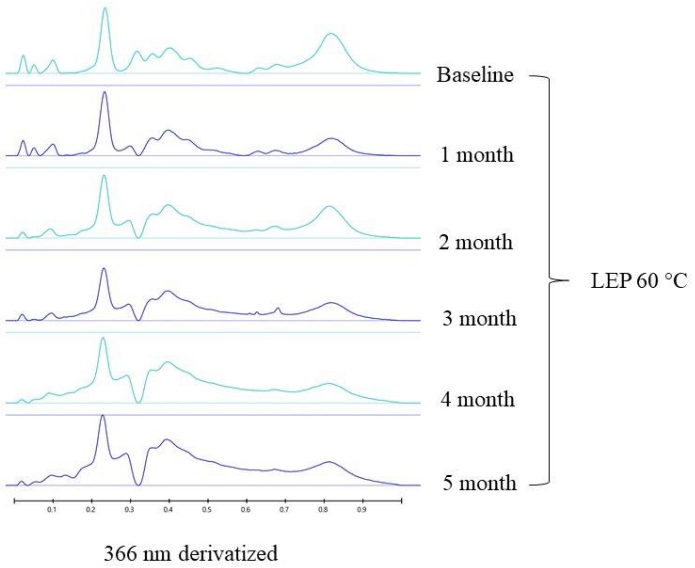

Supplementary Figure S24. Organic honey extract chromatograms (offset Y-axis values).

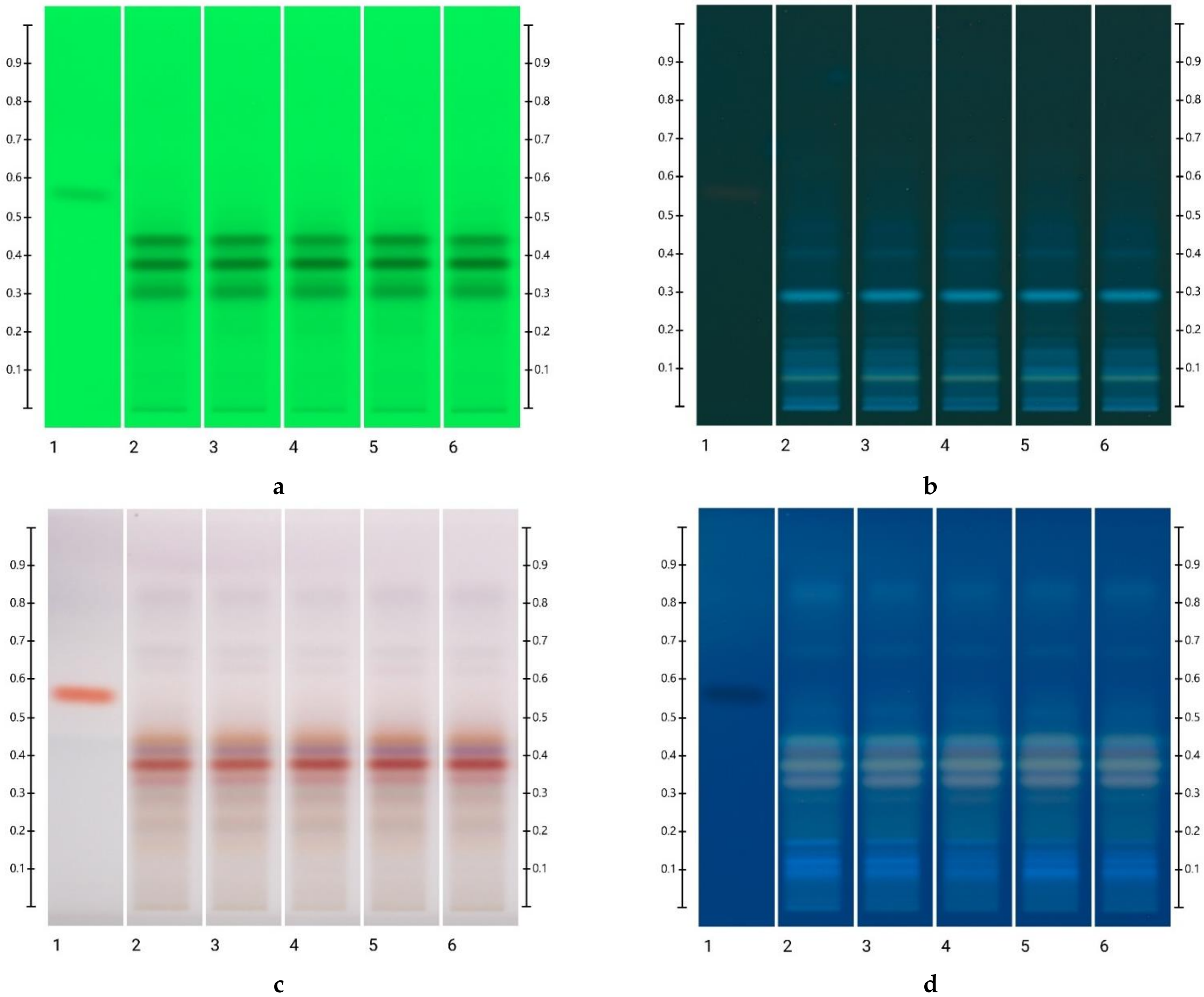

Supplementary Figure S25. MAR short-term storage at 40 °C; images taken at (**a**) 254 nm; (**b**) 366 nm; (**c**) White light after derivatisation and (**d**) 366 nm after derivatisation with vanillin reagent; Track 1— 4,5,7-trihydroxyflavanon, Track 2— 0 h, Track 3— 6 h, Track 4— 12 h, Track 5— 24 h, and Track 6— 48 h; 5 µL of each honey extract respectively.

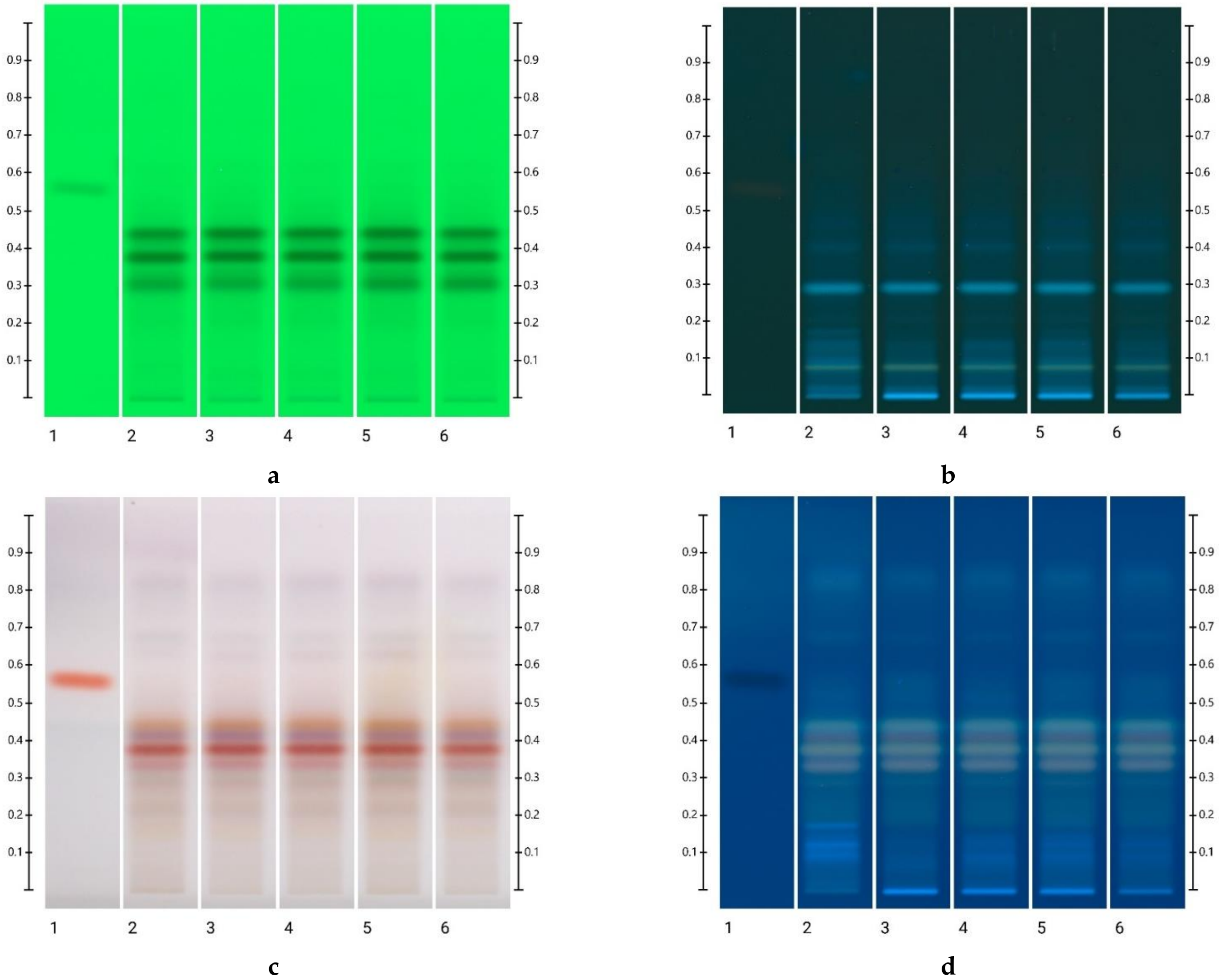

Supplementary Figure S26. MAR short-term storage at 60 °C; images taken at (**a**) 254 nm; (**b**) 366 nm; (**c**) White light after derivatisation and (**d**) 366 nm after derivatisation with vanillin reagent; Track 1— 4,5,7-trihydroxyflavanon, Track 2— 0 h, Track 3— 6 h, Track 4— 12 h, Track 5— 24 h, and Track 6— 48 h; 5 µL of each honey extract respectively.

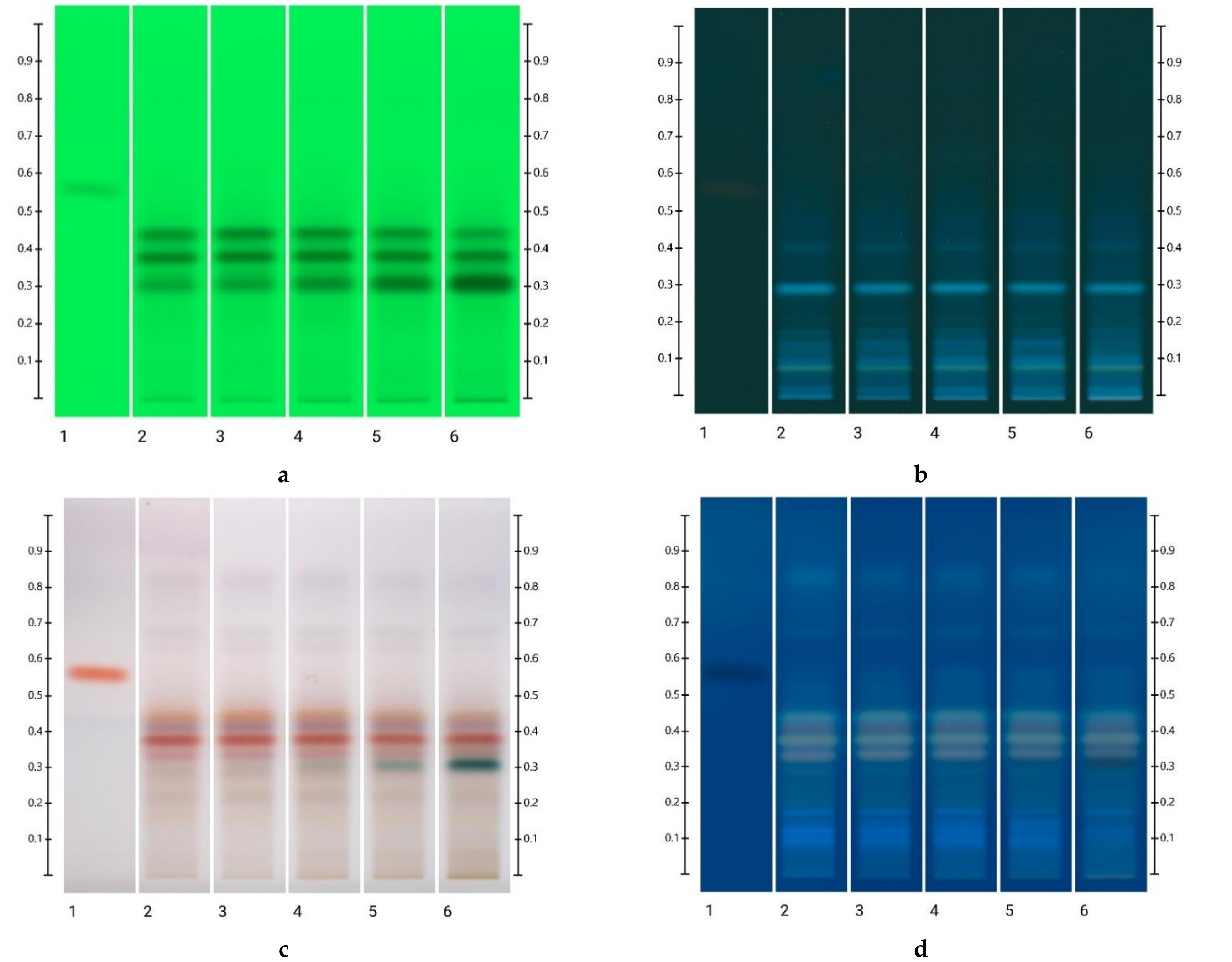

Supplementary Figure S27. MAR short-term storage at 80 °C; images taken at (**a**) 254 nm; (**b**) 366 nm; (**c**) White light after derivatisation and (**d**) 366 nm after derivatisation with vanillin reagent; Track 1— 4,5,7-trihydroxyflavanon, Track 2— 0 h, Track 3— 6 h, Track 4— 12 h, Track 5— 24 h, and Track 6— 48 h; 5 μL of each honey extract respectively.

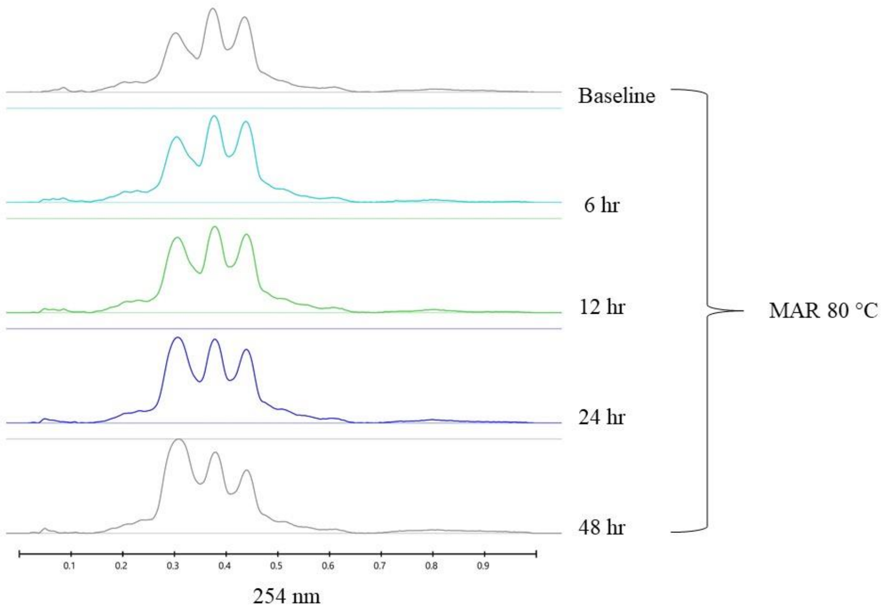

Supplementary Figure S28. Organic honey extract chromatograms (offset Y-axis values).

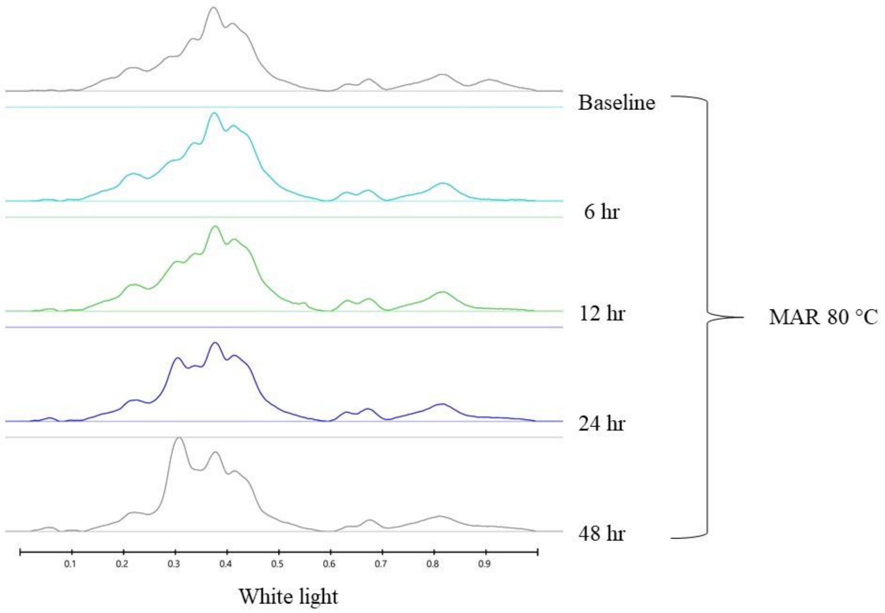

Supplementary Figure S29. Organic honey extract chromatograms (offset Y-axis values).

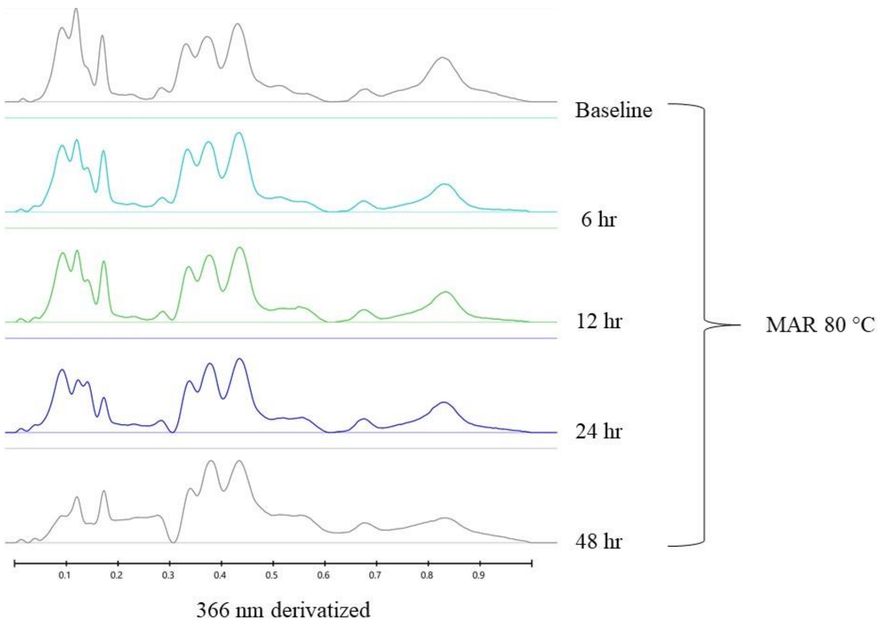

Supplementary Figure S30. Organic honey extract chromatograms (offset Y-axis values).

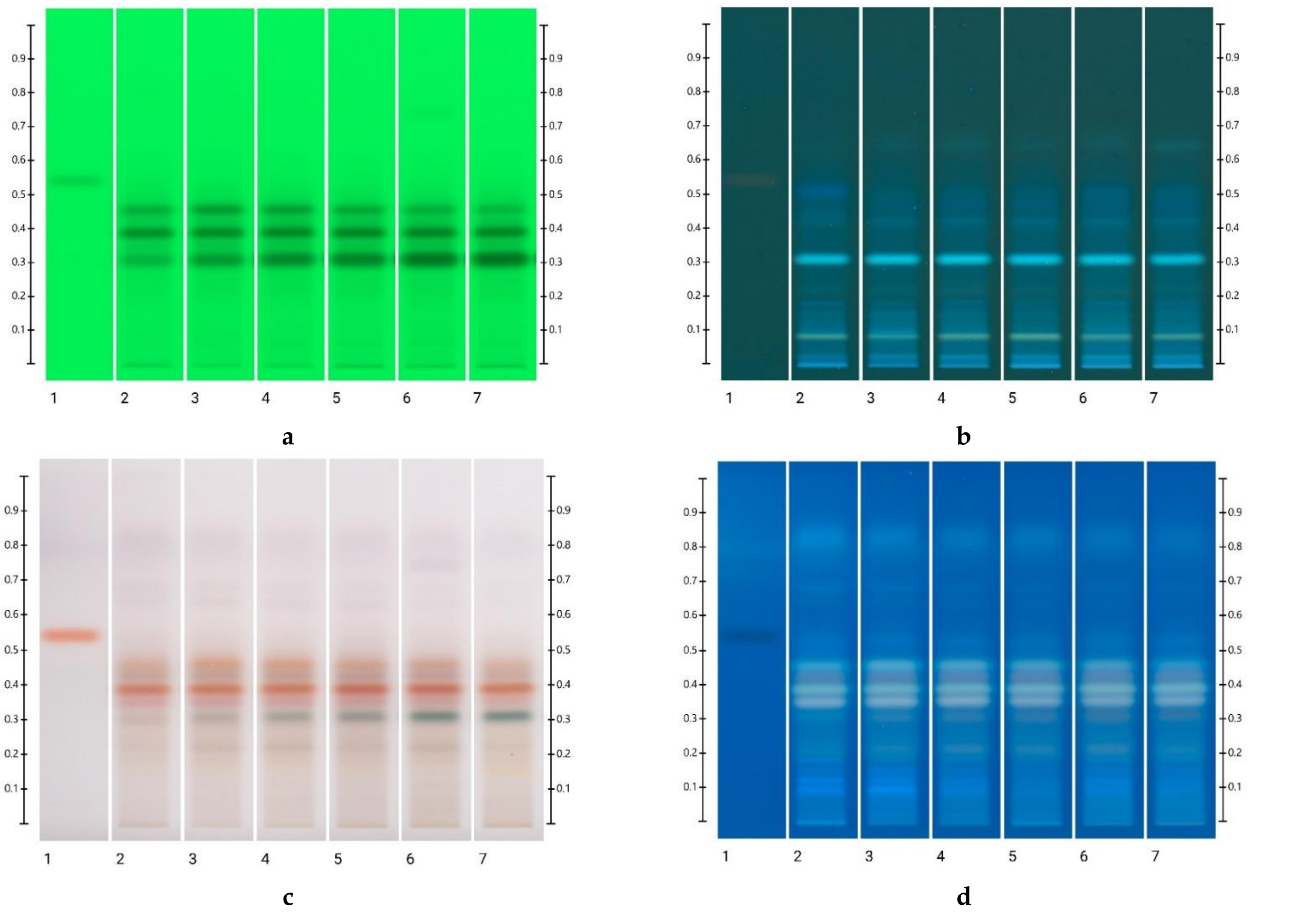

Supplementary Figure S31. MAR long-term storage at 40 °C Images taken at (**a**) 254 nm; (**b**) 366 nm; (**c**) White light after derivatisation and (**d**) 366 nm after derivatisation with vanillin reagent; Track 1—4,5,7-trihydroxyflavanon, Track 2— 0 h, Track 3— 1 month, Track 4— 2 month, Track 5— 3 month, Track 6— 4 month, and Track 7— 5 month; 5 μL of each honey extract respectively.

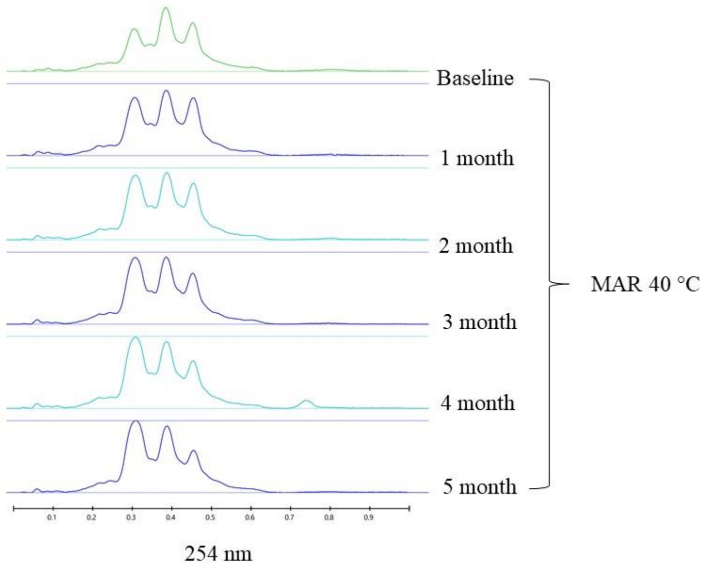

Supplementary Figure S32. Organic honey extract chromatograms (offset Y-axis values).

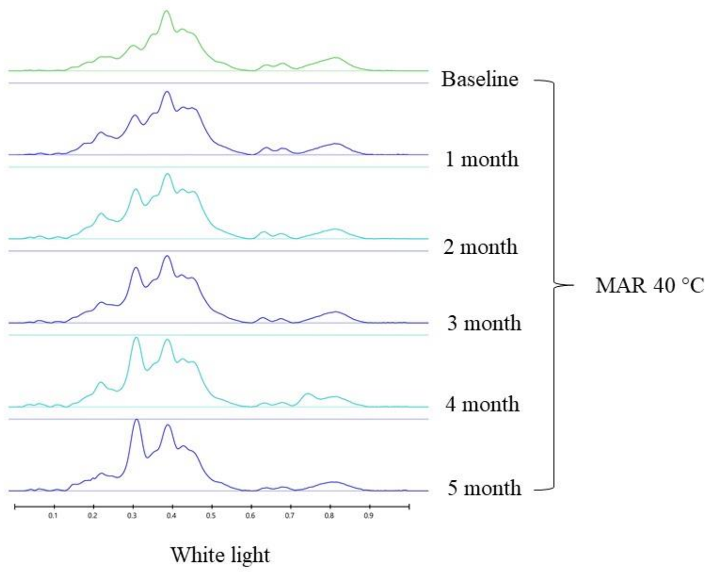

Supplementary Figure S33. Organic honey extract chromatograms (offset Y-axis values).

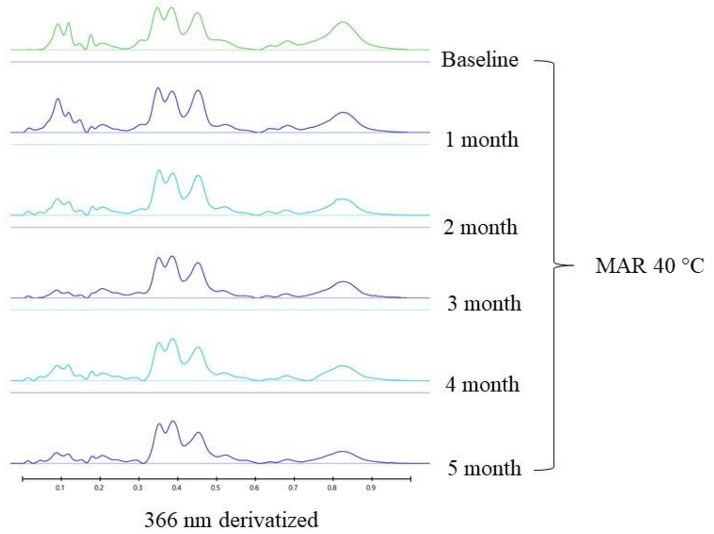

Supplementary Figure S34. Organic honey extract chromatograms (offset Y-axis values).

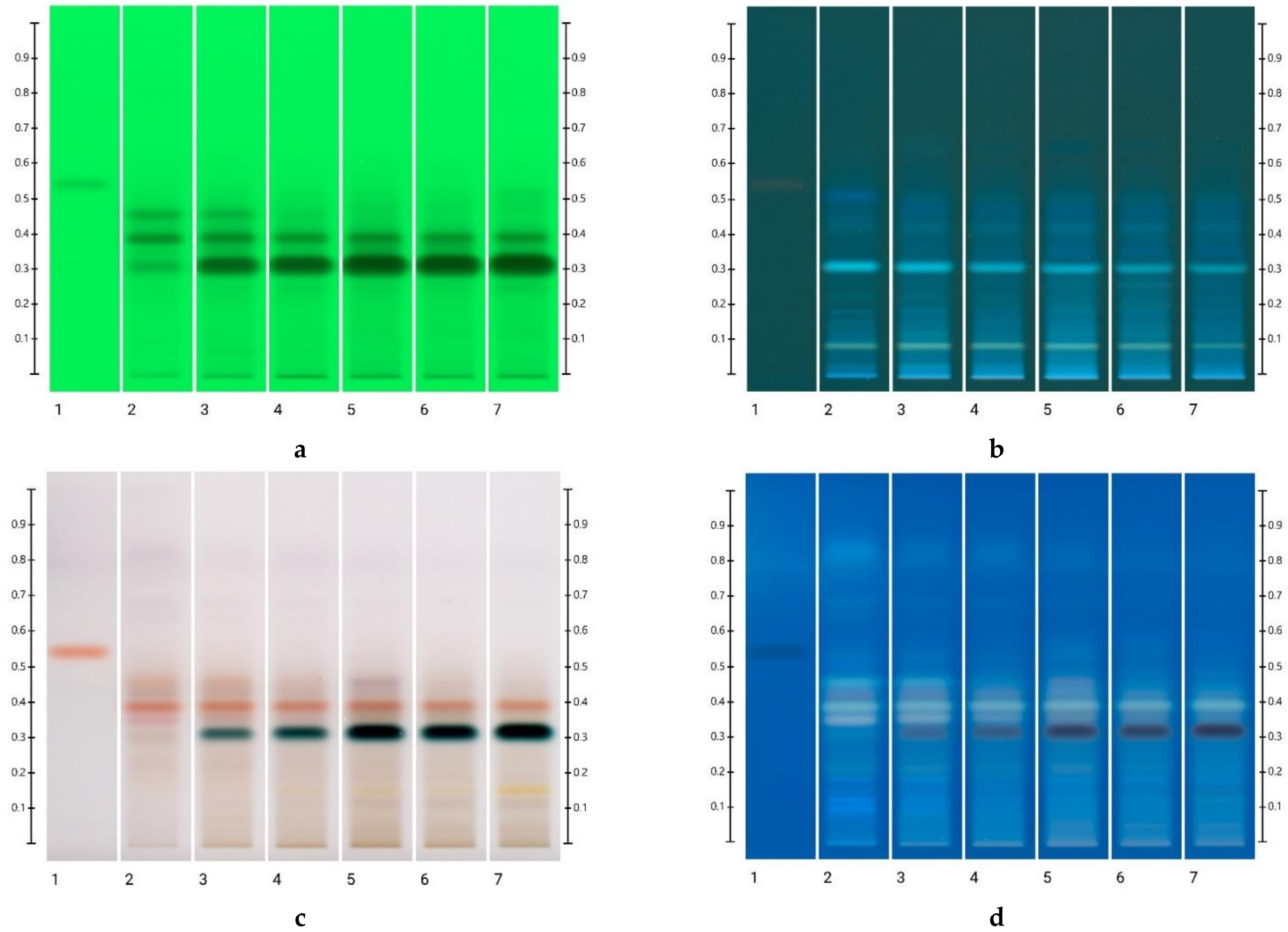

Supplementary Figure S35. MAR long-term storage at 60 °C Images taken at (**a**) 254 nm; (**b**) 366 nm; (**c**) White light after derivatisation and (**d**) 366 nm after derivatisation with vanillin reagent; Track 1—4,5,7-trihydroxyflavanon, Track 2— 0 h, Track 3— 1 month, Track 4— 2 month, Track 5— 3 month, Track 6— 4 month, and Track 7— 5 month; 5 µL of each honey extract respectively.

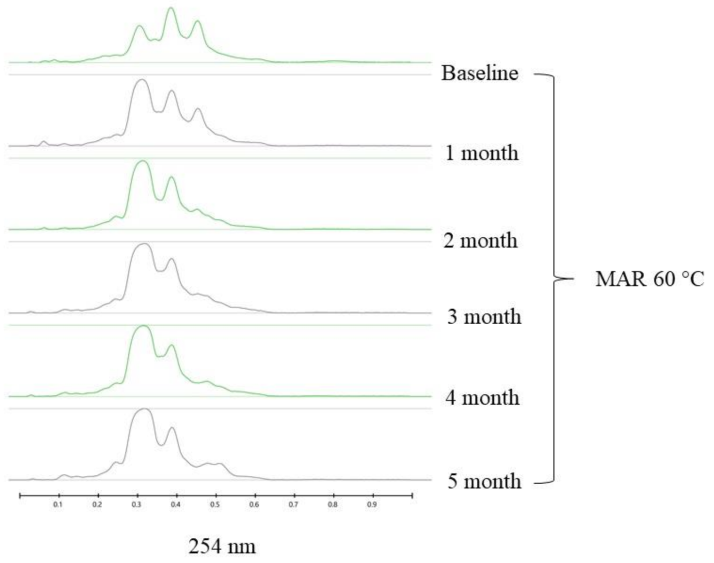

Supplementary Figure S36. Organic honey extract chromatograms (offset Y-axis values).

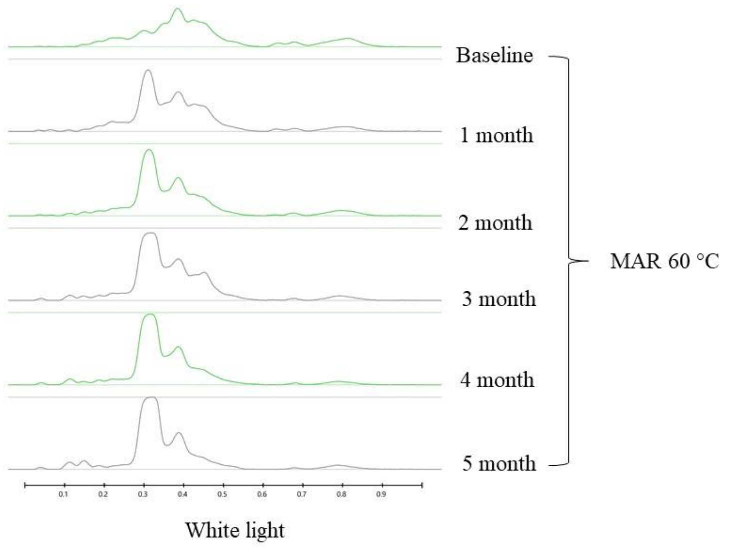

Supplementary Figure S37. Organic honey extract chromatograms (offset Y-axis values).

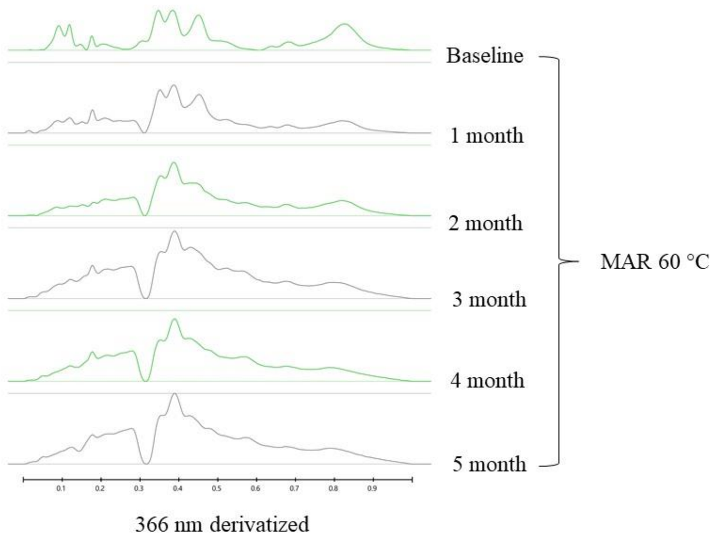

Supplementary Figure S38. Organic honey extract chromatograms (offset Y-axis values).

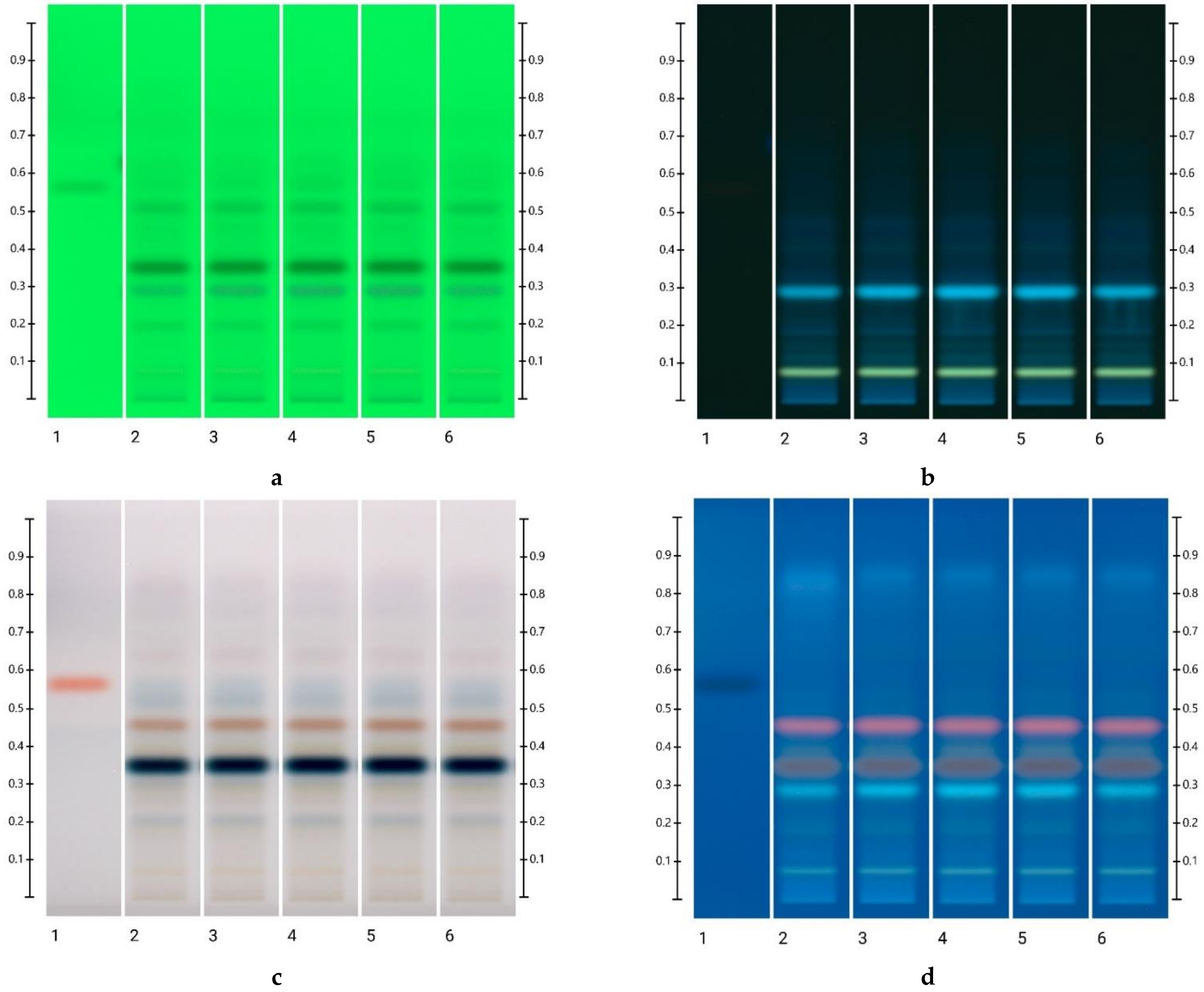

Supplementary Figure S39. PEP short-term storage at 40 °C; images taken at (**a**) 254 nm; (**b**) 366 nm; (**c**) White light after derivatisation and (**d**) 366 nm after derivatisation with vanillin reagent; Track 1— 4,5,7-trihydroxyflavanon, Track 2— 0 h, Track 3— 6 h, Track 4— 12 h, Track 5— 24 h, and Track 6— 48 h; 5 µL of each honey extract respectively.

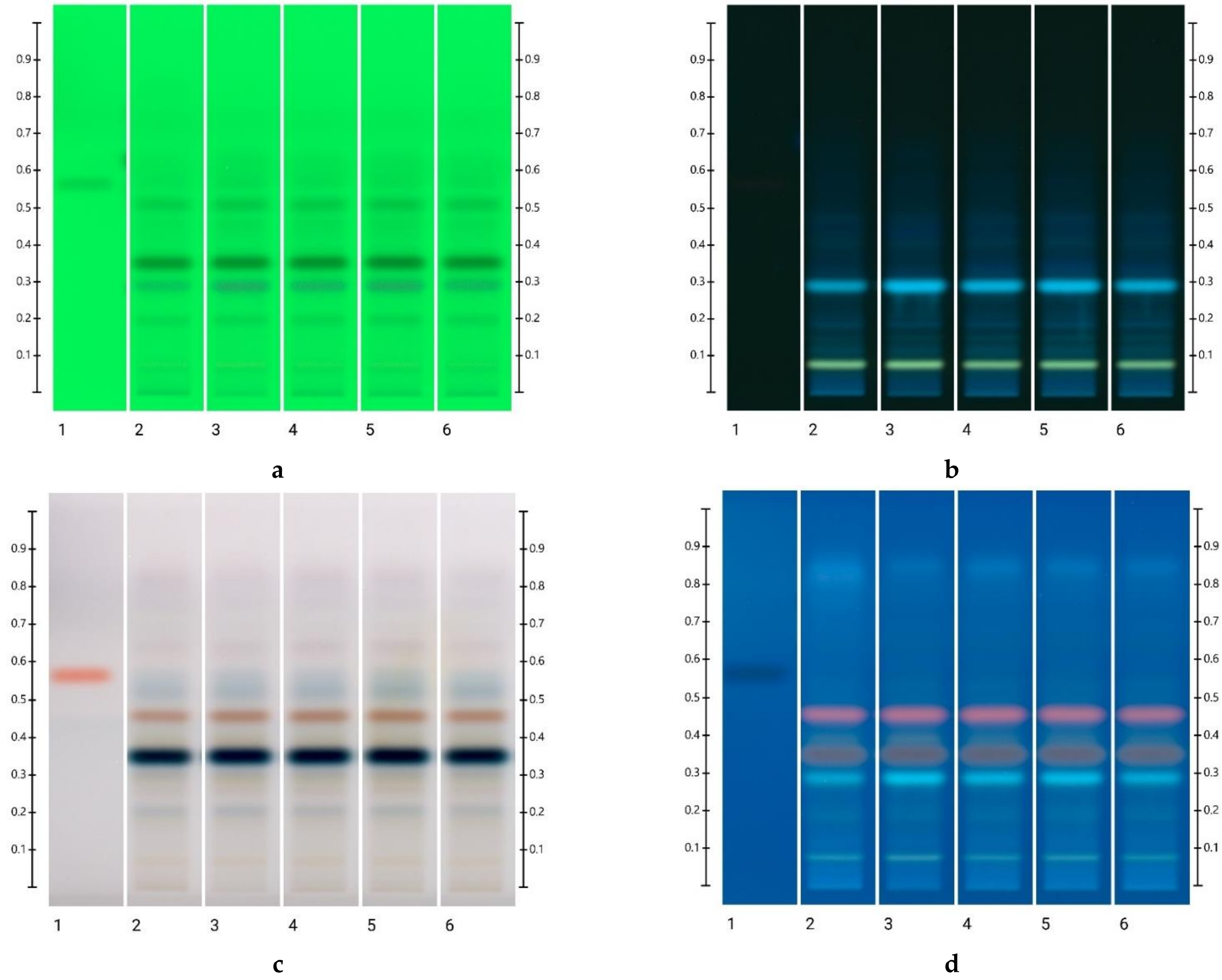

Supplementary Figure S40. PEP short-term storage at 60 °C; images taken at (**a**) 254 nm; (**b**) 366 nm; (**c**) White light after derivatisation and (**d**) 366 nm after derivatisation with vanillin reagent; Track 1— 4,5,7-trihydroxyflavanon, Track 2— 0 h, Track 3— 6 h, Track 4— 12 h, Track 5— 24 h, and Track 6— 48 h; 5 µL of each honey extract respectively.

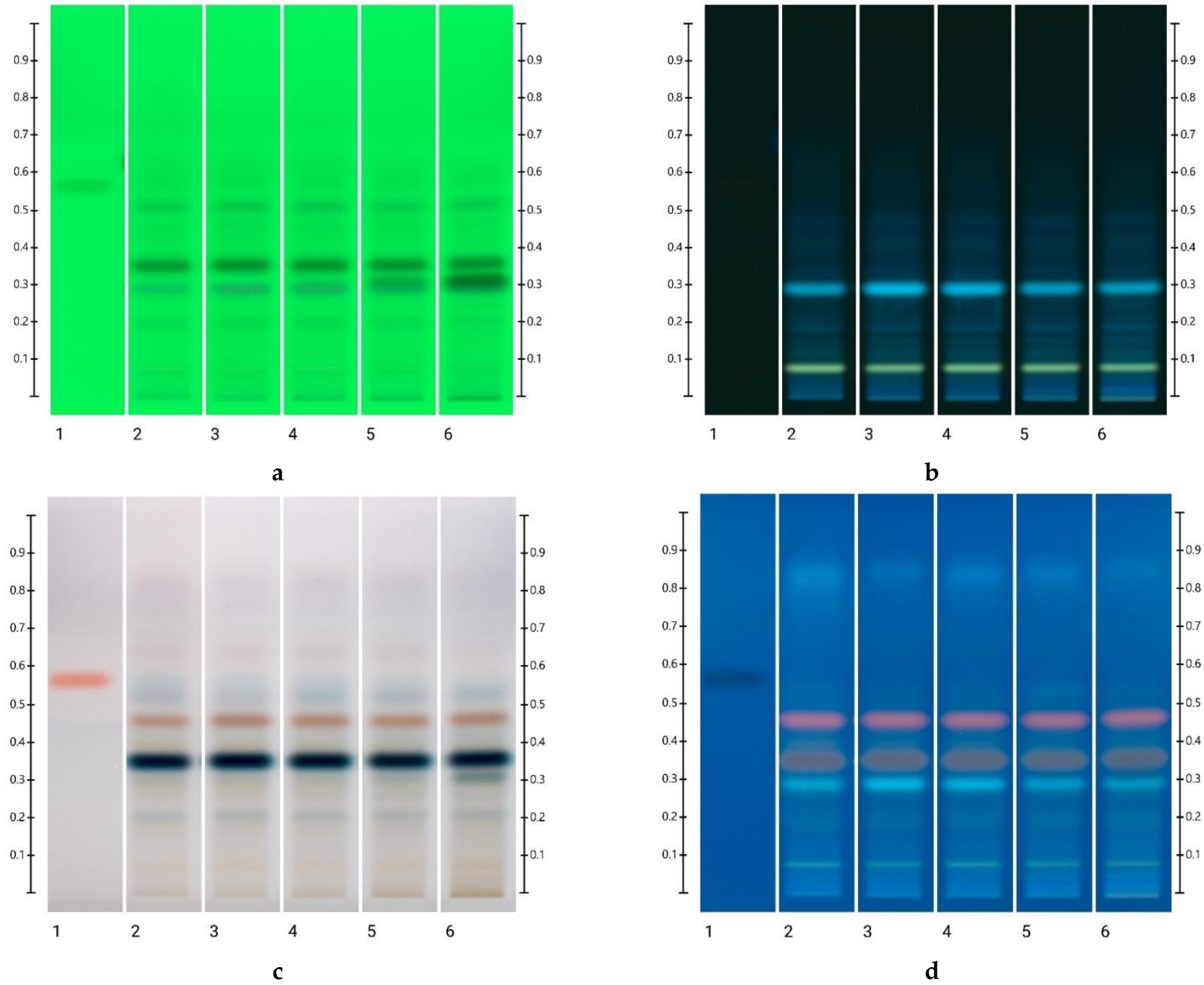

Supplementary Figure S41. PEP short-term storage at 80 °C; images taken at (**a**) 254 nm; (**b**) 366 nm; (**c**) White light after derivatisation and (**d**) 366 nm after derivatisation with vanillin reagent; Track 1— 4,5,7-trihydroxyflavanon, Track 2— 0 h, Track 3— 6 h, Track 4— 12 h, Track 5— 24 h, and Track 6— 48 h; 5 µL of each honey extract respectively.

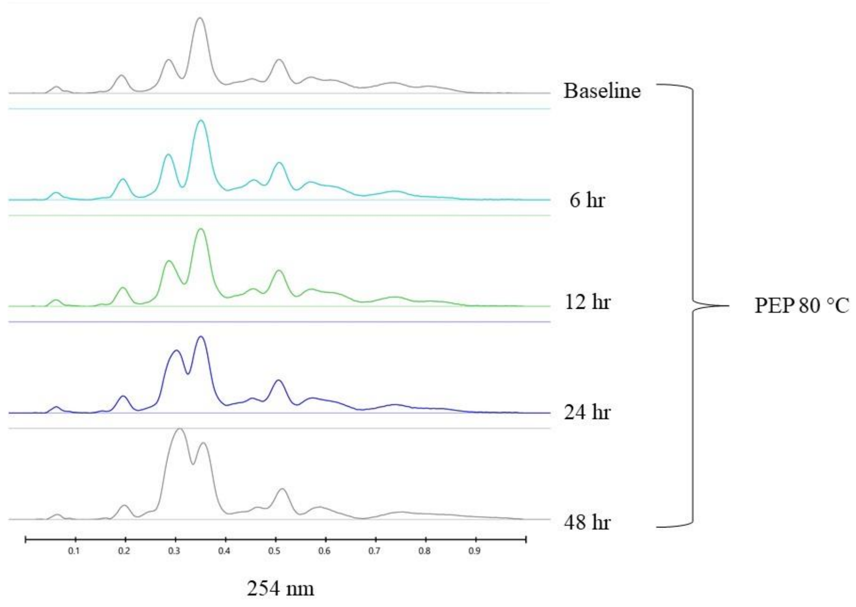

Supplementary Figure S42. Organic honey extract chromatograms (offset Y-axis values).

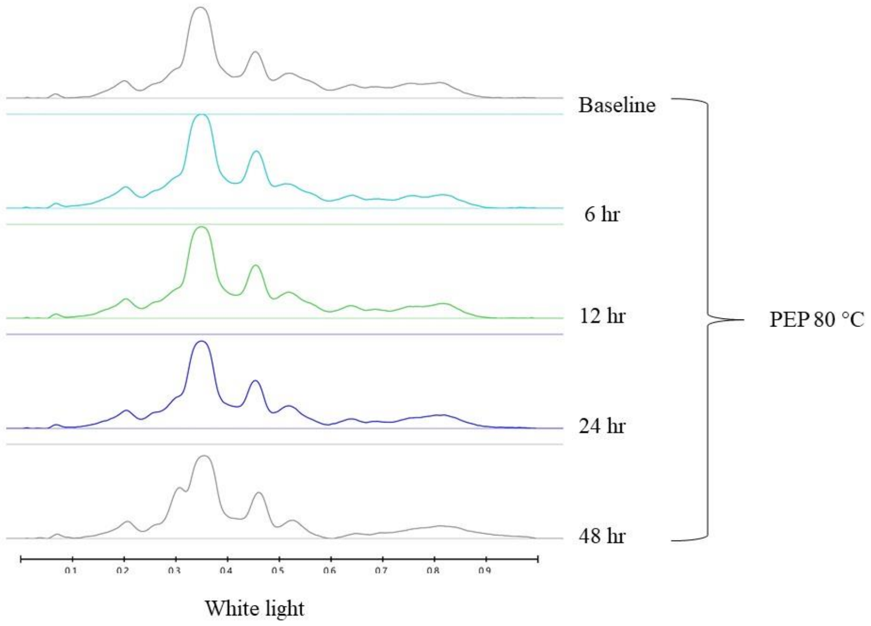

Supplementary Figure S43. Organic honey extract chromatograms (offset Y-axis values).

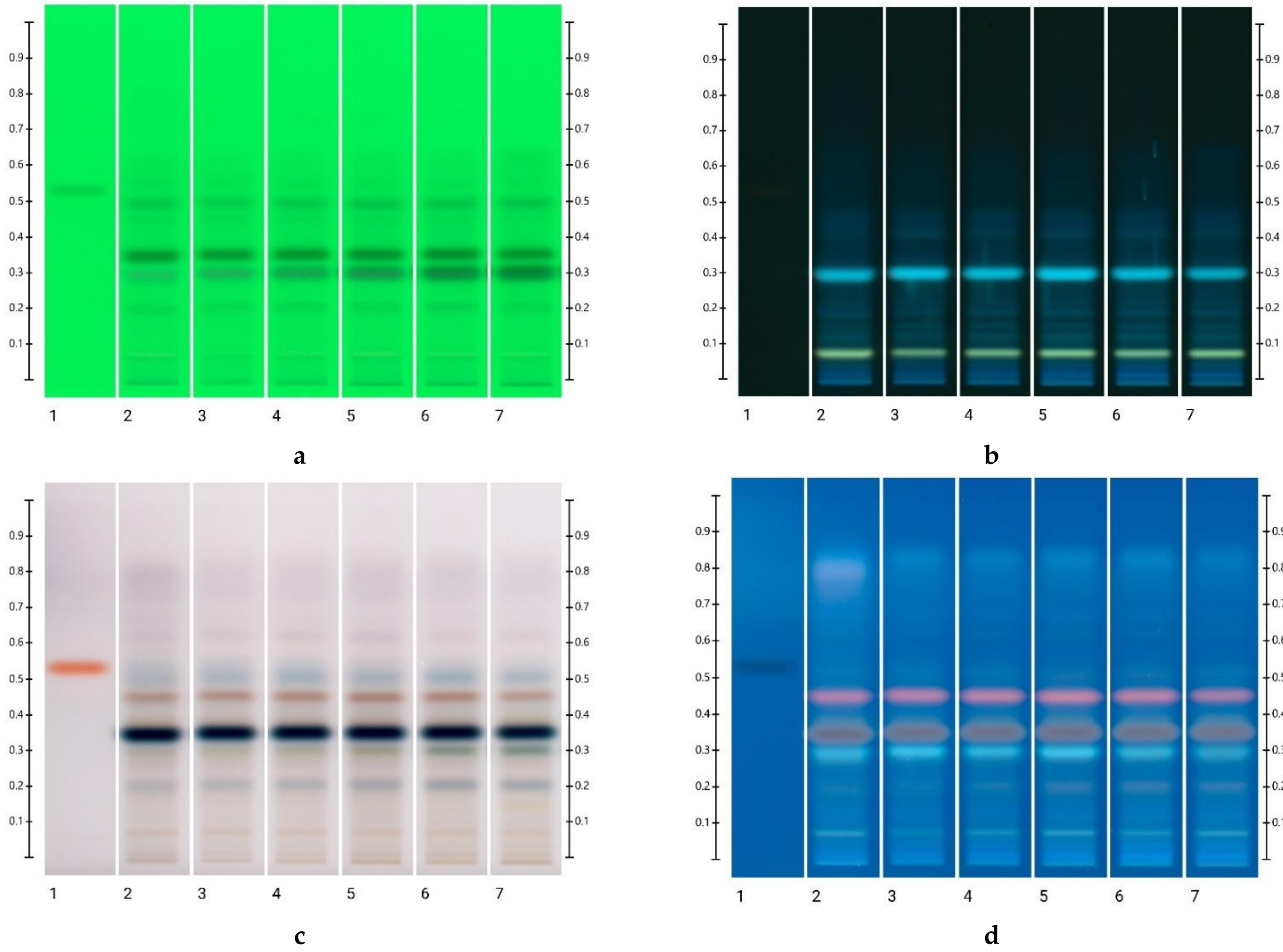

Supplementary Figure S44. PEP long-term storage at 40 °C Images taken at (**a**) 254 nm; (**b**) 366 nm; (**c**) White light after derivatisation and (**d**) 366 nm after derivatisation with vanillin reagent; Track 1—4,5,7-trihydroxyflavanon, Track 2— 0 h, Track 3— 1 month, Track 4— 2 month, Track 5— 3 month, Track 6— 4 month, and Track 7— 5 month; 5 µL of each honey extract respectively.

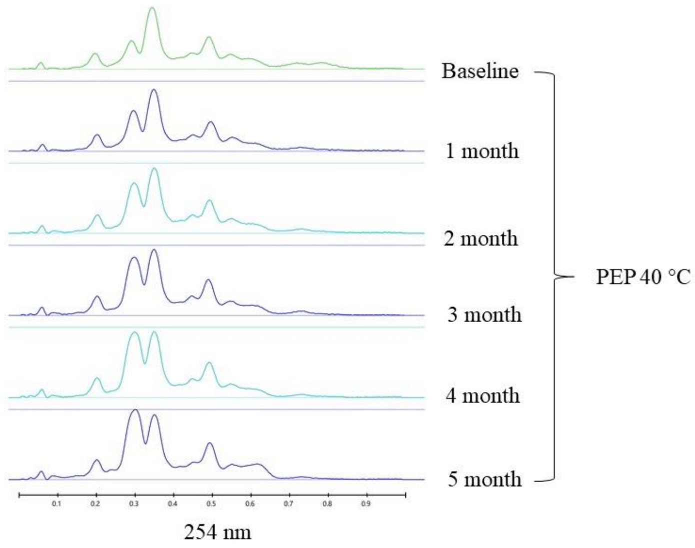

Supplementary Figure S45. Organic honey extract chromatograms (offset Y-axis values).

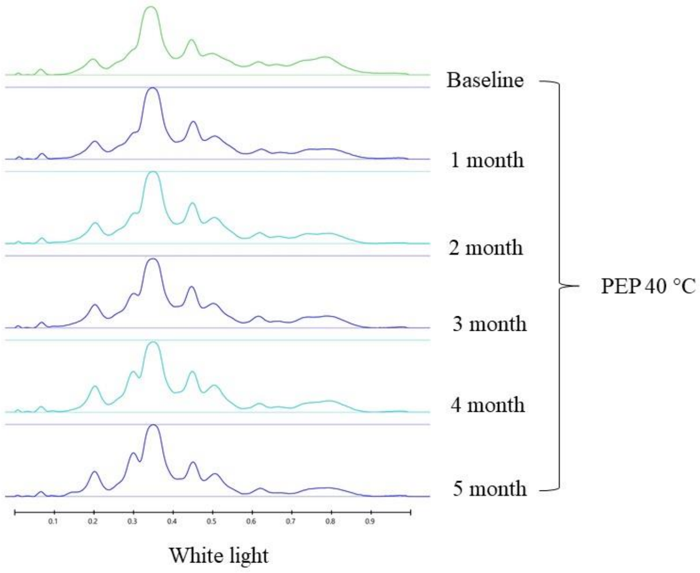

Supplementary Figure S46. Organic honey extract chromatograms (offset Y-axis values).

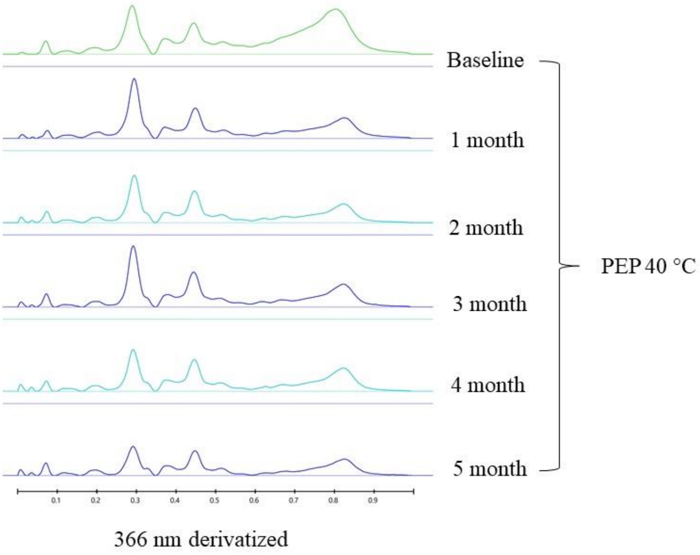

Supplementary Figure S47. Organic honey extract chromatograms (offset Y-axis values).

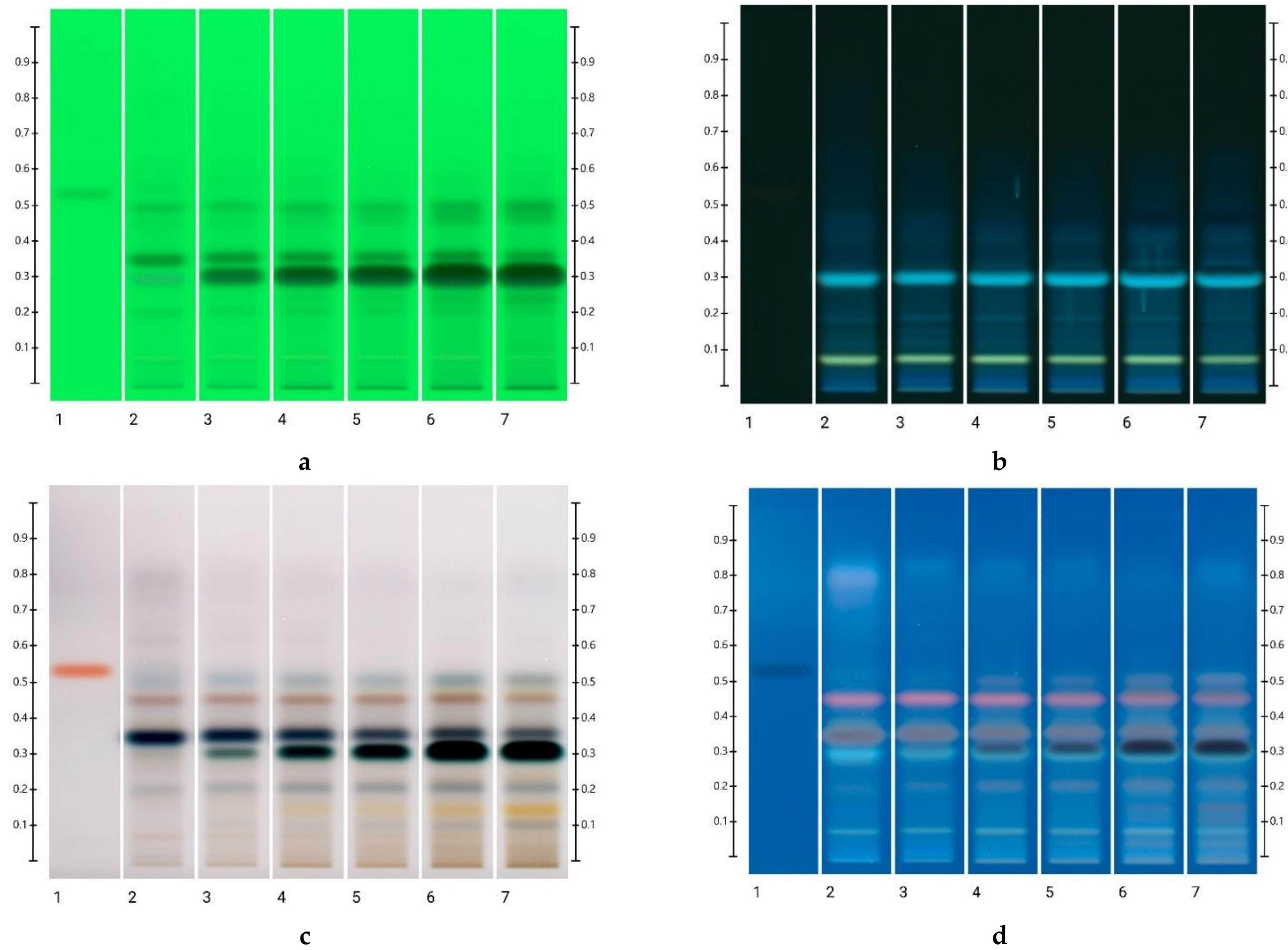

Supplementary Figure S48. PEP long-term storage at 60 °C Images taken at (**a**) 254 nm; (**b**) 366 nm; (**c**) White light after derivatisation and (**d**) 366 nm after derivatisation with vanillin reagent; Track 1—4,5,7-trihydroxyflavanon, Track 2— 0 h, Track 3— 1 month, Track 4— 2 month, Track 5— 3 month, Track 6— 4 month, and Track 7— 5 month; 5 µL of each honey extract respectively.

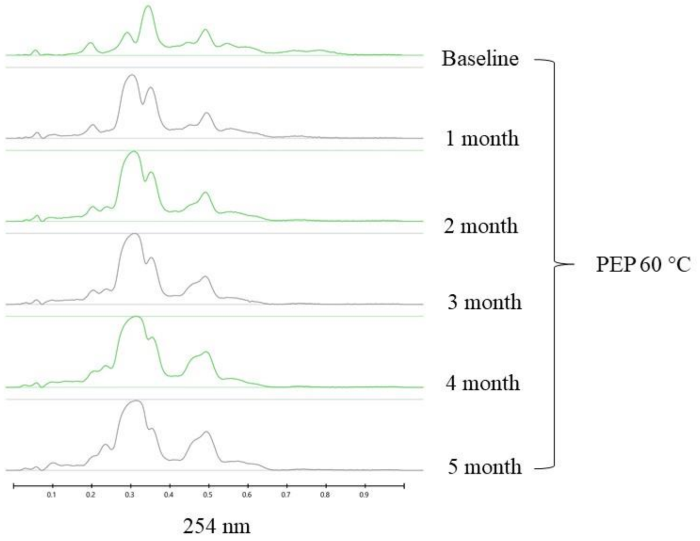

Supplementary Figure S49. Organic honey extract chromatograms (offset Y-axis values).

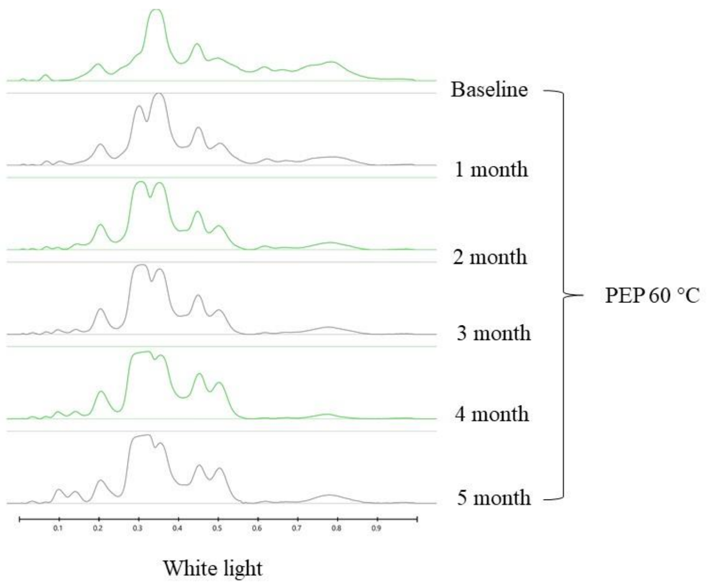

Supplementary Figure S50. Organic honey extract chromatograms (offset Y-axis values).

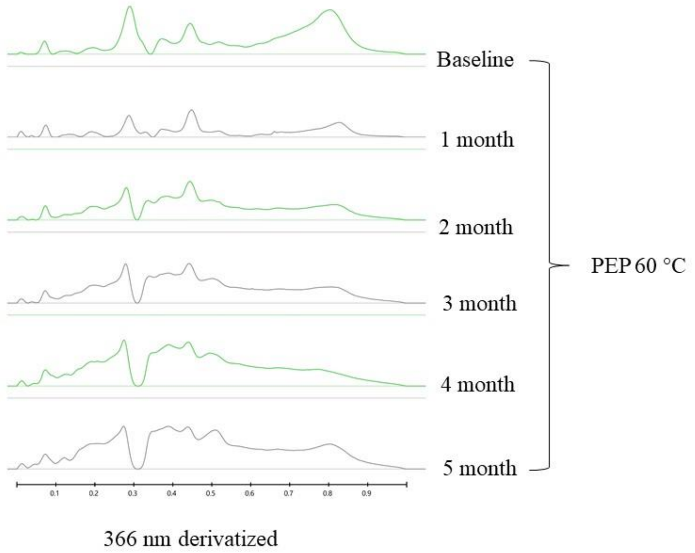

Supplementary Figure S51. Organic honey extract chromatograms (offset Y-axis values).

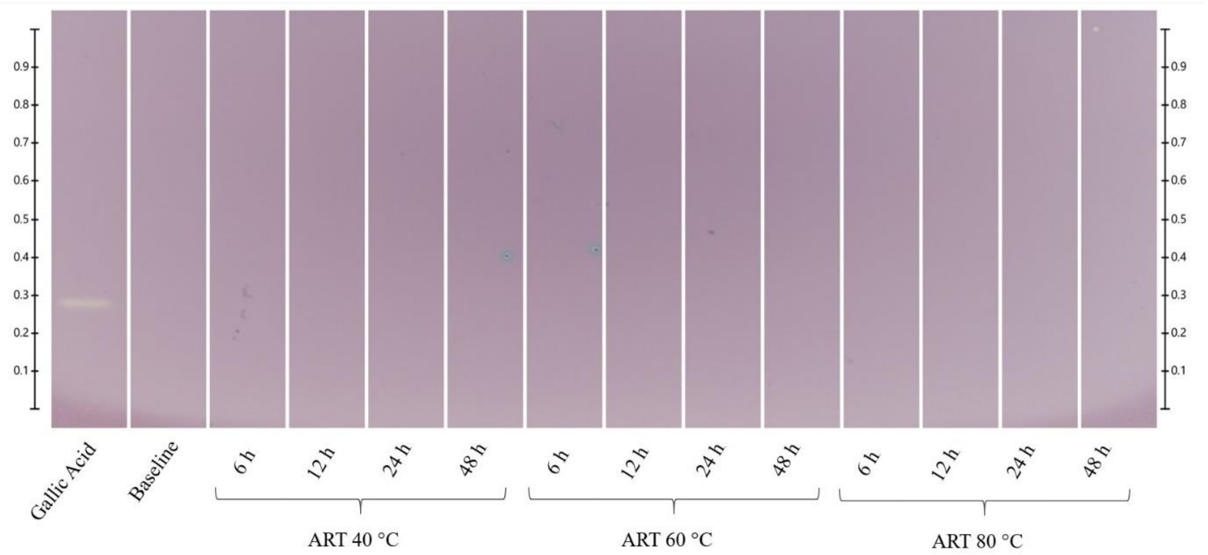

Supplementary Figure S52. HPTLC-DPPH fingerprints of ART stored at 40 °C, 60 °C and 80 °C for up to 48 h. Images of HPTLC plate were taken at White light after 60 min of derivatization with DPPH* reagents; Gallic acid (4 µL) and honey extracts (5 µL) respectively.

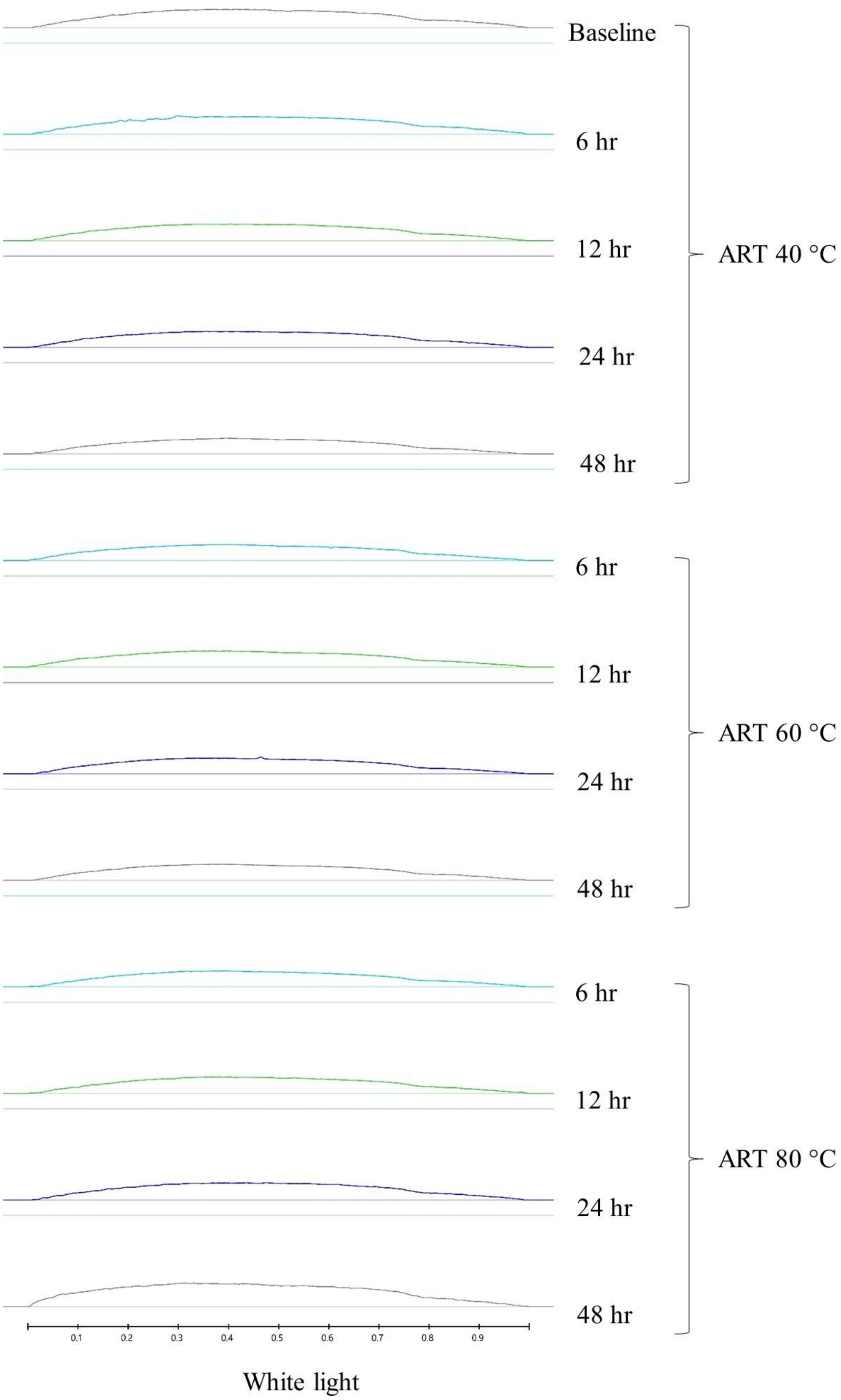

Supplementary Figure S53. Organic honey extract chromatograms (offset Y-axis values).

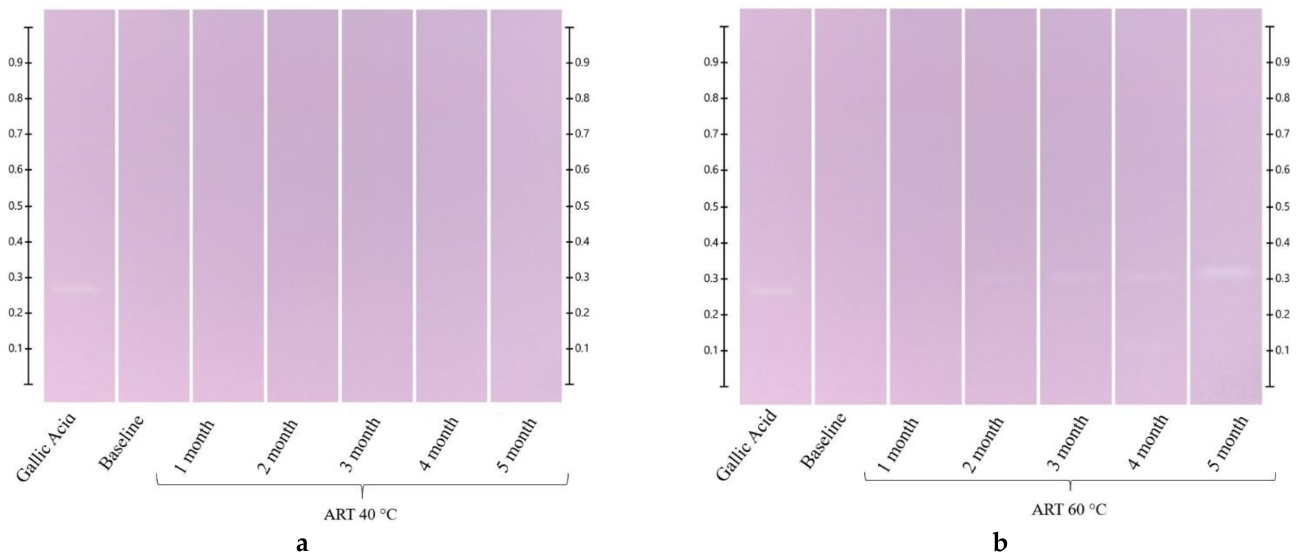

Supplementary Figure S54. HPTLC-DPPH fingerprints of ART stored at 40 °C (**a**), and at 60 °C (**b**) for up to 5 months. Images of HPTLC plate were taken at White light after 60 min of derivatization with DPPH* reagents; Gallic acid (4 μL) and honey extracts (5 μL) respectively.

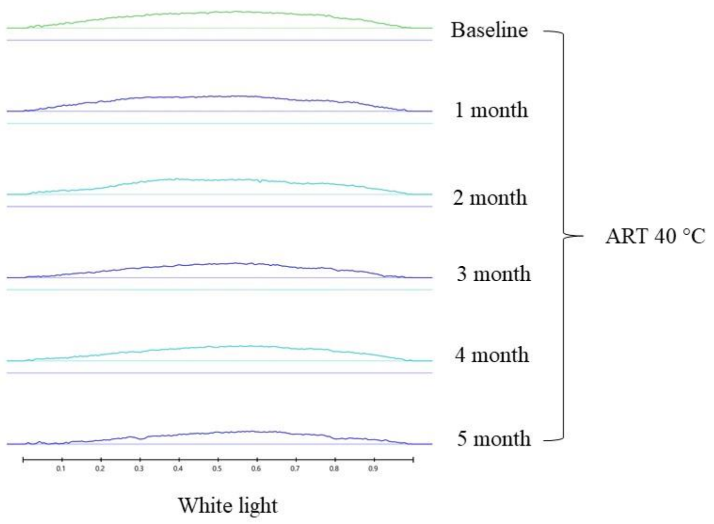

Supplementary Figure S55. Organic honey extract chromatograms (offset Y-axis values).

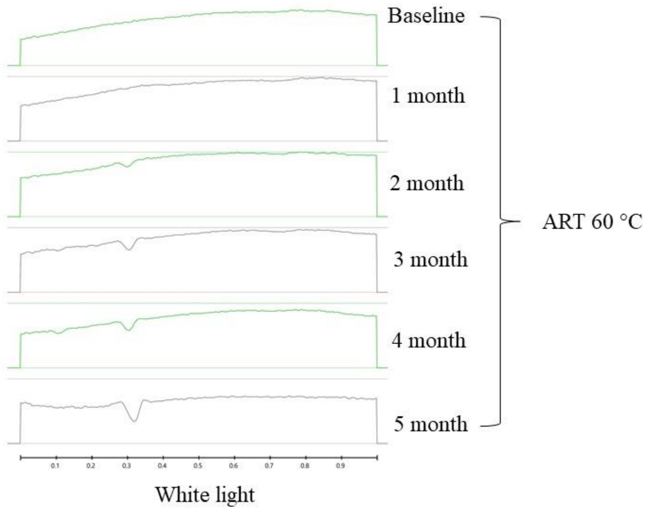

Supplementary Figure S56. Organic honey extract chromatograms (offset Y-axis values).

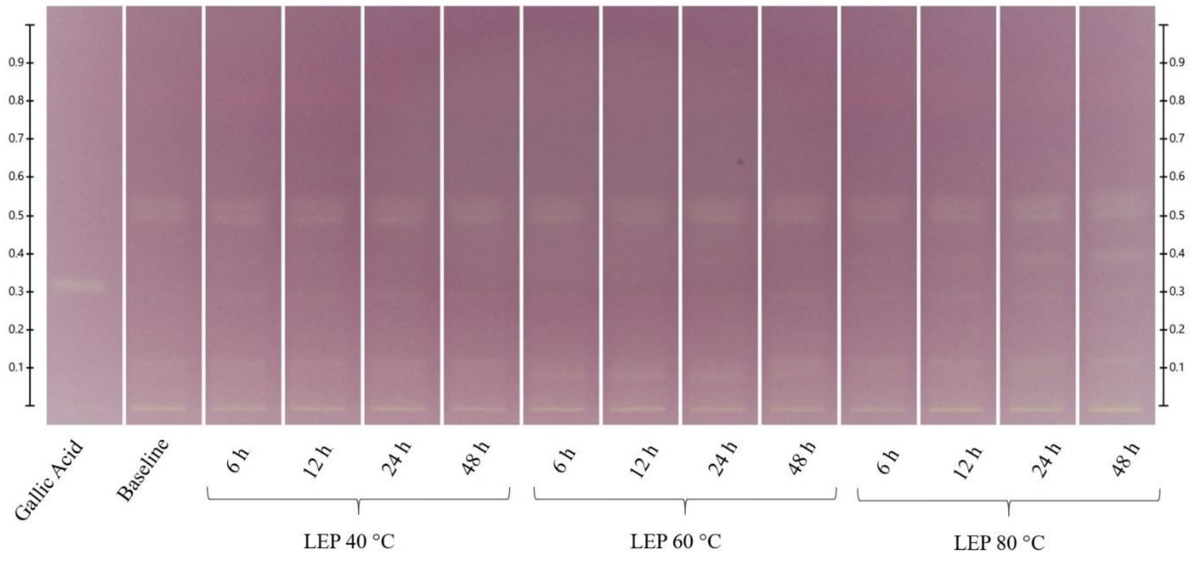

Supplementary Figure S57. HPTLC-DPPH fingerprints of LEP honey stored at 40 °C, 60 °C and 80 °C for up to 48 hours. Images of HPTLC plate were taken at White light after 60 min of derivatization with DPPH* reagents; Gallic acid (4 μL) and honey extracts (5 μL) respectively.

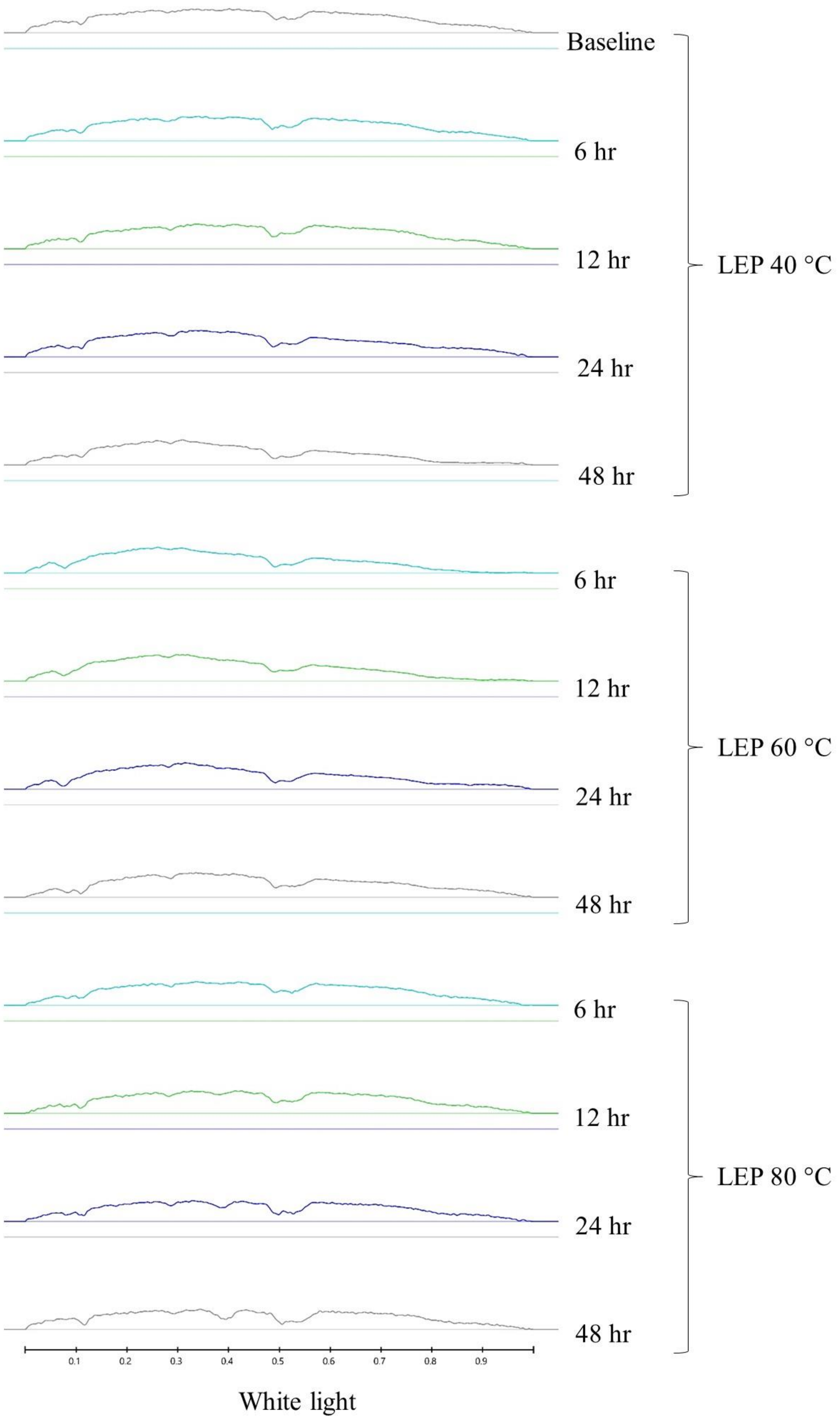

Supplementary Figure S58. Organic honey extract chromatograms (offset Y-axis values).

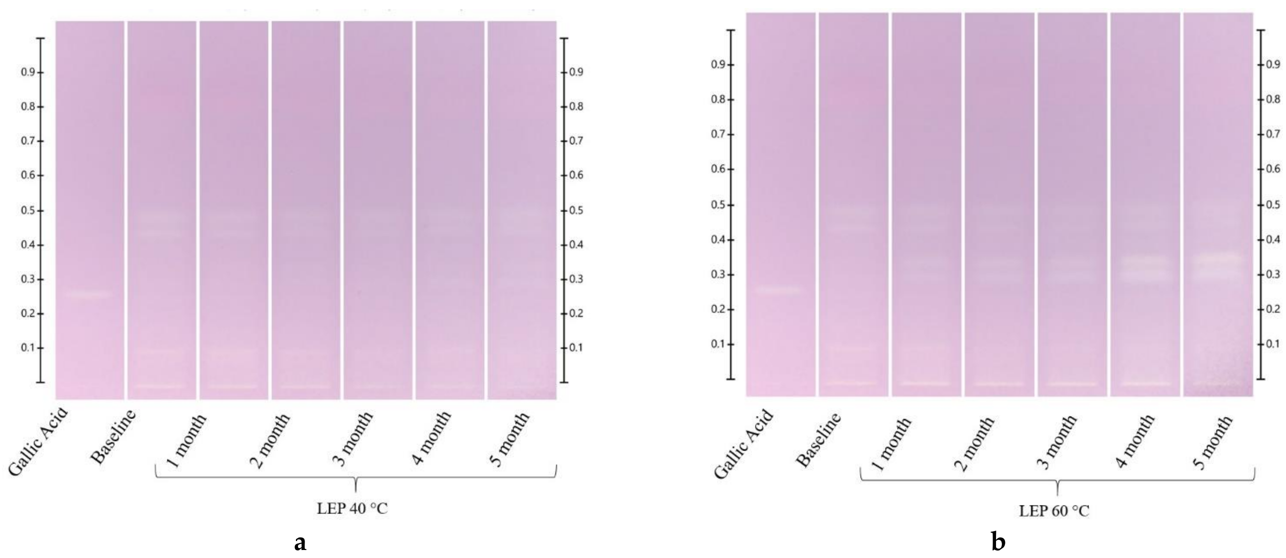

Supplementary Figure S59. HPTLC-DPPH fingerprints of LEP honey stored at 40 °C (**a**), and at 60 °C (**b**) for up to 5 months. Images of HPTLC plate were taken at White light after 60 min of derivatization with DPPH* reagents; Gallic acid (4 µL) and honey extracts (5 µL) respectively.

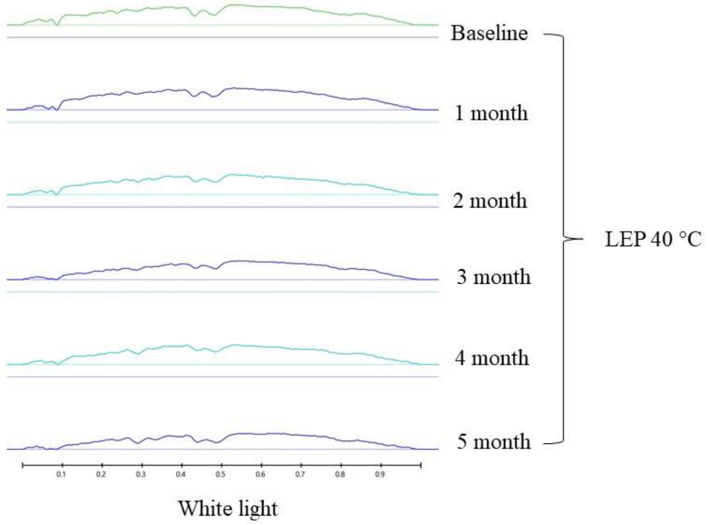

Supplementary Figure S60. Organic honey extract chromatograms (offset Y-axis values).

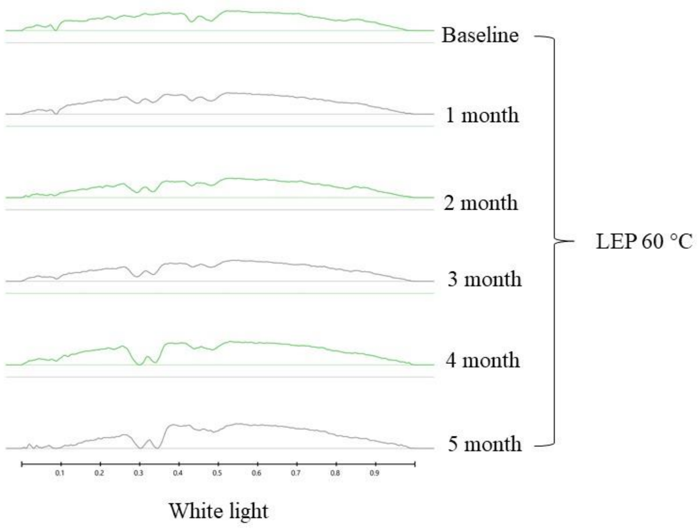

Supplementary Figure S61. Organic honey extract chromatograms (offset Y-axis values).

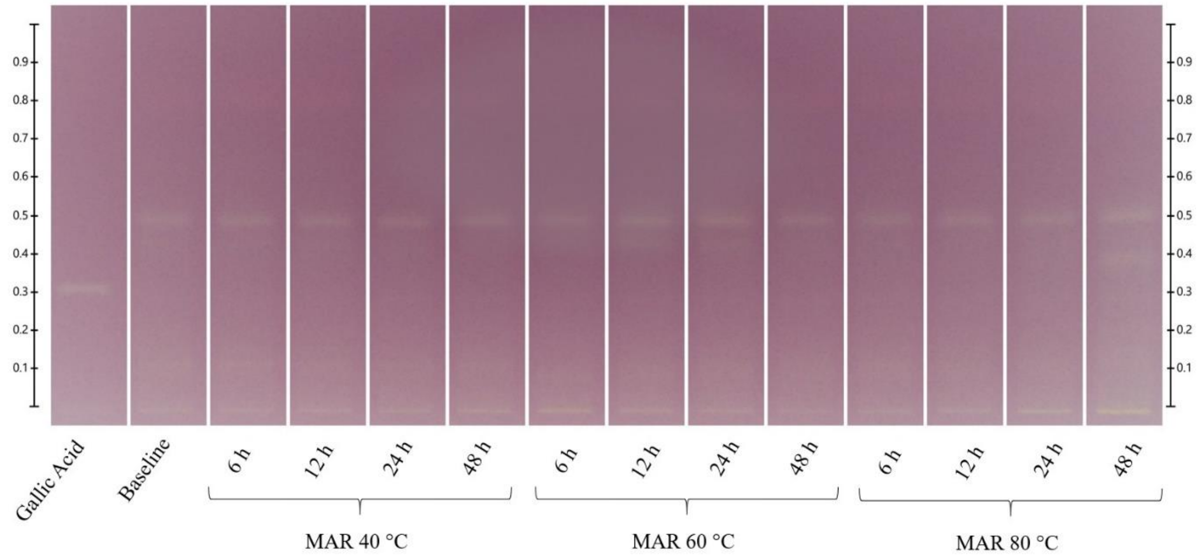

Supplementary Figure S62. HPTLC-DPPH fingerprints of MAR stored at 40 °C, 60 °C and 80 °C for up to 48 hours. Images of HPTLC plate were taken at White light after 60 min of derivatization with DPPH* reagents; Gallic acid (4 μL) and honey extracts (5 μL) respectively.

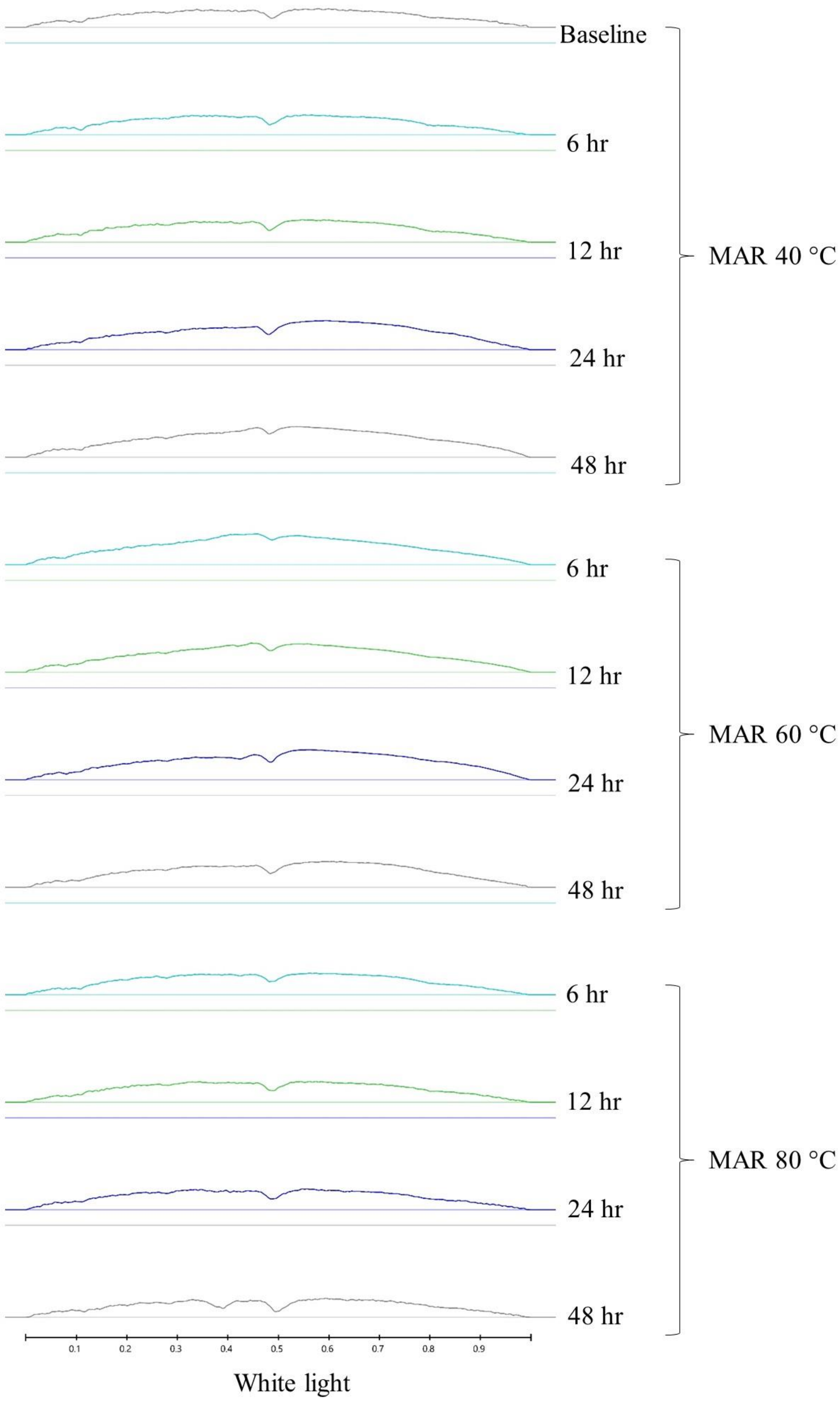

Supplementary Figure S63. Organic honey extract chromatograms (offset Y-axis values).

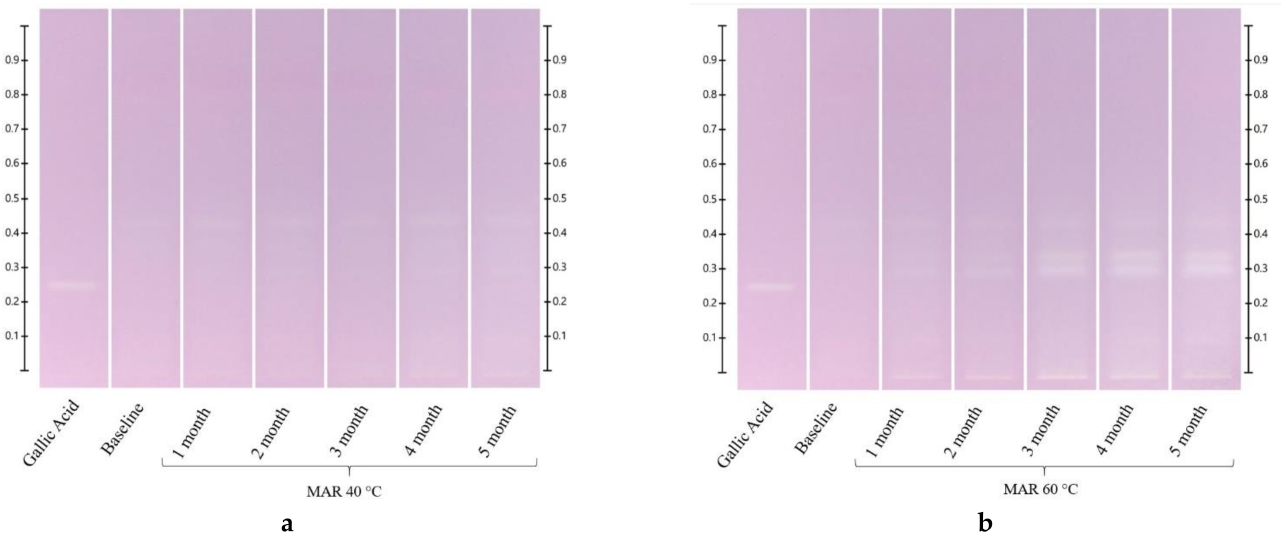

Supplementary Figure S64. HPTLC-DPPH fingerprints of MAR honey stored at 40 °C (**a**), and at 60 °C (**b**) for up to 5 months. Images of HPTLC plate were taken at White light after 60 min of derivatization with DPPH* reagents; Gallic acid (4 μL) and honey extracts (5 μL) respectively.

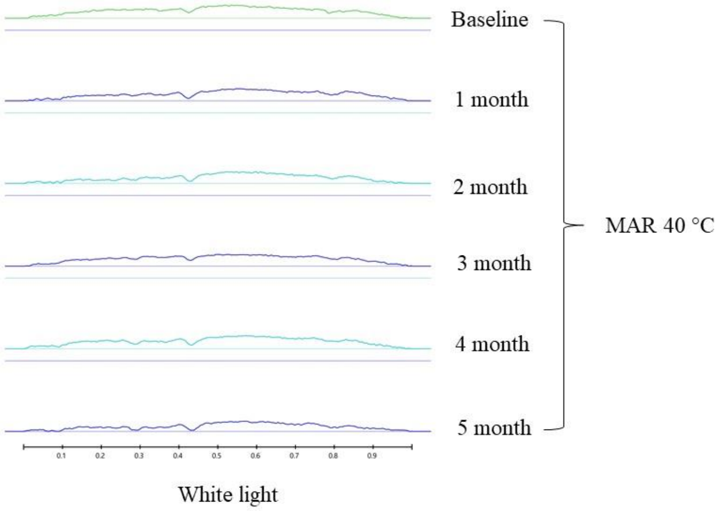

Supplementary Figure S65. Organic honey extract chromatograms (offset Y-axis values).

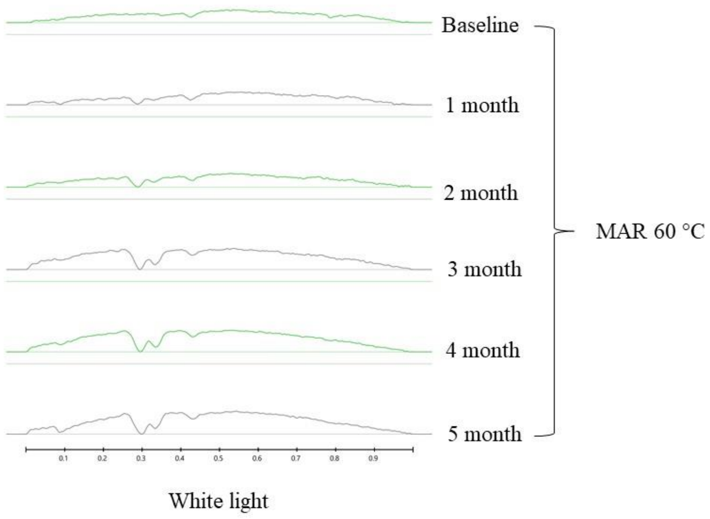

Supplementary Figure S66. Organic honey extract chromatograms (offset Y-axis values).

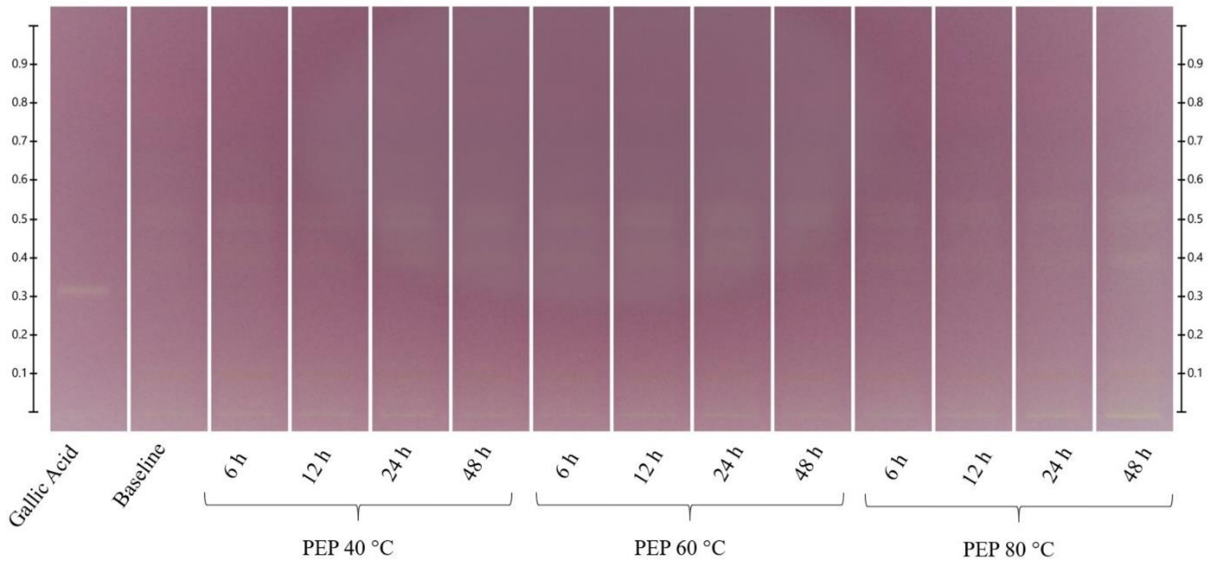

Supplementary Figure S67. HPTLC-DPPH fingerprints of PEP stored at 40 °C, 60 °C and 80 °C for up to 48 hours. Images of HPTLC plate were taken at White light after 60 min of derivatization with DPPH* reagents; Gallic acid (4 μL) and honey extracts (5 μL) respectively.

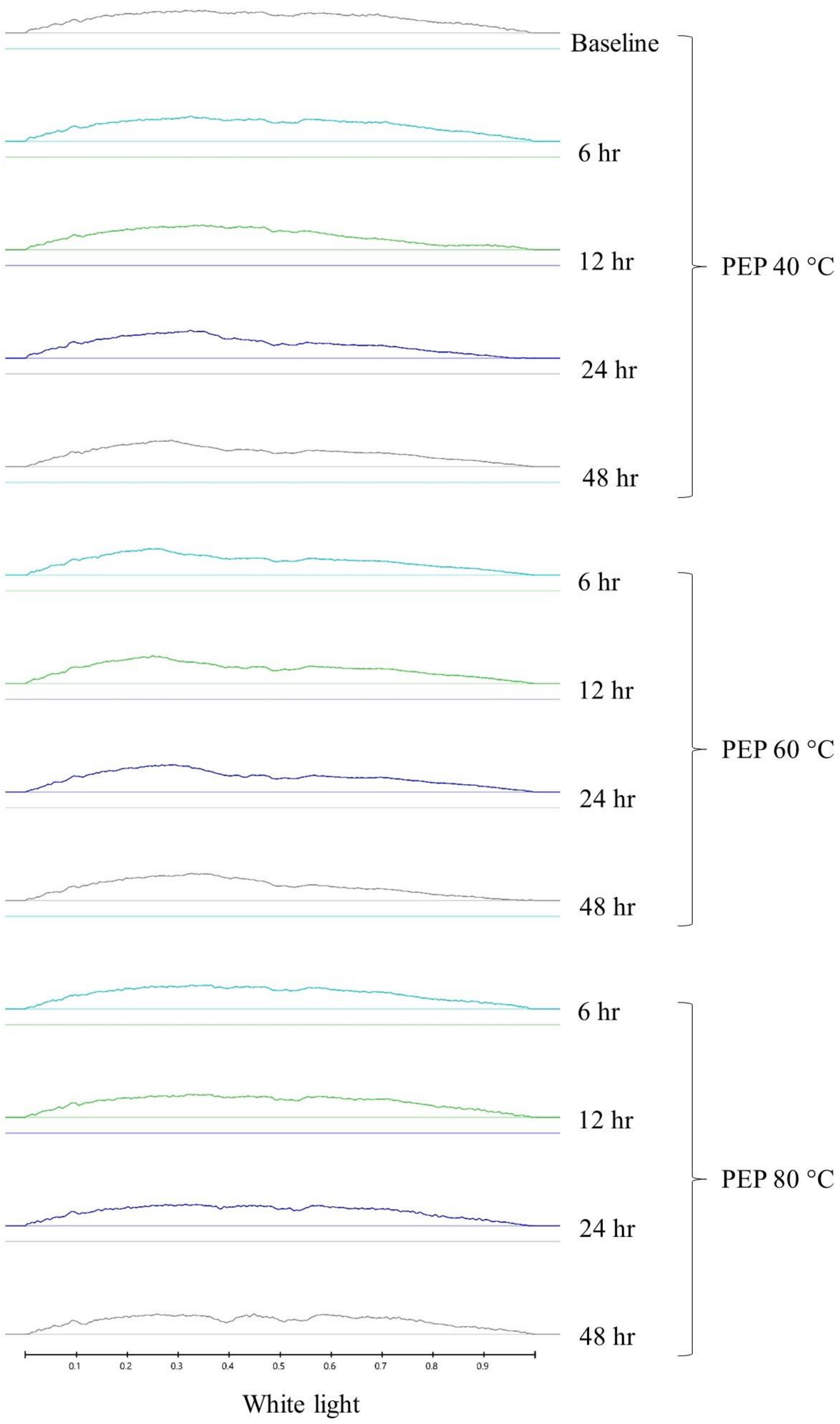

Supplementary Figure S68. Organic honey extract chromatograms (offset Y-axis values).

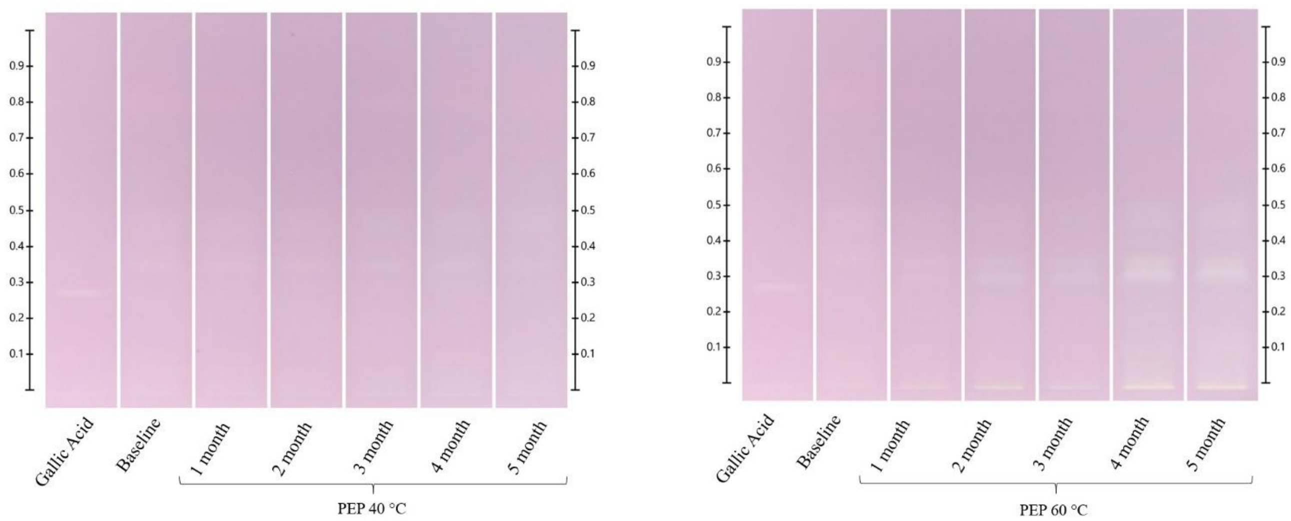

a **b**

Supplementary Figure S69. HPTLC-DPPH fingerprints of PEP honey stored at 40 °C (**a**), and at 60 °C (**b**) for up to 5 months. Images of HPTLC plate were taken at White light after 60 min of derivatization with DPPH* reagents; Gallic acid (4 μL) and honey extracts (5 μL) respectively.

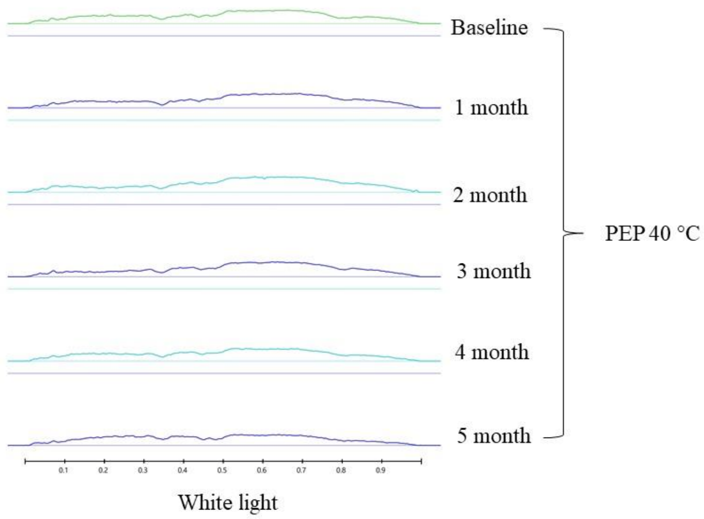

Supplementary Figure S70. Organic honey extract chromatograms (offset Y-axis values).

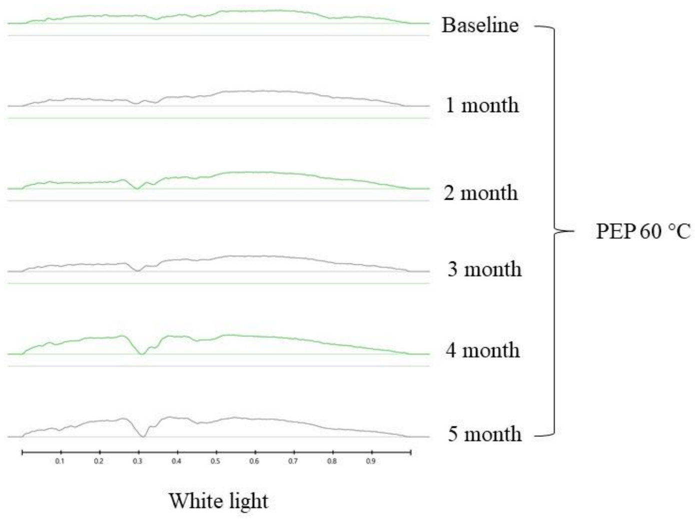

Supplementary Figure S71. Organic honey extract chromatograms (offset Y-axis values).
